# Ladder-Type Cu(II) Coordination Polymer with π–π Stacking of Planar Blatter Radical Ligands: Structural and Magnetic Characterization

**DOI:** 10.1021/acs.cgd.5c01115

**Published:** 2025-09-17

**Authors:** Hemant K. Singh, Kayla M. Smith, Jeremy M. Rawson, Bruno Camargo, Ethan S. Pollett, Oleksandr Hietsoi, Andrienne C. Friedli, Piotr Kaszyński

**Affiliations:** † Centre of Molecular and Macromolecular Studies, 86897 Polish Academy of Sciences, 90363 Łódź, Poland; ‡ Department of Chemistry, 5235 Middle Tennessee State University, Murfreesboro, Tennessee 37132, United States; § Department of Chemistry and Biochemistry, 8637 University of Windsor, Windsor, Ontario N9B 3P4, Canada; ∥ Institute of Experimental Physics, Faculty of Physics, 226285 University of Warsaw, 02093 Warsaw, Poland; ⊥ Faculty of Chemistry, University of Łódź, 91403 Łódź, Poland

## Abstract

The synthesis of two C(2)-pyridyl derivatives of the planar Blatter radical was developed by using the tris­(trimethylsilyl)­silane (TTMSS)-assisted cyclization of appropriate iodoarenes. These monodentate paramagnetic ligands were characterized by spectroscopic (UV–vis and electron paramagnetic resonance (EPR)) and electrochemical methods and reacted with (pdc)­Cu­(H_2_O)_3_. A complex containing the 3-pyridyl group was characterized structurally, revealing novel polymeric Cu–O–Cu–O ladders separated with slipped stacks of radical ligands. Superconducting quantum interference device (SQUID) magnetometry of this complex demonstrated strong antiferromagnetic interactions between the radicals and largely isolated Cu­(II) ions. Analysis of the magnetic data with the Hatfield model, assuming two isolated one-dimensional (1D) alternating Heisenberg chains, gave *J*_RR_/*k*_B_ = −1200 K and α_RR_ = 0.4 for the paramagnetic ligand stacks, and *J*_CuCu_/*k*_B_ = −3.5 K and α_CuCu_ = 0.9 for the Cu­(II) ion chains. Analysis of the experimental data was augmented with density functional theory (DFT) calculations.

## Graphical abstract

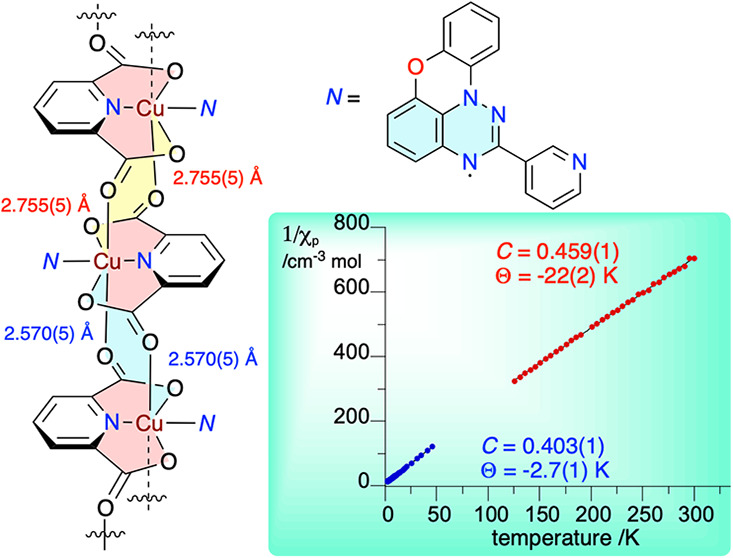

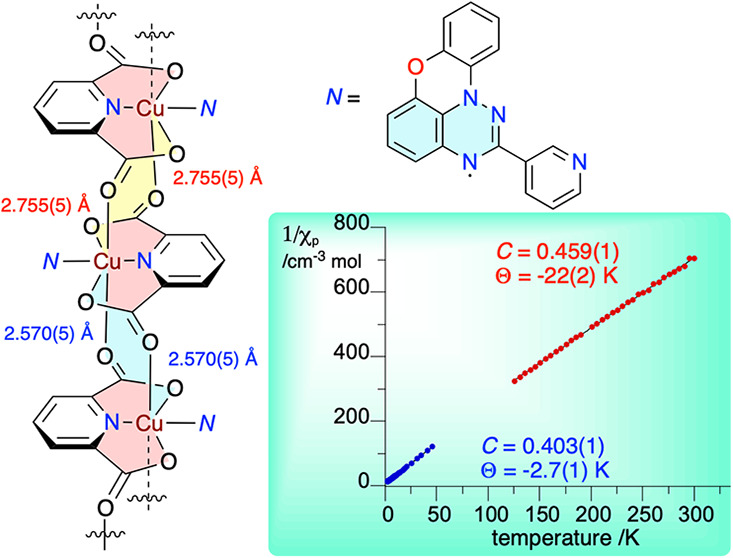

## Introduction

Metal ion complexes with organic ligands constitute an important class of functional materials, with applications in catalysis,^1^ photonics,^2,3^ electrochromics,^4^ electron conduction,^5–7^ sensing,^8,9^ and magnetism.^6,10,11^ The supramolecular structure and function of such materials can be controlled in a wide range of behaviors by the judicious choice of ligands.^12^ Besides molecular materials, in which each complex is an individual species, complexes can form coordination polymer chains,^13–15^ sheets,^7,16^ and three-dimensional (3-D) structures,^17^ such as metal–organic frameworks (MOFs).^11,18–21^

A particularly interesting subset of metal ion complexes is that containing paramagnetic organic ligands (stable radicals) coordinated to paramagnetic metal ions. This so-called metal–radical approach^10,22^ results in magnetic materials, which are of general interest for basic science and information processing technologies.^23,24^ In this context, a number of open-shell ligands, e.g., semiquinones,^25,26^ nitroxides,^27^ thiazyls,^28,29^ and verdazyls,^30,31^ have been coordinated to metal ions. The use of di- and multitopic paramagnetic ligands leads to chains, sheets, and 3D structures with increased dimensionality of magnetic interactions.^32,33^ More recently, investigations have focused on complexes with ligands derived from Blatter radical **1a** (Figure 1).

**Figure 1:**
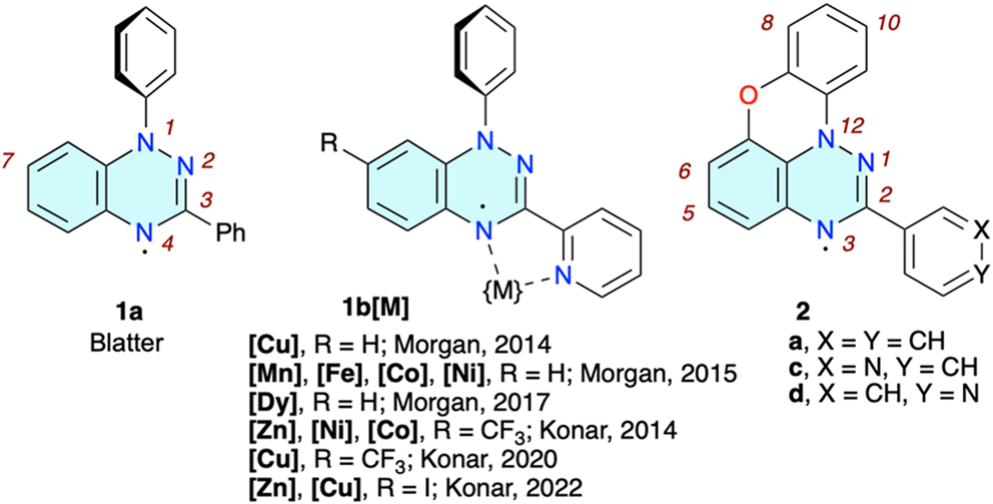
Structures and partial numbering system of Blatter radical **1a**, metal complexes of the C(3)-pyrid-2-yl derivative **1b**, and C(2)-aryl derivatives **2** of the planar Blatter radical.

Benzo­[*e*]­[1,2,4]­triazinyl radicals,^34^ including the prototypical Blatter radical **1a** ^35^ (Figure 1), constitute an extended family of exceptionally stable^36^ π-delocalized^37^ radicals with favorable electrochemical and photophysical properties.^38,39^ Their well-developed chemistry^40^ permits functionalization^41^ and incorporation into more complex molecular systems, such as liquid crystals.^42–44^

Initial studies concentrated on the C(3)-pyrid-2-yl derivative **1b** (R = H) as a ligand with a bidentate chelation pocket, similar to that in 2,2′-bipyridyl, for paramagnetic metal ion complexes. Results demonstrated that in **1b­[M]** (R = H, Figure 1) metal–radical exchange interactions are ferromagnetic for M = Cu­(II) and M = Ni­(II), while for the analogous complexes with M = Mn­(II), Fe­(II), and Dy­(III), the interactions between ions and radicals are antiferromagnetic.^45–47^ These investigations were later expanded to ligands **1b** with R = CF_3_ and I, for which relatively strong ferromagnetic M–radical interactions were found for complexes **1b­[Ni]**, **1b­[Co]**, and **1b­[Cu]**.^48–50^ In the complex **1b­[Zn]** with the diamagnetic Zn­(II) ion, only a weak through space antiferromagnetic exchange interaction, *J* = −3.5 cm^–1^, was observed between the radical centers.^50^ A summary of these findings is tabularized in ref 49.

The two ancillary ligands in the reported complexes of **1b** are the hexafluoacetylacetonate (hfac) and, in the case of **1b­[Dy]**, the di-*t*-butyl-propane-1,3-dionate (tbacac), which isolate the metal ions from each other. The size of these ligands, the ancillary and Blatter, permits only the formation of discrete π–π dimers with significant interplanar separations and, consequently, weak intermolecular exchange interactions.

A ligand that could provide an extended structure and higher dimensionality of magnetic interactions is the planar, tridentate pyridine-2,6-dicarboxylate (pdc), which enforces a square-planar geometry of M ions, such as Cu­(II), with one coplanar site open for binding with a monodentate heterocyclic N ligand (Figure 2). The two apical positions are available for ligands L that can connect the molecules through, e.g., H_2_O and intermolecular H-bonding^51,52^ or by coordination of a C=O group, which is either part of the heterocyclic ligand^53^ or the neighboring pdc fragment (Figure 2). While the first mode of formation of coordination chains, with a [–Cu­(RO)­H···O=CO–] motif and H-bonded networks with H_2_O, is reasonably well exemplified, the involvement of the carbonyl group is much less common.^51^ From a multidimensional materials perspective, the last scenario ([−Cu–O=CO−] motif in Figure 2) is particularly attractive because the (pdc)Cu units interact directly, leading to dimers,^54,55^ oligomers,^51,56^ and polymers with relatively short Cu···Cu separations.^57^ The close proximity of the Cu centers requires relatively flat ligands N, such as **2c** or **2d** (Figure 1), that can π-stack in this confined arrangement.

**Figure 2:**
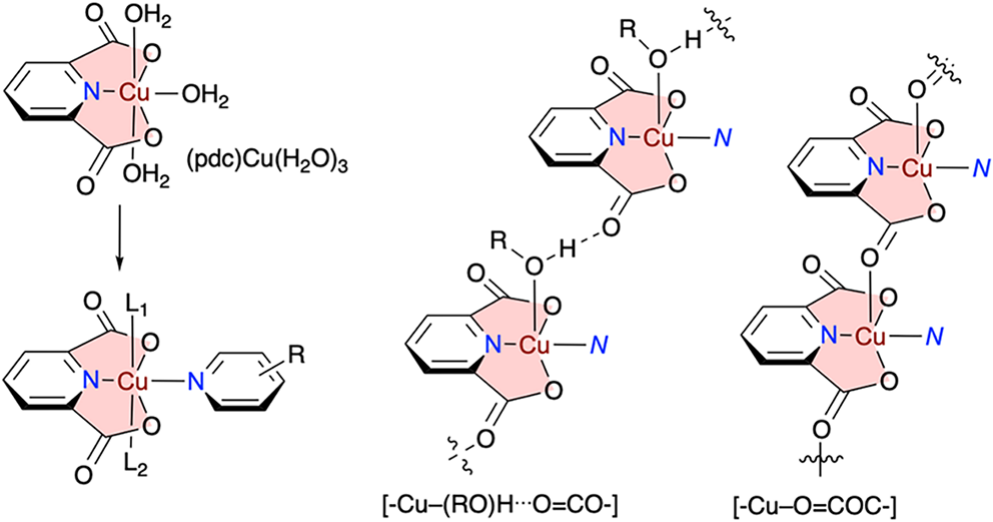
Left: Formation of the square-planar complex Cu­(O_2_N_2_). Right: Two modes of association of neighboring (pdc)­Cu units through the H-bonding of a ROH fragment (typically water) or direct bonding of C=O to Cu.

Recent developments in Blatter radical chemistry have opened up access to planar analogues of **1a**, such as **2a** ^58^ and its functional derivatives,^59^ that have more favorable packing properties and spin delocalization. The radical **2a** was initially obtained in low yields by organolithium methods^58^ and later by aza-Pschorr,^60^ photochemical,^61^ and, the most effective, TMS_3_SiH-assisted cyclization^62^ (up to 96% yield) methods. These methods provide convenient access not only to **2a** and its functional derivatives, but also enable a formal “docking” of the spin-bearing [1,2,4]­triazinyl fragment to larger polycyclic aromatic systems.^63–67^ Such derivatives with strategically placed metal ion binding sites and paramagnetic ions with a proper coordination environment, such as (pdc)­Cu, may provide an attractive avenue to materials with higher dimensionalities of magnetic interactions.

Herein, we report two planar Blatter radicals **2c** and **2d** containing the pyridyl substituent at the C(2) position as monodentate ligands. The synthesis and characterization of the ligands and formation of complexes **2c­[Cu]** and **2d­[Cu]** (Figure 3) with the (pdc)Cu coordination building block are described. One of the complexes is characterized structurally and investigated by superconducting quantum interference device (SQUID) magnetometry. Analysis of experimental data is augmented with density functional theory (DFT) computational results.

**Figure 3:**
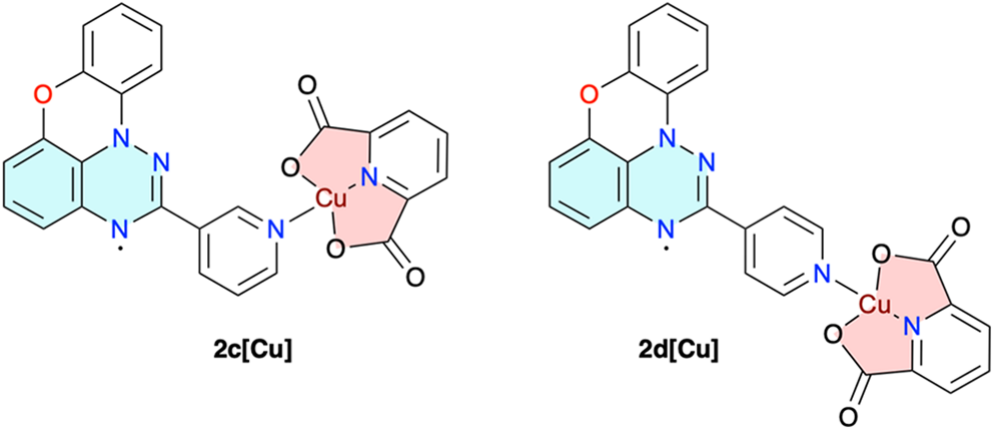
Structures of complexes **2c­[Cu]** and **2d­[Cu]**.

## Results and Discussion

### Synthesis

Radicals **2c** and **2d** were obtained using the procedure developed for the preparation of the parent radical **2a**,^62^ and involving the TMS_3_SiH-assisted cyclization of appropriate iodides **3**, as shown in Scheme 1. The original procedure was modified in response to the solubility and reactivity of the intermediates affected by the presence of the pyridyl group. Thus, commercial hydrazides **4c** and **4d** were reacted with 1,2-difluoro-3-nitrobenzene in dimethyl sulfoxide (DMSO), giving hydrazides **5c** and **5d** in 85 and 66% yield, respectively (recrystallized from aq MeOH). Attempted reductive cyclization of the resulting hydrazides **5** to triazines **6** using the typical Sn/AcOH conditions failed, and only complex mixtures of products were obtained. It was speculated that metallic tin reduces the protonated pyridyl group in **5**, or any intermediate to **6**, leading to byproducts. Therefore, Sn/AcOH was replaced with the SnCl_2_·H_2_O/EtOH system, leading to purple and dark red solutions of the *leuco* forms of **6c** and **6d**, respectively.

**Scheme 1:**
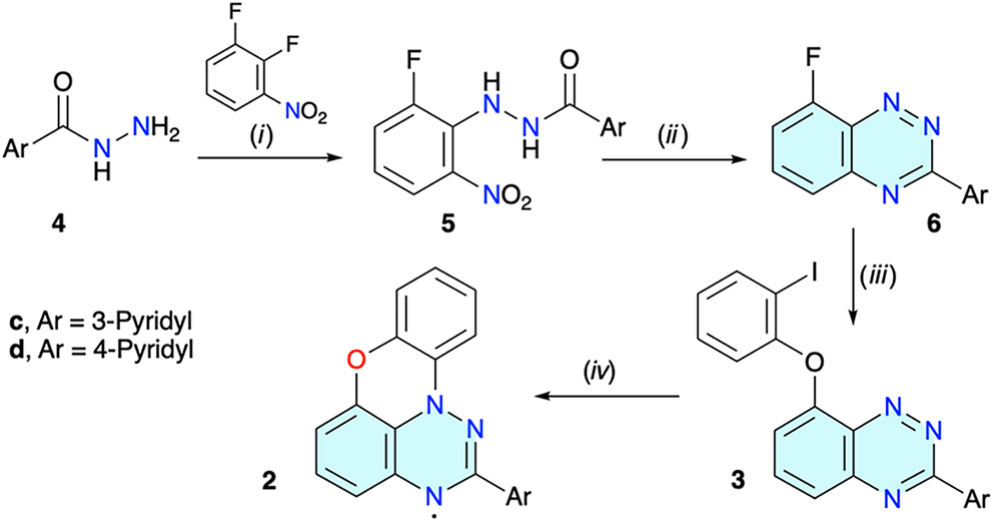
Synthesis of the Radical **2** ^a^

a Reagents and conditions: (i) DMSO, 100 °C, 14 h, 85% **5c** and 66% **5d**; (ii) (1) SnCl_2_·2H_2_O, EtOH, rflx, 12 h; (2) air 45% **6c** and 74% **6d**; (iii) NaH, dry DMSO, 80 °C, 12 h, ∼70%; (iv) TTMSS, AIBN, toluene/pyridine, 80 °C, 4 h; (2) air ∼50%.

Exposure of the reaction mixtures to air gave triazines **6c** and **6d** as bright yellow solids in 45 and 74% yield, respectively. The lower yield of former triazine **6c** is presumably due to its lower chemical stability during the workup and purification steps.

Triazines **6** were reacted with 2-iodophenol in DMSO in the presence of NaH, giving the iodoarenes **3** in about 70% yield after purification. Interestingly, small amounts of dark-green side products that matched the properties of the radical **2** were observed during column chromatography of iodides **3**, presumably formed in light-induced cyclization.^61^

Finally, the cyclization of the aryl iodide **3** to the radical **2** was accomplished under radical chain reaction conditions in the presence of TMS_3_SiH (TTMSS). Reactions of **3c** and **3d** conducted in anhydrous toluene were very slow and inefficient. Addition of pyridine to the reaction mixture as a silyl iodide scavenger proved to be effective. Thus, cyclization of each **3c** and **3d** in a toluene/pyridine mixture (6:1), followed by aerial oxidation of the resulting leuco forms and column chromatography, gave the desired radicals **2c** and **2d**, respectively, in about 50% yield as dark-green solids.

Copper complexes **2c­[Cu]** and **2d­[Cu]** (Figure 3) were prepared in 90 and 75% yields, respectively, by slow evaporation of dilute solutions of radicals **2c** and **2d** with (pdc)­Cu­(H_2_O)_3_ ^68,69^ in a MeOH/EtOH mixture (1:9).

### Molecular and Crystal Structures

The complex **2c­[Cu]** crystallizes (MeOH/EtOH, 1:9) in the triclinic space group *P*1̅. Repeated attempts at slow evaporation of the MeOH/EtOH solution of **2d­[Cu]** gave only powders that were unsuitable for XRD analysis. Similarly, no suitable crystals of either radical **2c** or **2d** could be obtained through crystallization using a full polarity range of solvents and mixtures. In contrast, XRD structures of several intermediates, iodide **3c** (triclinic, *P*1̅), fluoride **6c**, and hydrazide **5d** (both monoclinic, *P*2_1_/*n*), were obtained, all containing a single molecule in the asymmetric unit. The results for **2c­[Cu]** are shown in Figures 4–6, and full data for all structures are provided in the Supporting Information (SI).

**Figure 4:**
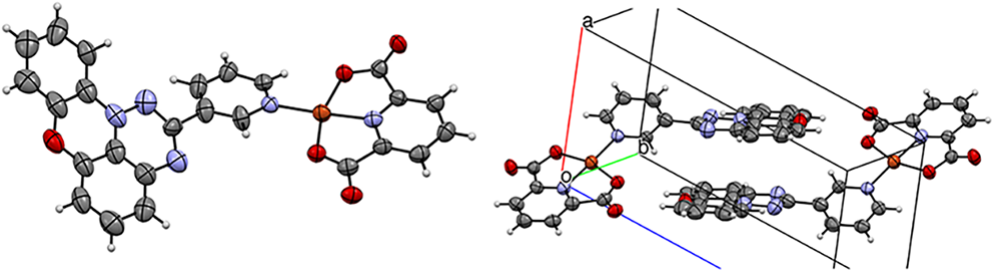
Atomic displacement ellipsoid representations of the individual molecule (left) and unit cell (right) of **2c­[Cu]**. The ellipsoids are drawn at the 50% probability level. Color codes: C, gray; O, red; N, blue; and Cu, brown.

Analysis of the molecular structure of **2c­[Cu]** revealed the planar (pdc)Cu fragment, with the pyridyl group of the radical **2c** completing the square-planar coordination environment of the Cu ion (Figure 4). The geometry of the (pdc)Cu moiety is typical, with a N–Cu distance of 1.913(6) Å and an O–Cu–O angle of 161.3(2)°. The paramagnetic pyridyl ligand **2c** is connected to (pdc)­Cu with a N–Cu bond length of 1.958(5) Å, and is twisted relative to the (pdc)Cu plane by 12.7°, which again is typical for other (pdc)­Cu–pyridine complexes.^70–72^ The planar Blatter radical fragment has usual dimensions,^59^ and it forms a torsion angle of 39.0° with the pyridine ring. Overall, the mean planes of (pdc)Cu and triazino­[5,6,1-*kl*]­phenoxazine form an angle of 34.4°.

The unit cell of **2c­[Cu]** contains two molecules in which the planar triazino­[5,6,1-*kl*]­phenoxazine fragments form a discrete antiparallel dimer (related by an inversion center) with a distance of 3.363 Å between their mean planes (Figure 4).

The molecules are arranged in the crystal in such a way that the carbonyl groups of neighboring molecules coordinate to the Cu center, providing an axial orientation of the ligands and thus completing the octahedral coordination sphere (Figure 5). This leads to an infinite ladder-type (Figure 6) backbone formed by two edge-connected 8-membered rings with alternating Cu–O distances (2.755(5) and 2.570(5) Å) as the repeating unit (Figure 5). To the best of our knowledge, this represents a new structural motif in the coordination chemistry of the (pdc)­Cu building block.

**Figure 5:**
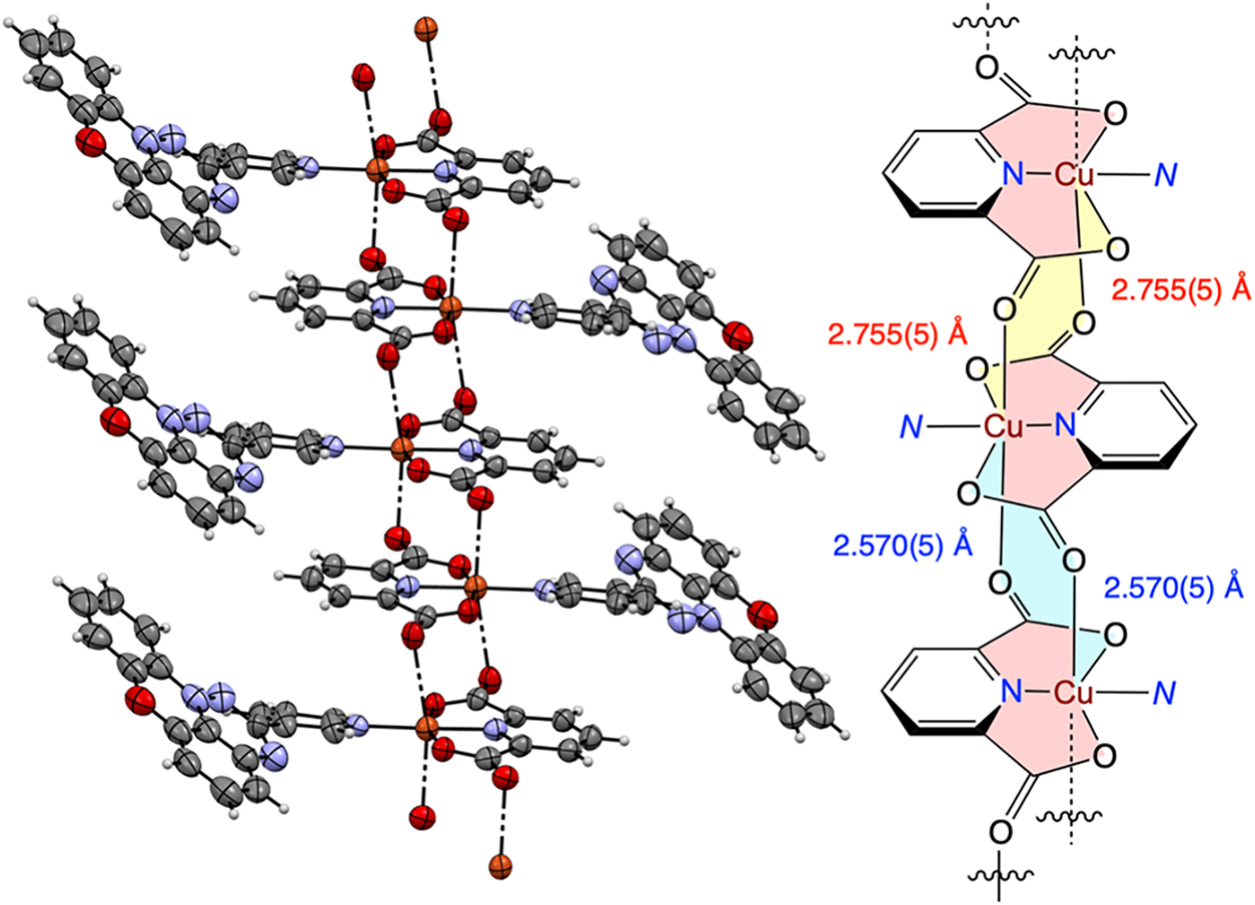
Left: Partial packing diagram for **2c­[Cu]**. Right: Schematic representation of the main chain of the coordination polymer composed of fused “yellow” and “blue” 8-membered rings with indicated different O–Cu distances. N represents the ligand **2c**. Other views are shown in the SI.

**Figure 6:**
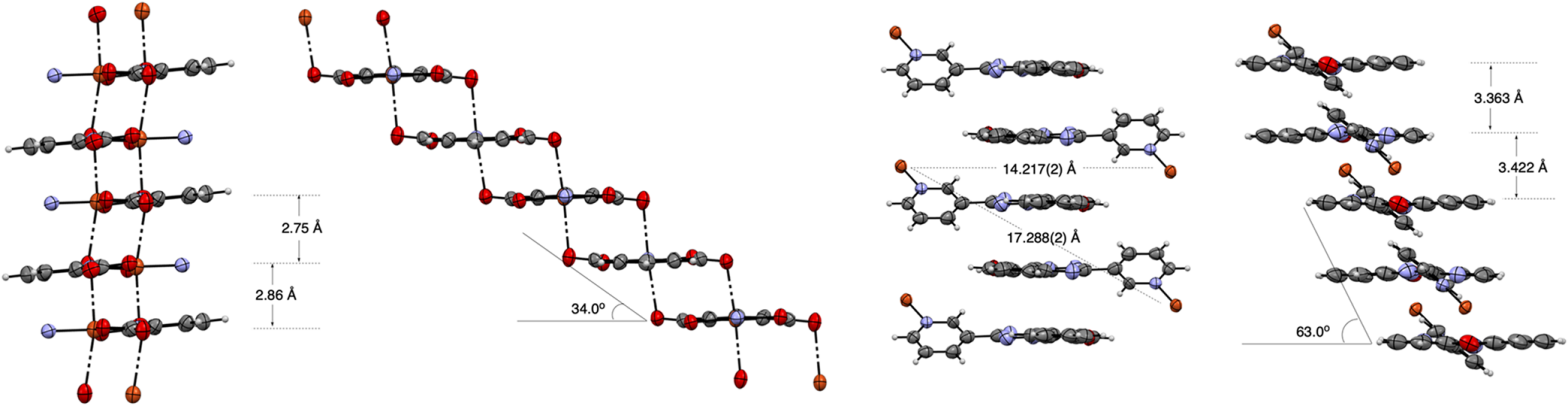
Left: Two views of the **2c­[Cu]** coordination polymer backbone. The radical ligand **2c** is abbreviated as the N atom (blue). Right: Two views of the radical fragment with the coordinated Cu atom (brown). The distances are between the mean planes of the (pdc)Cu fragments (left) and triazino­[5,6,1-*kl*]­phenoxazine rings (right). The slippage angle is shown in degrees. Color codes: C, gray; O, red; N, blue; and Cu, brown.

Unlike the previously reported oligomers and polymers, each (pdc)­Cu fragment is engaged in a total of four C=O···Cu contacts, resulting in two mutual C=O···Cu interactions between any two neighboring (pdc)Cu units (Figures 5 and 6). The observed distances are consistent with those reported for hexacoordinated Cu centers (axial C=O and H_2_O ligands),^51,55,56^ while shorter C=O···Cu distances, ∼2.4 Å, were reported for complexes with pentacoordinated Cu centers.^54,57^

The planar Blatter ligands **2c** are positioned on two sides of the polymeric backbone, and the individual strands are arranged parallel to each other and separated by 17.058(2) Å. This leads to interdigitation and consequently the formation of slipped stacks of discrete π–π dimers with alternating mean interplanar distances of 3.363 and 3.422 Å and a slippage angle of 27° (Figure 6).

### Characterization of Radicals

To assess the effect of the pyridine ring at the C(2) position on electronic properties, radicals **2c** and **2d** were analyzed by spectroscopic (UV–vis and EPR) and electrochemical methods, and results were compared to those of the parent radical **2a**.

Both pyridyl radicals exhibit five low-intensity absorption bands in the visible range, tailing to about 750 nm, which are nearly identical to those observed in parent **2a** (Figure 7). The lowest-energy maximum in **2a** (λ_max_ = 675 nm) is slightly bathochromically shifted in pyridine derivatives **2c** and **2d** by 5.5 and 3 nm, respectively. This trend is reproduced by time-dependent-DFT (TD-DFT) calculations (Table 1).

**Figure 7:**
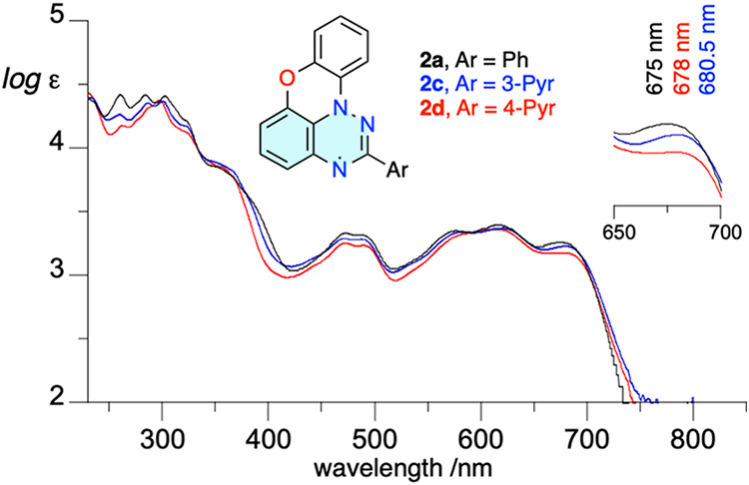
UV–vis spectra for radicals **2a** (black), **2c** (blue), and **2d** (red) recorded in CH_2_Cl_2_. The inset shows the expanded low-energy part of the plot.

**Table 1:** Selected Experimental and DFT Electronic Parameters for the Radicals **2**

radical	R	λ_max,exp_ ^a^/nm	λ_max,theor_ ^b^/nm	*E*_1/2_^–1/0 ^ ^c^/V	*E*_1/2_^0/+1^ ^c^/V	*E*_cell_ ^d^/V	*E*_α‑HOMO_ ^b^/eV	*E*_β‑LUMO_ ^b^/eV	*a*_N(12)_ ^e^/G	*a*_N(1)_ ^e^/G	*a*_N(3)_ ^e^/G
**2a** ^f^	Ph	675	576	–1.306	–0.153	1.153	–4.835	–2.938	7.43	4.21	4.47
**2c**	3-pyridyl	680.5	578	–1.275	–0.096	1.179	–4.904	–3.009	7.54	4.07	4.42
**2d**	4-pyridyl	678	577	–1.255	–0.074	1.181	–4.939	–3.048	7.53	3.99	4.51

a The lowest-energy absorption band recorded in CH_2_Cl_2_.

b D_0_ → D_2_ excitation with β-HOMO → β-LUMO character obtained at the TD UB3LYP/6-311G­(d,p)//UB3LYP/6-311G­(d,p) level of theory in CH_2_Cl_2_ dielectric medium.

c Potentials vs Fc/Fc^+^ couple (0.46 V vs saturated calomel electrode (SCE)).^73^ Recorded in CH_2_Cl_2_ with Bu_4_N^+^PF_6_^–^ (100 mM), at ca. 20 °C, 50 mV s^–1^, glassy carbon working electrode.

d *E*_cell_ = *E*_1/2_^0/+1^ – *E*_1/2_^–1/0^.

e Recorded in benzene at ca. 20 °C.

f Ref 74.

TD-DFT calculations demonstrated that the observed lowest-energy absorption band in **2** can be ascribed to D_0_ → D_2_ excitation with β-HOMO → β-LUMO character (∼96%) delocalized in the triazino­[5,6,1-*kl*]­phenoxazine. In contrast, the D_0_ → D_1_ excitation consists mainly of the α-HOMO → α-LUMO transition (∼96%) and has some intramolecular charge transfer (CT) character involving the excitation from triazino­[5,6,1-*kl*]­phenoxazine to benzo­[*e*]­[1,2,4]­triazine-pyridine. It has an order of magnitude lower oscillator strength, *f* (∼0.003), and most likely is not easily observed experimentally.

Cyclic voltammetry revealed that in analogy to parent **2a**, the oxidation and reduction processes are essentially reversible for radicals **2c** and **2d** (Figure 8). Potentials for both processes are shifted anodically relative to that of the planar Blatter **2a** (Table 1), and the shift correlates well with Hammett parameters^75^ σ_p_ for substituents at the C(2) position (Ph, 3-pyridyl, and 4-pyridyl, Figure 8). Analysis of the plots indicates that the C(2) substituent has nearly 60% greater impact on the highest-occupied molecular orbital (HOMO) (slope ρ_ox_ = 0.18(3)) than on the lowest-unoccupied molecular orbital (LUMO) (ρ_red_ = 0.114(4), Figure 8). As a consequence, the electrochemical window in series 2 widens for substituents with increasingly stronger electron-withdrawing properties (see Table 1).

**Figure 8:**
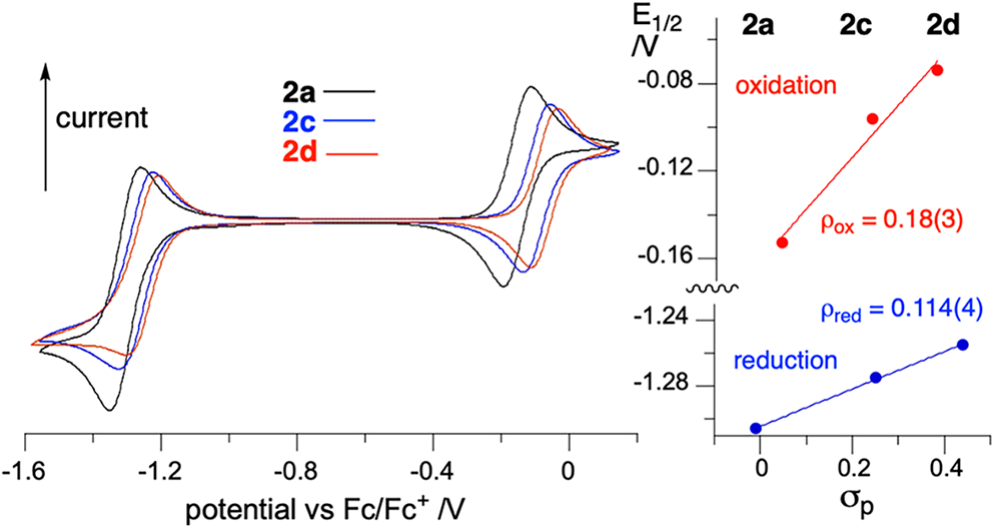
Left: Cyclic voltammograms for the radicals **2**; 1 mM in the CH_2_Cl_2_/Bu_4_N^+^PF_6_^–^ electrolyte (100 mM), at ca. 20 °C, 50 mV s^–1^, glassy carbon working electrode. Right: Correlation of *E*_1/2_^–1/0^ (blue) and *E*_1/2_^0/+1^ (red) vs the Hammett substituent parameters σ_p_. See the SI for details.

This result is different from that obtained for a series of C(10) derivatives of the planar Blatter **2a**,^59^ in which the C(10) substituents have a slightly stronger impact on the LUMO than on the HOMO (ρ_red_ = 0.19(2) and ρ_ox_ = 0.163(14), respectively). On the other hand, the results for series **2** are similar to those observed in a family of C(3) derivatives of the parent Blatter radical **1a**, although the effect is nearly twice as stronger in the C(3) derivatives with slopes ρ_ox_ = 0.33(5) and ρ_red_ = 0.19(5).^74^

### EPR Spectroscopy

The EPR spectra of radicals **2c** and **2d** measured at ambient temperature in benzene solutions exhibit multiplet signals typical for planar Blatter radicals.^59,60^ Simulation of the spectra performed using three ^14^N and four ^1^H nuclei (Figure 9 and SI) gave hyperfine coupling constants (hfcc), assigned to specific nuclei on the basis of DFT results: *a*_N(12)_ ≈ 7.50 G, *a*_N(1)_ ≈ 4.0 G, and *a*_N(3)_ ≈ 4.4 G (Table 1). In addition, two large *a*_H_ ≈ 1.8 G constants were found, which are consistent with substantial positive spin densities of about 0.09 calculated at the C(9) and C(11) positions.

**Figure 9:**
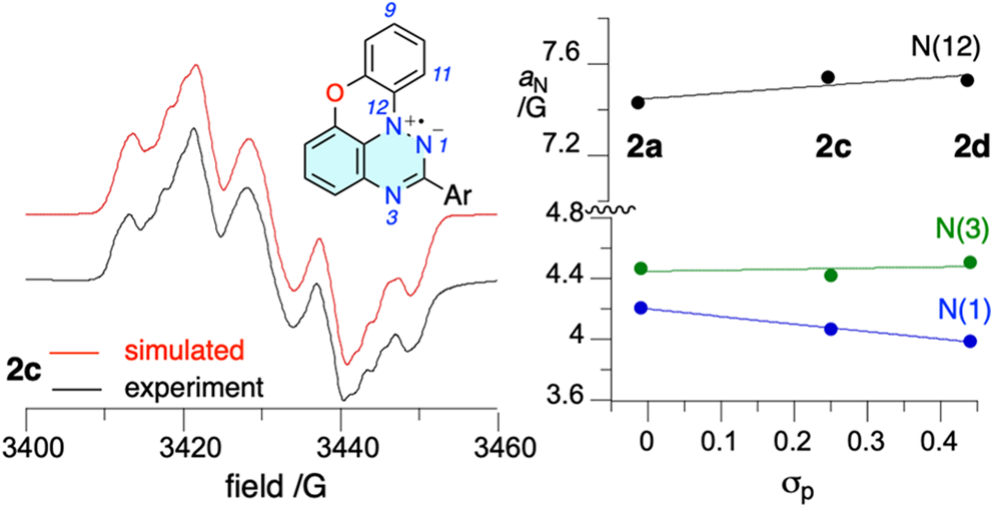
Left: Experimental (black) and simulated (red) EPR spectra for **2c** recorded in benzene at ca. 20 °C. *g* = 2.0055. Right: Correlation of *a*_N_ hfcc for radicals **2** with the C(2) substituent Hammett parameters σ_p_. For details, see the SI.

Correlation analysis of the *a*_N_ values for pyridyl derivatives **2c** and **2d** and the parent **2a** revealed that electron-withdrawing substituents at the C(2) position of **2** shift spin density from the N(1) to the N(12) position, while spin concentration on N(3) remains nearly unchanged. This is consistent with the increasing contribution of the zwitterionic resonance form stabilized by the electron-withdrawing groups (e.g., pyridyl), and, consequently, greater spin delocalization, as evident from the radical delocalization value^76^ (RDV):^77^ the RDV^–1^ is 3.39 for **2a** and 3.41 for **2c** and **2d**. A comparison of the experimental and theoretical hfcc values demonstrates that DFT underestimated the *a*_N_ constants by 1.52(1) G for N(12), 0.42(1) G for N(1), and 0.31(1) G for N(3) and overestimated the largest *a*_H_ values by about 0.6 G.^77^

Copper­(II) complexes **2c­[Cu]** and **2d­[Cu]** in the solid-state exhibit pseudoaxial EPR spectra with *g*_*x*_ ≈ 2.03, *g*_*y*_ ≈ 2.04, *g*_*z*_ ≈ 2.15, and *g*_iso_ ≈ 2.07 (Figure 10). The observed pattern of *g*-values, *g*_*z*_ > *g*_*x*_ ∼ *g*_*y*_ > 2.0, is typical of an axially elongated octahedral coordination geometry^78^ and consistent with the XRD structure of **2c­[Cu]** (*vide supra*). Simulation of both powder EPR spectra gives very little residue,^77^ hence there is no clear evidence for the contribution of the Blatter radical ligand to the EPR spectra of these complexes. This is consistent with an arrangement in which the paramagnetic ligands are strongly coupled antiferromagnetically and therefore are EPR silent, as observed in **2c­[Cu]** (*vide infra*).

**Figure 10:**
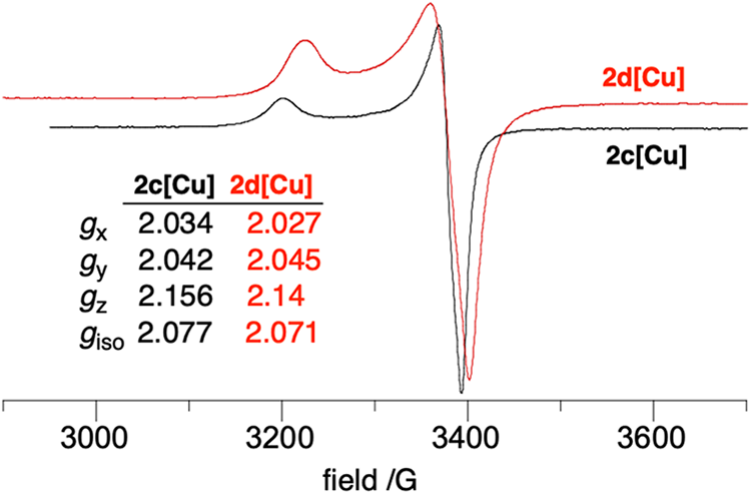
Powder EPR spectra for **2c­[Cu]** (black) and **2d­[Cu]** (red) complexes recorded at ca. 20 °C.

### DFT Studies

Magnetic interactions within the monomeric complex and between neighboring paramagnetic fragments in the crystal structure of **2c­[Cu]** were investigated by DFT methods. Thus, exchange interactions between the Cu­(II) center and the radical in the monomeric unit, between the nearest radical ligands **2c**, and between copper centers in the Cu···O···Cu bridge at their crystallographic coordinates were calculated using the broken symmetry (BS) approach and the Yamaguchi formalism^79,80^ (eq 1), where *E* is the self-consistent field (SCF) energy and ⟨*S* ^2^⟩ is the total spin angular momentum of the high (*T*) or low (OSS) spin state.

1 $$\Delta E_{S-T}=J=2\frac{E_{\mathrm{OSS}}-E_{T}}{{\left\langle S^{2}\right\rangle }_{T}-{\left\langle S^{2}\right\rangle }_{\mathrm{OSS}}}$$

Results obtained at the UB3LYP/6-31++G­(d,p) level of theory demonstrate that the spins of Cu­(II) and the radical ligand are weakly coupled ferromagnetically with an exchange interaction energy of *J*_Cu···R_ = 3.6 cm^–1^ with H_2_O ligands and *J*_Cu···R_ = 3.7 cm^–1^ without the ligands. This weak exchange interaction is consistent with the separation of the two magnetic moments and spin densities with the pyridine fragment (Figure 11). Exchange interactions between the Cu centers in the isolated Cu···O···Cu bridge are calculated at *J*_Cu···Cu_ = −3.1 cm^–1^ for both geometries. For details, see the SI.

**Figure 11:**
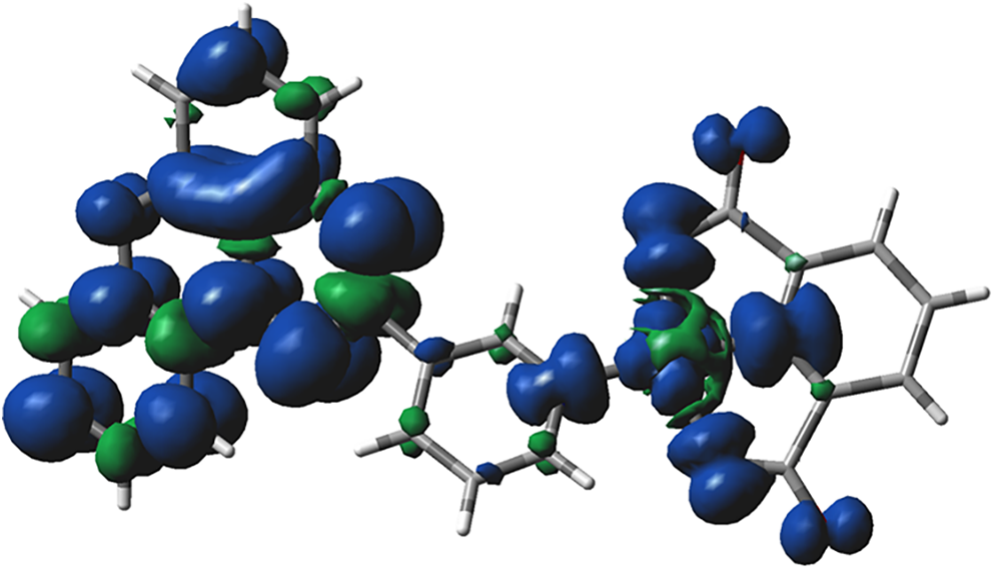
Spin distribution in the triplet state of the monomeric unit of **2c­[Cu]** without H_2_O axial ligands calculated (UB3LYP/6-31++G­(d,p)) at the crystallographic coordinates.

The same calculations for neighboring ligands revealed that both pairs exhibit antiferromagnetic exchange interactions with *J*_1R···R_ = −356 cm^–1^ for the tighter discrete pair in the unit cell (Figure 6) with 3.363 Å of π–π separation and *J*_2R···R_ = −143 cm^–1^ for the second pair with 3.422 Å separation of the mean planes. The magnitude of these exchange interactions indicates a negligible contribution of the ligands to the paramagnetic signal of the solid sample, which is in agreement with the solid-state EPR spectra (*vide supra*).

### SQUID Magnetometry

The magnetic susceptibility of a polycrystalline sample of the complex **2c­[Cu]** was measured on a Quantum Design SQUID magnetometer (MPMS-XL-7T) at 300 → 2 K in field swept mode using a sweep rate of 1 K min^–1^ and an applied field of 0.6 T. The total magnetic susceptibility of **2c­[Cu]** was corrected for sample diamagnetism, and the resultant paramagnetic susceptibility, χ_p_, was analyzed as the χ_p_ *T* product. Thus, the temperature dependence of χ_p_ *T* shown in Figure 12 reveals a gradual decline in χ_p_ *T* upon cooling from 0.428 emu K mol^–1^ at room temperature to 0.377 emu K mol^–1^ at ca. 50 K and then a more rapid decrease down to 0.137 emu K mol^–1^ at the base temperature of 2 K.

**Figure 12:**
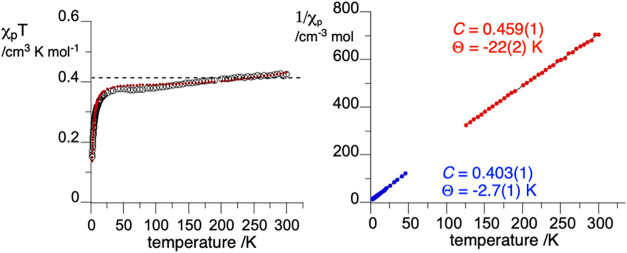
Left: Temperature dependence of χ_p_ *T* for **2c­[Cu]**. The red dashed line corresponds to the double alternating chain model (eqs 3 and 4). The horizontal line marks the Curie constant for an isolated Cu­(II) ion (*C* = 0.405 cm^3^ K mol^–1^ based on the experimental *g*_iso_). Right: The Curie–Weiss analysis of 1/χ_p_ data for **2c­[Cu]** in two temperature regimes. For details, see the text and SI.

Analysis of the 1/χ_p_(*T*) curve indicates two linear regions (Figure 12), which were modeled separately using the Curie–Weiss model (eq 2). Thus, in the high-temperature regime (*T* > 100 K), **2c­[Cu]** follows Curie–Weiss behavior with *C* = 0.459(1) cm^3^ K mol^–1^ and θ = −22(2) K (Figure 12). This Curie constant reflects contributions arising from both the Cu­(II) ion and the radical ligand, but the value of *C* is significantly lower than that expected for two independent *S* = 1/2 ions (*C*_Cu_ + *C*_R_ = 0.405 + 0.375 = 0.780 emu K mol^–1^, assuming *g*_iso_ = 2.077 for Cu­(II) and *g* = 2.000 for **2c)**. The large Weiss constant is indicative of strong antiferromagnetic interactions in this regime. Conversely, in the low-temperature regime (*T* < 50 K), the sample again follows Curie–Weiss behavior but with *C* = 0.403(1) cm^3^ K mol^–1^ and θ = −2.7(1) K, consistent with a magnetic contribution from only the Cu­(II) ion, while the paramagnetism of the pendant radical ligands is largely quenched due to strong antiferromagnetic exchange interactions.

2 $$\chi_{\mathrm{tot}}=\frac{C}{T-\theta }$$

The results of the Curie–Weiss analysis are supported by the XRD and DFT data (*vide supra*), suggesting that the magnetism of **2c­[Cu]** is comprised of two essentially independent contributions: (i) an alternating one-dimensional chain of Cu­(II) ions bridged by pairs of carboxylate groups, with small antiferromagnetic exchange, and (ii) an alternating one-dimensional chain of radicals with π–π interactions favoring strong antiferromagnetic exchange (|*J*_RR_| ∼ 102 cm^–1^ by DFT). Therefore, the magnetic behavior of **2c­[Cu]** was modeled as two independent alternating one-dimensional (1D) Heisenberg chains, one corresponding to strong radical–radical exchange, *J*_RR_, and one with weaker Cu···Cu exchange, *J*_CuCu_. The spin Hamiltonian for such a system can be written as eq 3, in which α_*x*_ is an alternation parameter assuming values in a range of α_*x*_ = 1, for a regular chain, and α_*x*_ = 0, for isolated pairs of spins.

3 $$\begin{array}{l} Ĥ=-J_{\mathrm{CuCu}}\left[{Ŝ}_{\mathrm{Cu}1}{Ŝ}_{\mathrm{Cu}2}+a_{\mathrm{CuCu}}{Ŝ}_{\mathrm{Cu}2}{Ŝ}_{\mathrm{Cu}3}\cdot \cdot \cdot \right]\\ -J_{\mathrm{RR}}\left[{Ŝ}_{R1}{Ŝ}_{R2}+a_{\mathrm{RR}}{Ŝ}_{R2}{Ŝ}_{R3}\cdot \cdot \cdot \right] \end{array}$$

Consequently, the magnetic data for **2c­[Cu]** were modeled as the sum of two independent alternating chains, each described by the Hatfield model^81^ (eq 4). This model is based on a cluster approach of ten interacting spins, with one set of parameters *A*–*F* for 0 ≤ α ≤ 0.4 (nearer the dimer pair limit) and another set for 0.4 ≤ α ≤ 1.0 (near the regular chain limit).^77,82^

4 $$\chi_{p}\left(T\right)=\frac{N_{A}g^{2}\mu_{B}^{2}}{k_{B}T}\frac{A+\mathrm{Bx}+Cx^{2}}{1+\mathrm{Dx}+Ex^{2}+Fx^{3}}$$

$$x=\left|J\right|/k_{B}T$$

To reduce the number of parameters, the *g*-values were fixed at 2.077 for Cu and 2.00 for the radical, based on the EPR studies. Fitting of the χ_p_ *T* data was commenced using the initial magnitude of the exchange coupling *J*_RR_ and the alternation parameter α, as determined by DFT. For the low-temperature data, the initial magnitude of *J*_CuCu_ was estimated from the mean field model (eq 5):

5 θ=2zJS(S+1)/3k

where θ is the Weiss constant and *z* is the number of nearest exchange-coupled neighbors (*z* = 2 for a linear chain). Based on the low-temperature data (Figure 12), θ = −2.7 K gave *J*_CuCu_/*k*_B_ ∼ −2.7 K. Minimization of the *R*-function (eq 6) afforded *J*_RR_/*k*_B_ = −1200 K and α_RR_ = 0.4 for the radical chain, and *J*_CuCu_/*k*_B_ = −3.5 K with α_CuCu_ = 0.9 (*R* = 0.0088).

6 $$R=\frac{1}{n}\sum_{i=1}^{n}\sum \left|\chi T_{\mathrm{obs}}-\chi T_{\mathrm{calc}}\right|$$

## Summary and Conclusions

Two planar monodentate paramagnetic ligands based on the planar Blatter radical **2a** were obtained. Electrochemical and EPR analyses demonstrated a significant effect of the electron-withdrawing pyridyl groups on redox potentials and spin distribution in the radicals, which correlates with the Hammett parameters of the substituents.

The pyridyl radicals formed complexes with the (pdc)Cu building block. Structural analysis of the complex **2c­[Cu]** revealed a new Cu···O=C··· ladder-type linear coordination polymer, which provides a framework for a π-stacking arrangement of the paramagnetic ligands. DFT and magnetic data for **2c­[Cu]** indicate that both the Cu­(II) ions and the ligands **2c** can be treated as isolated 1D alternating antiferromagnetic chains, which can be analyzed using the Hatfield model.

The presented results demonstrate a new structural motif in coordination chemistry in which the paramagnetic Cu­(II) ions form a backbone, which, in turn, imposes stacking of planar radical ligands. It is possible that the formation of this structure results from synergistic forces: completing the octahedral coordination sphere of the Cu­(II) ion and π–π stacking with spin pairing of the radical ligands.

These findings open the possibility to manipulate the supramolecular structure of such complexes by using other tridentate pdc-type ligands, such as dimeric pdc,^83^ and planar Blatter radicals with differently substituted pyridine (monotopic ligands) or two pyridine functionalities substituted at the triazino­[5,6,1-*kl*]­phenoxazine core (ditopic ligands) to achieve other topologies of paramagnetic N ligands.^84,85^

## Computational Details

Quantum-mechanical calculations were carried out using the Gaussian 16 suite of programs.^86^ Geometry optimizations of radical **2** were performed at the UB3LYP/6-311G­(d,p) level of theory in a vacuum, using tight convergence limits and the Cs symmetry constraints. Isotropic Fermi contact coupling constants (hfcc) and spin densities for radicals were calculated using the UCAM-B3LYP/EPR-II//UB3LYP/6-311G­(d,p) method in benzene dielectric medium implemented with the polarizable continuum model (PCM).^87^ Electronic excitation energies in CH_2_Cl_2_ dielectric medium were obtained at the UB3LYP/6-UB3LYP/6-311G­(d,p)//UB3LYP/6-311G­(d,p) level of theory using the TD-DFT method.^88^ Exchange interaction energies *J*_RR_(DFT) between the nearest radical ligands were obtained using single-point calculations at the UB3LYP/6-31++G­(d,p) level of theory for two pairs of ligands at their crystallographic coordinates and the Yamaguchi formalism^79,80^ (eq 1).

## Experimental Section

All of the reagents and solvents were obtained commercially and used as received. Anhydrous tetrahydrofuran (THF) and CH_2_Cl_2_ were obtained from the MBRAUN solvent purification system. All chemical operations were performed without contact with metal objects or salts. Reactions were carried out under an inert atmosphere (N_2_ or Ar gas), while subsequent workup of reactions and isolation of the product were conducted in air. Oil baths were used for heating reactions. All volatiles were removed under reduced pressure. All reaction mixtures and column eluents were monitored by TLC using commercial aluminum-backed thin-layer chromatography (TLC) plates (Merck Kieselgel 60 F_254_ or Merck Al_2_O_3_ F_254_ neutral). The plates were observed under UV light at 254 and 365 nm. Chromatographic purifications were performed by using silica gel 60 (70–230 mm, Merck). Melting points were determined on a MEL-TEMP apparatus and are uncorrected. ^1^H and ^13^C NMR spectra were obtained at 400 and 101 MHz, respectively, on a Bruker AV 400 Neo spectrometer in CDCl_3_ and referenced to the solvent (δ = 7.26 ppm for ^1^H and δ = 77.16 ppm for ^13^C) or in DMSO-*d*_6_ and referenced to the solvent (δ = 2.50 ppm for ^1^H and δ = 39.52 ppm for ^13^C).^89^ High-resolution mass spectrometry (HRMS) measurements were performed using a SYNAPT G2-Si High-Definition Mass Spectrometry (Waters) equipped with an eletrospray ionization (ESI) or atmospheric pressure chemical ionization (APCI) source and Quantitative Time-of-Flight (QuanTof) mass analyzer. Passivated SiO_2_ was prepared by suspension in 2% solution of Et_3_N in CH_2_Cl_2_ and then evaporation to dryness.

### Preparation of Radicals **2c** and **2d**. General Procedure

A solution of tris­(trimethylsilyl)­silane (TTMSS, 497.3 mg, 2.0 mmol) and iodide **3c** or **3d** (426.0 mg, 1.0 mmol) in a mixture of dry pyridine (2 mL) and dry toluene (12 mL) was stirred at 80 °C under an Ar atmosphere. A solution of azobisisobutyronitrile (AIBN) (98.5 mg, 0.6 mmol) in dry toluene (2 mL) was dispensed to the reaction flask over the course of 4 h using a syringe pump. Once all AIBN solution was added, the reaction flask was removed from heat, the septum was removed, and the solution was allowed to stir open to air overnight. The solvent of the resulting green solution was evaporated completely to give a green solid. The solid residue was absorbed onto passivated SiO_2_ and separated by column chromatography (passivated SiO_2_, CH_2_Cl_2_/AcOEt, 5:1), giving **2c** or **2d** as a dark-green solid.

#### 2-(Pyridin-3-yl)-3*H*-[1,2,4]­triazino­[5,6,1-*kl*]­phenoxazin-3-yl (**2c**)

Following the general procedure, **2c** (101 mg, 56% yield) was obtained starting from 256 mg (0.60 mmol) of **3c**, 0.38 mL of TTMSS (1.20 mmol), and 59 mg (0.36 mmol) of AIBN. Mp 230–232 °C (CH_2_Cl_2_/AcOEt); IR (neat) ν 1575, 1459, 1386, 1345, 1276, 1126, 814, 740, 704 cm^–1^; UV–vis (CH_2_Cl_2_), λ_max_ (log ε) 680.5 (3.23), 621 (3.37), 583 (3.34), 488 (3.28), 471 (3.29), 300.5 (4.37), 286 (4.35), 260 (4.26); ESI­(+)-MS *m*/*z* 300 (100, [M + H]^+^); HRMS (ESI-TOF) *m*/*z* M^+^ calcd for C_18_H_11_N_4_O: 299.0933, found 299.0937. Anal. Calcd for C_18_H_11_N_4_O: C, 72.23; H, 3.70; N, 18.72. Found: C, 72.19; H, 3.82; N, 18.63.

#### 2-(Pyridin-4-yl)-3*H*-[1,2,4]­triazino­[5,6,1-*kl*]­phenoxazin-3-yl (**2d**)

Following the general procedure, **2d** (110 mg, 47% yield) was obtained starting from 332 mg (0.78 mmol) of **3d**, 0.5 mL of TTMSS (1.56 mmol), and 77 mg (0.47 mmol) of AIBN. Mp 215–218 °C (CH_2_Cl_2_/AcOEt); IR (neat) ν 1600, 1574, 1483, 1459, 1387, 1279, 1079, 836, 784, 754, 683 cm^–1^; UV–vis (CH_2_Cl_2_), λ_max_ (log ε) 678 (3.17), 613 (3.36), 489.5 (3.23), 472 (3.26), 297.5 (4.37), 262 (4.175); ESI­(+)-MS *m*/*z* 300 (100, [M + H]^+^); HRMS (ESI-TOF) *m*/*z* M^+^ calcd for C_18_H_11_N_4_O: 299.0933, found 299.0935. Anal. Calcd for C_18_H_11_N_4_O: C, 72.23; H, 3.70; N, 18.72. Found: C, 72.21; H, 3.83; N, 18.70.

### Preparation of Complexes **2c­[Cu]** and **2d­[Cu]**. General Procedure

The radical **2c** or **2d** (30 mg, 0.10 mmol) was dissolved in a warm mixture of EtOH and MeOH (9:1, 15 mL) in a conical flask to give a dark blue solution. The complex Cu­(pdc)­(H_2_O)_3_ ^51^ (28 mg, 0.10 mmol) was dissolved in the same solvent mixture (3 mL) to give a pale blue solution. The two solutions were mixed and left to slowly evaporate for 2 days. The resulting dark blue needles of **2c­[Cu]** and the conglomerate material of **2d­[Cu]** were washed with MeOH to give 90 and 75% yield, respectively.

#### Complex **2c­[Cu]**

Mp 277–280 °C dec (MeOH); IR ν 3073, 3049, 3018, 1664, 1622, 1600, 1570, 1458, 1362, 1345, 1126, 1107, 843, 837, 760, 712, 697, 682 cm^–1^; ESI­(+)-MS *m*/*z* 593 (100). Anal. Calcd for C_25_H_14_CuN_5_O_5_: C, 56.87; H, 2.67; N, 13.27. Calcd for C_25_H_14_CuN_5_O_5_·H_2_O: C, 55.00; H, 2.95; N, 12.83. Found: C, 55.18; H, 2.71; N, 12.98.

#### Complex **2d­[Cu]**

Mp 289–291 °C dec (MeOH); IR ν 3085, 3066, 3020, 1672, 1645, 1622, 1598, 1574, 1460, 1428, 1333, 1319, 1272, 1081, 1068, 910, 852, 789, 757, 730, 678 cm^–1^; ESI­(+)-MS *m*/*z* 335 (100). Anal. Calcd for C_25_H_14_CuN_5_O_5_: C, 56.87; H, 2.67; N, 13.27. Calcd for C_25_H_14_CuN_5_O_5_·H_2_O: C, 55.00; H, 2.95; N, 12.83. Found: C, 54.97; H, 2.81; N, 12.82.

### Preparation of Iodides **3c** and **3d**. General Procedure

To a stirred solution of 2-iodophenol (264.0 mg, 1.2 mmol) in dry DMSO (6 mL) was added 60% NaH in mineral oil (96 mg, 2.4 mmol) in one portion. After 15 min, fluoride **6c** or **6d** (226.1 mg, 1.0 mmol) was added, and the reaction mixture was stirred overnight under an Ar atmosphere at 80 °C. The mixture was cooled, diluted with AcOEt (25 mL), and washed with water (3 × 25 mL) and brine (25 mL). The combined organic layers were dried (Na_2_SO_4_), and the solvent was evaporated. The solid residue was absorbed onto SiO_2_ and separated by column chromatography (passivated SiO_2_, CH_2_Cl_2_/AcOEt, 4:1), giving iodides **3c** or **3d** as a yellow solid.

#### 8-(2-Iodophenoxy)-3-(pyridin-3-yl)­benzo­[*e*]­[1,2,4]­triazine (**3c**)

Following the general procedure, iodide **3c** (425 mg, 75% yield) was obtained starting from 350 mg (1.59 mmol) of 2-iodophenol and 300 mg (1.33 mmol) of fluoride **6c**. Mp 186–188 °C (CH_2_Cl_2_/AcOEt); ^1^H NMR (400 MHz, CDCl_3_) δ 9.96 (d, *J* = 1.3 Hz, 1H), 9.06 (dt, *J*_1_ = 8.1 Hz, *J*_2_ = 2.0 Hz, 1H), 8.82 (dd, *J*_1_ = 4.9 Hz, *J*_2_ = 1.7 Hz, 1H), 7.95 (dd, *J*_1_ = 7.9 Hz, *J*_2_ = 1.6 Hz, 1H), 7.85 (t, *J* = 8.0 Hz, 1H), 7.81 (dd, *J*_1_ = 8.6 Hz, *J*_2_ = 1.6 Hz, 1H), 7.55 (ddd, *J*_1_ = 8.0 Hz, *J*_2_ = 4.8 Hz, *J*_3_ = 0.9 Hz, 1H), 7.42 (td, *J*_1_ = 7.7 Hz, *J*_2_ = 1.6 Hz, 1H), 7.18 (dd, *J*_1_ = 8.1 Hz, *J*_2_ = 1.4 Hz, 1H), 7.03 (td, *J*_1_ = 7.8 Hz, *J*_2_ = 1.5 Hz, 1H), 6.91 (dd, *J*_1_ = 7.3 Hz, *J*_2_ = 1.5 Hz, 1H); ^13^C­{^1^H} NMR (101 MHz, CDCl_3_) δ 158.6, 155.3, 154.2, 152.0, 150.3, 142.1, 140.6, 140.1, 136.36, 136.32, 131.5, 130.3, 127.3, 123.9, 123.0, 121.7, 114.2, 89.9; ESI­(+)-MS *m*/*z* 427 (100, [M + H]^+^); HRMS (ESI-TOF) *m*/*z* [M + H]^+^ calcd for C_18_H_12_IN_4_O: 427.0056, found 427.0064. Anal. Calcd for C_18_H_11_IN_4_O: C, 50.72; H, 2.60; N, 13.15. Found: C, 50.71; H, 2.59; N, 13.14.

#### 8-(2-Iodophenoxy)-3-(pyridin-4-yl)­benzo­[*e*]­[1,2,4]­triazine (**3d**)

Following the general procedure, **3d** (397 mg, 70% yield) was obtained starting from 350 mg (1.59 mmol) of 2-iodophenol and 300 mg (1.33 mmol) of **6d**. Mp 148–150 °C (CH_2_Cl_2_/AcOEt); ^1^H NMR (400 MHz, CDCl_3_) δ 8.91 (d, *J* = 6.2 Hz, 2H), 8.71 (dd, *J*_1_ = 4.6 Hz, *J*_2_ = 1.6 Hz, 2H), 7.98 (dd, *J*_1_ = 7.9 Hz, *J*_2_ = 1.6 Hz, 1H), 7.91 (t, *J* = 8.0 Hz, 1H), 7.85 (dd, *J*_1_ = 8.6 Hz, *J*_2_ = 1.6 Hz, 1H), 7.45 (td, *J*_1_ = 7.5 Hz, *J*_2_ = 1.5 Hz, 1H), 7.21 (dd, *J*_1_ = 8.0 Hz, *J*_2_ = 1.4 Hz, 1H), 7.06 (td, *J*_1_ = 7.7 Hz, *J*_2_ = 1.5 Hz, 1H), 6.97 (dd, *J*_1_ = 7.3 Hz, *J*_2_ = 1.5 Hz, 1H); ^13^C­{^1^H} NMR (101 MHz, CDCl_3_) δ 158.1, 155.2, 154.3, 149.9, 144.0, 142.1, 140.7, 140.4, 136.6, 130.4, 127.5, 123.1, 122.9, 121.9, 114.7, 89.9; ESI­(+)-MS *m*/*z* 427 (100, [M + H]^+^); HRMS (ESI-TOF) *m*/*z* [M + H]^+^ calcd for C_18_H_12_IN_4_O: 427.0056, found 427.0064. Anal. Calcd for C_18_H_11_IN_4_O: C, 50.72; H, 2.60; N, 13.15. Found: C, 50.70; H, 2.58; N, 13.15.

### Preparation of Precursors **5c** and **5d**. General Procedure

A solution of 2,3-difluoronitrobenzene (159.1 mg, 1.0 mmol) and nicotinic acid hydrazide (**4c**) or isonicotinic acid hydrazide (**4d**, 137.1 mg, 1.0 mmol) in dry DMSO (4 mL) was stirred overnight under an Ar atmosphere at 100 °C. The mixture was allowed to cool and poured into water (20 mL). The resulting yellow solid was extracted with EtOAc in triplicate; the combined extracts were washed with water and dried (Na_2_SO_4_). The solvent was evaporated to yield the crude product, which was recrystallized (MeOH/H_2_O) to give **5c** or **5d** as a yellow solid.

#### *N*′-(2-Fluoro-6-nitrophenyl)­nicotinohydrazide (**5c**)

Following the general procedure, hydrazide **5c** (3.53 g, 85% yield) was obtained starting from 2.07 g (15.0 mmol) of **4c** and 2.40 g (15.0 mmol) of 2,3-difluoronitrobenzene. Mp 152–155 °C (MeOH/H_2_O); ^1^H NMR (400 MHz, DMSO-*d*_6_) δ 11.03 (s, 1H), 8.95 (br s, 1H), 8.74 (br d, *J* = 2.9 Hz, 2H), 8.14 (dt, *J*_1_ = 8.0 Hz, *J*_2_ = 2.0 Hz, 1H), 7.79 (dt, *J*_1_ = 8.5 Hz, *J*_2_ = 1.5 Hz, 1H), 7.56–7.51 (m, 2H), 7.03 (td, *J*_1_ = 8.3 Hz, *J*_2_ = 4.8 Hz, 1H); ^13^C­{^1^H} NMR (101 MHz, DMSO-*d*_6_) δ 165.0, 152.7 (d, ^1^ *J*_F–C_ = 247.6 Hz), 152.6, 148.4, 138.3 (d, ^3^ *J*_F–C_ = 3.5 Hz), 135.2, 133.9 (d, ^2^ *J*_F–C_ = 10.6 Hz), 127.9, 123.7, 121.4 (d, ^2^ *J*_F–C_ = 20.4 Hz), 121.2 (d, ^3^ *J*_F–C_ = 3.3 Hz), 119.7 (d, ^4^ *J*_F–C_ = 8.5 Hz); ESI­(+)-MS *m*/*z* 277 (100, [M + H]^+^); HRMS (ESI-TOF) *m*/*z* [M + H]^+^ calcd for C_12_H_10_FN_4_O_3_: 277.0737, found 277.0740. Anal. Calcd for C_12_H_9_FN_4_O_3_: C, 52.18; H, 3.28; N, 20.28. Found: C, 51.99; H, 3.21; N, 20.30.

#### *N*′-(2-Fluoro-6-nitrophenyl)­isonicotinohydrazide (**5d**)

Following the general procedure, hydrazide **5d** (2.71 g, 66% yield) was obtained starting from 2.07 g (15.0 mmol) of **4d** and 2.40 g (15.0 mmol) of 2,3-difluoronitrobenzene. Mp 130–134 °C (MeOH/H_2_O); ^1^H NMR (400 MHz, DMSO-*d*_6_) δ 11.13 (s, 1H), 8.78–8.75 (m, 3H), 7.80 (d, *J* = 8.5 Hz, 1H), 7.71 (dd, *J*_1_ = 4.4 Hz, *J*_2_ = 1.7 Hz, 2H), 7.53 (ddd, *J*_1_ = 12.6 Hz, *J*_2_ = 8.2 Hz, *J*_3_ = 1.4 Hz, 1H), 7.03 (td, *J*_1_ = 8.3 Hz, *J*_2_ = 4.8 Hz, 1H); ^13^C­{^1^H} NMR (101 MHz, DMSO-*d*_6_) δ 164.9, 152.7 (d, ^1^ *J*_F–C_ = 247.6 Hz), 150.4, 139.2, 138.3 (d, ^3^ *J*_F–C_ = 3.6 Hz), 133.7 (d, ^2^ *J*_F–C_ = 10.5 Hz), 121.5 (d, ^2^ *J*_F–C_ = 20.2 Hz), 121.3, 121.2 (d, ^3^ *J*_F–C_ = 3.3 Hz), 119.7 (d, ^4^ *J*_F–C_ = 8.5 Hz); ESI­(+)-MS *m*/*z* 277 (100, [M + H]^+^); HRMS (ESI-TOF) *m*/*z* [M + H]^+^ calcd for C_12_H_10_FN_4_O_3_: 277.0737, found 277.0738. Anal. Calcd for C_12_H_9_FN_4_O_3_: C, 52.18; H, 3.28; N, 20.28. Found: C, 52.19; H, 3.27; N, 20.29.

### Preparation of Fluorides **6c** and **6d**. General Procedure

To a stirred solution of hydrazide **5c** or **5d** (276.0 mg, 1.0 mmol) in EtOH (5 mL), SnCl_2_·2H_2_O (1128.5 mg, 5.0 mmol) was added in a single portion, and the reaction mixture was refluxed overnight. Upon reaction completion, the reaction mixture was cooled and quenched in saturated NaHCO_3_ solution (50 mL) and then extracted with EtOAc (3 × 25 mL). The combined organic layers were washed with additional saturated NaHCO_3_ solution (30 mL). The combined organic layers were dried (Na_2_SO_4_), and the solvent was evaporated. The solid residue was absorbed onto SiO_2_ and separated by column chromatography (SiO_2_, 10–60% AcOEt/CH_2_Cl_2_), giving **6c** or **6d** as a yellow solid.

#### 8-Fluoro-3-(pyridin-3-yl)­benzo­[*e*]­[1,2,4]­triazine (**6c**)

Following the general procedure, fluoride **6c** (1.22 g, 45% yield) was obtained starting from 3.29 g (11.90 mmol) of hydrazide **5c** and 13.77 g (60.70 mmol) of SnCl_2_·2H_2_O. Mp 170–172 °C (CH_2_Cl_2_/AcOEt); ^1^H NMR (400 MHz, CDCl_3_) δ 9.97 (d, *J* = 1.3 Hz, 1H), 9.11 (dt, *J*_1_ = 8.1 Hz, *J*_2_ = 2.0 Hz, 1H), 8.85 (dd, *J*_1_ = 4.9 Hz, *J*_2_ = 1.7 Hz, 1H), 8.03–7.97 (m, 2H), 7.62–7.54 (m, 2H); ^13^C­{^1^H} NMR (101 MHz, CDCl_3_) δ 158.5, 158.1 (d, ^1^ *J*_F–C_ = 270.2 Hz), 151.6, 149.8, 141.7, 138.6 (d, ^3^ *J*_F–C_ = 12.7 Hz), 137.0, 136.2 (d, ^3^ *J*_F–C_ = 8.6 Hz), 131.5, 125.2 (d, ^4^ *J*_F–C_ = 5.2 Hz), 124.2, 114.9 (d, ^2^ *J*_F–C_ = 17.9 Hz); ESI­(+)-MS *m*/*z* 227 (100, [M + H]^+^); HRMS (ESI-TOF) *m*/*z* [M + H]^+^ calcd for C_12_H_8_FN_4_: 227.0733, found 227.0738. Anal. Calcd for C_12_H_7_FN_4_: C, 63.71; H, 3.12; N, 24.77. Found: C, 63.58; H, 3.05; N, 24.91.

#### 8-Fluoro-3-(pyridin-4-yl)­benzo­[*e*]­[1,2,4]­triazine (**6d**)

Following the general procedure, **6d** (1.64 g, 74% yield) was obtained starting from 2.69 g (9.70 mmol) of hydrazide **5d** and 11.22 g (49.70 mmol) of SnCl_2_·2H_2_O. Mp 178–180 °C (CH_2_Cl_2_/AcOEt); ^1^H NMR (400 MHz, CDCl_3_) δ 8.91 (dd, *J*_1_ = 4.5 Hz, *J*_2_ = 1.6 Hz, 2H), 8.62 (dd, *J*_1_ = 4.5 Hz, *J*_2_ = 1.6 Hz, 2H), 8.05–7.97 (m, 2H), 7.59 (ddd, *J*_1_ = 9.0 Hz, *J*_2_ = 7.3 Hz, *J*_3_ = 1.6 Hz, 1H); ^13^C­{^1^H} NMR (101 MHz, CDCl_3_) δ 158.6, 158.0 (d, ^1^ *J*_F–C_ = 270.2 Hz), 150.9, 142.7, 141.7, 138.8 (d, ^3^ *J*_F–C_ = 12.9 Hz), 136.2 (d, ^3^ *J*_F–C_ = 8.6 Hz), 125.4 (d, ^4^ *J*_F–C_ = 5.3 Hz), 122.5, 115.3 (d, ^2^ *J*_F–C_ = 17.9 Hz); ESI­(+)-MS *m*/*z* 227 (100, [M + H]^+^); HRMS (ESI-TOF) *m*/*z* [M + H]^+^ calcd for C_12_H_8_FN_4_: 227.0733, found 227.0734. Anal. Calcd for C_12_H_7_FN_4_: C, 63.71; H, 3.12; N, 24.77. Found: C, 63.68; H, 3.16; N, 24.85.

## Supplementary Material



## Notes

NMR, IR, UV–vis, and EPR methods and spectra, XRD data collection and SQUID magnetization study details and analysis, and details of DFT calculations (PDF)
