# Supplementary material for: Ladder-Type Cu(II) Coordination Polymer with π–π Stacking of Planar Blatter Radical Ligands: Structural and Magnetic Characterization
Source: Cryst Growth Des. 2025 Sep 17;25(19):8289–99. doi: 10.1021/acs.cgd.5c01115 (PMC12492399; doi:10.1021/acs.cgd.5c01115)
Supplement: Supplementary file 1 [file cg5c01115_si_001.pdf]

Ladder-type Cu(II) coordination polymer with π–π stacking of planar Blatter radical ligands: Structural and magnetic characterization

Hemant K. Singh,[a] Kayla M. Smith,[b] Jeremy M. Rawson,[c] Bruno Camargo,[d] Ethan S. Pollett,[b] Oleksandr Hietsoi, [b]Andrienne C. Friedli,[b] and Piotr Kaszyński* [a,b,e]

[a] Centre of Molecular and Macromolecular Studies, Polish Academy of Sciences, 90363 Łódź, Poland
[b] Department of Chemistry, Middle Tennessee State University, Murfreesboro, TN 37132, USA
[c] Department of Chemistry and Biochemistry, University of Windsor, Windsor, ON, Canada, N9B 3P4
[d] Institute of Experimental Physics, Faculty of Physics, University of Warsaw, 02093 Warsaw, Poland
[e] Faculty of Chemistry, University of Łódź, 91403 Łódź, Poland

Table of Contents **Page**

1. NMR Spectra …S2
2. Solid-state IR spectra …S8
3. XRD data collection and refinement details …S10
   a) crystal preparation …S10
   b) general comments to data collection …S10
   c) structure solution and refinement …S11
   d) data analysis …S12
   e) additional views of packing in ***2c[Cu]*** …S14
4. UV-vis spectroscopy …S15
5. Electrochemical analysis …S17
6. EPR Spectroscopy …S18
7. SQUID magnetometry …S24
8. Computational details and results …S28
   a) isotropic Fermi contact coupling constants (hfcc) and spin densities …S29
   b) spin delocalization in radicals in benzene dielectric medium …S30
   c) electronic excitations …S31
   d) partial output data from TD-DFT calculations …S32
   e) intermolecular interaction energy calculations …S34
9. Archive for DFT geometry optimization results …S36
10. References …S37

1. NMR spectra

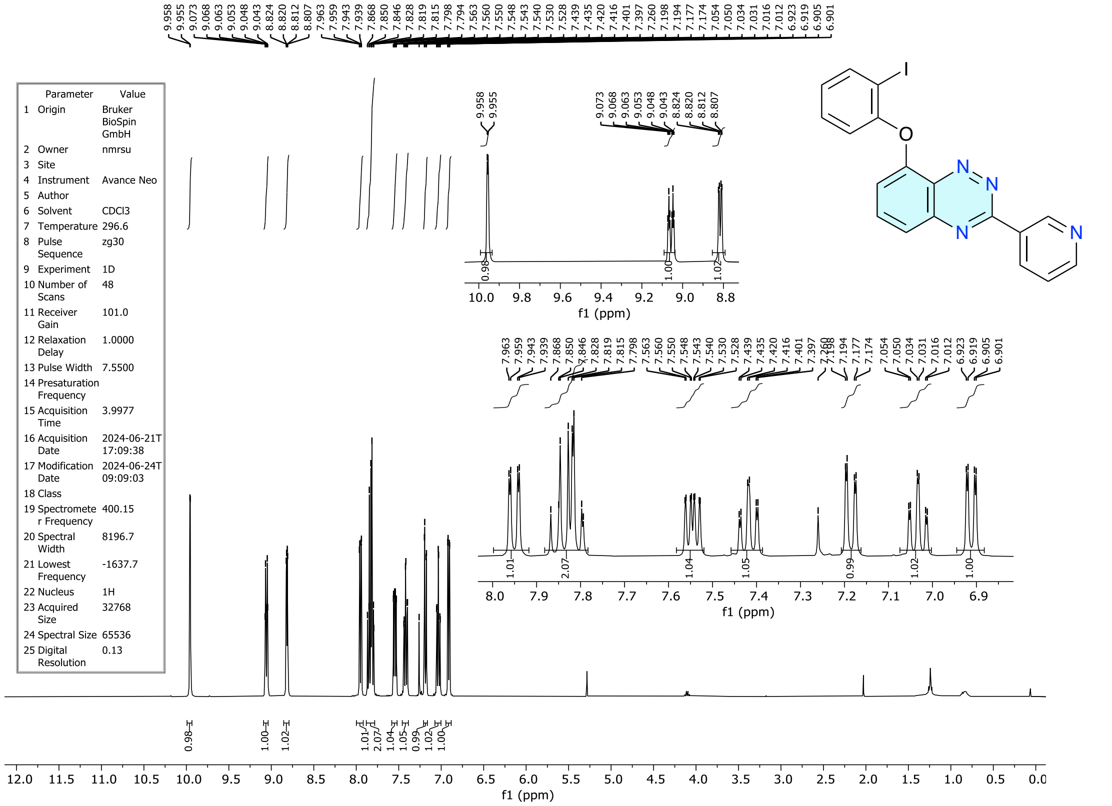

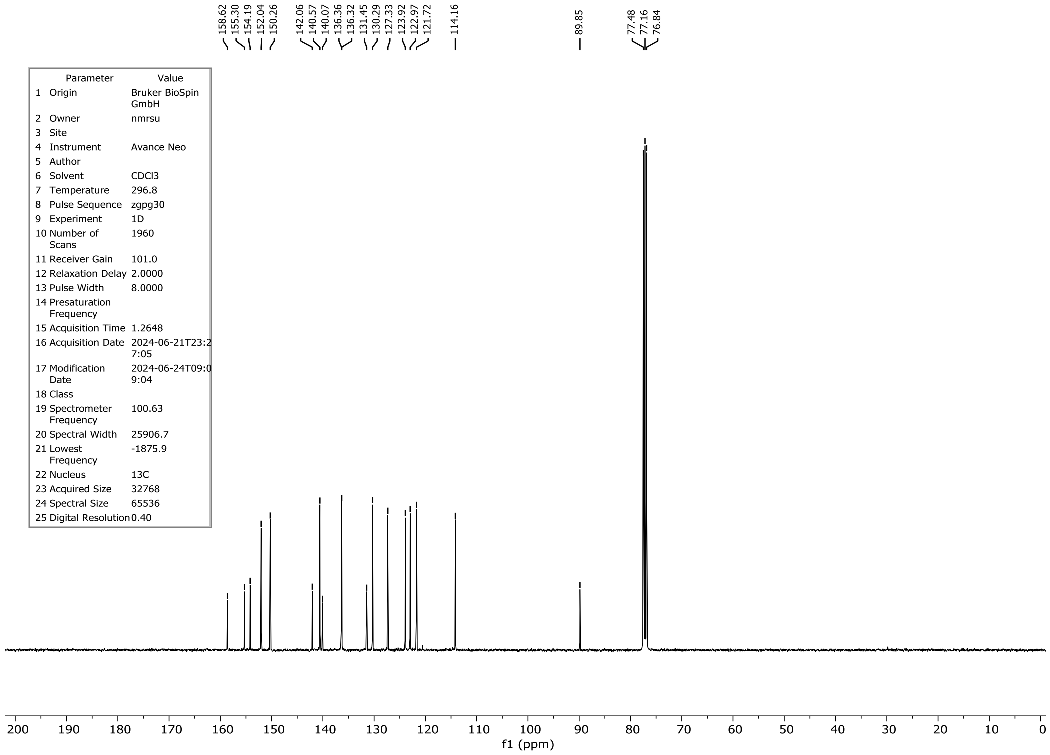

Figure S1. ^{1}H and ^{13}C{^{1}H} NMR of **3c** recorded in $CDCl_3$ at 400 and 101 MHz, respectively.

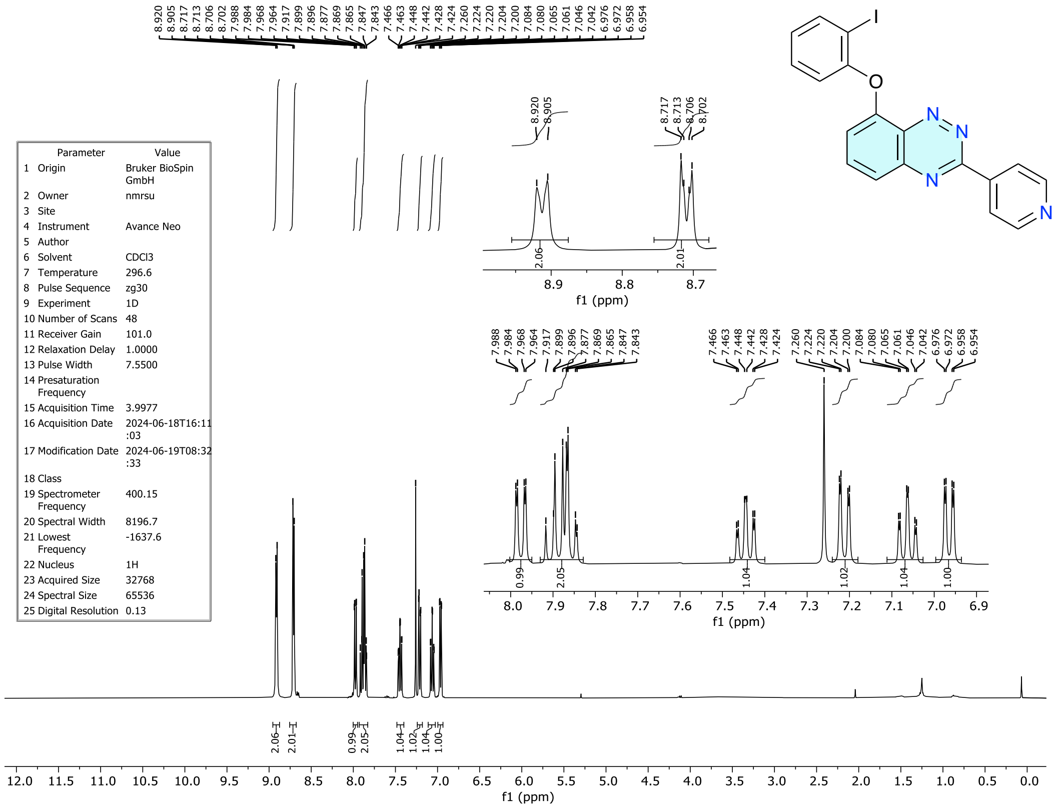

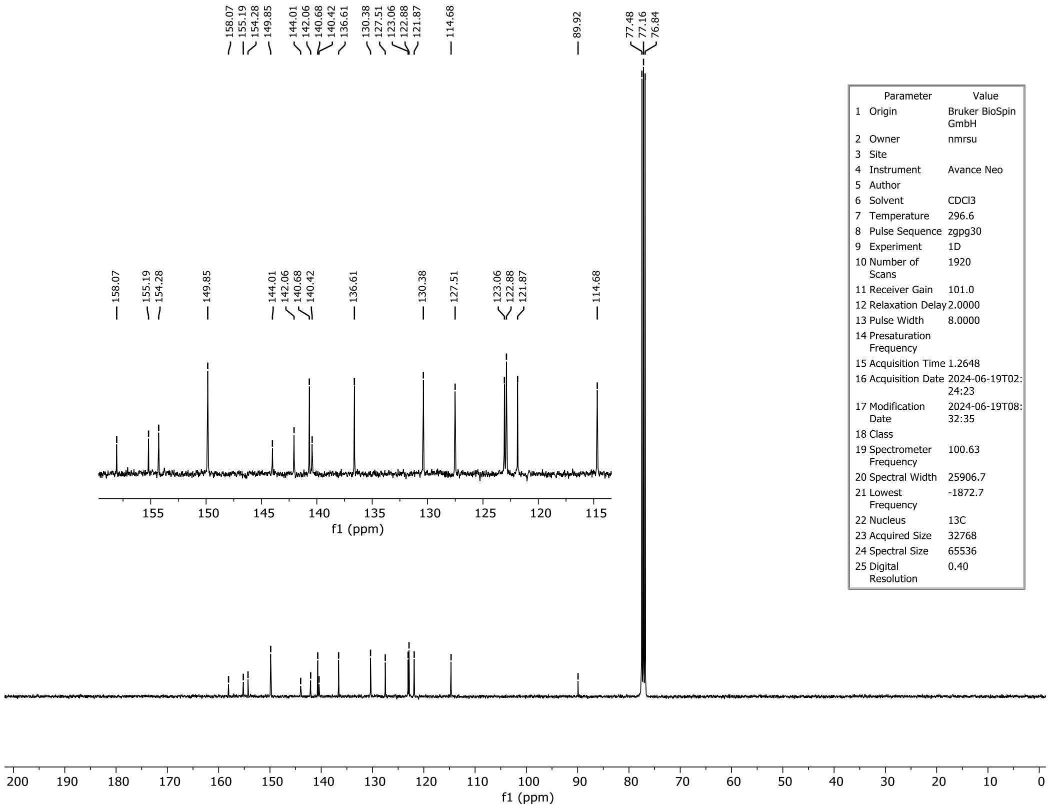

Figure S2. ^{1}H and $^{13}C\{^{1}H\}$ NMR of **3d** recorded in $CDCl_3$ at 400 and 101 MHz, respectively.

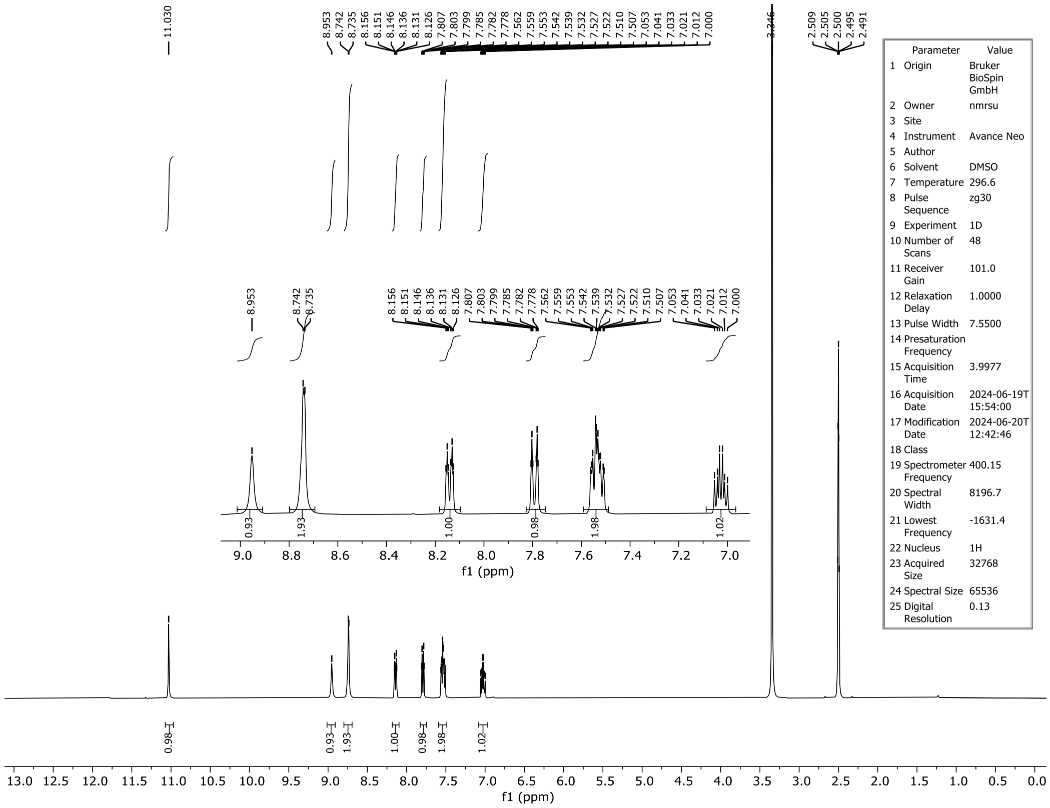

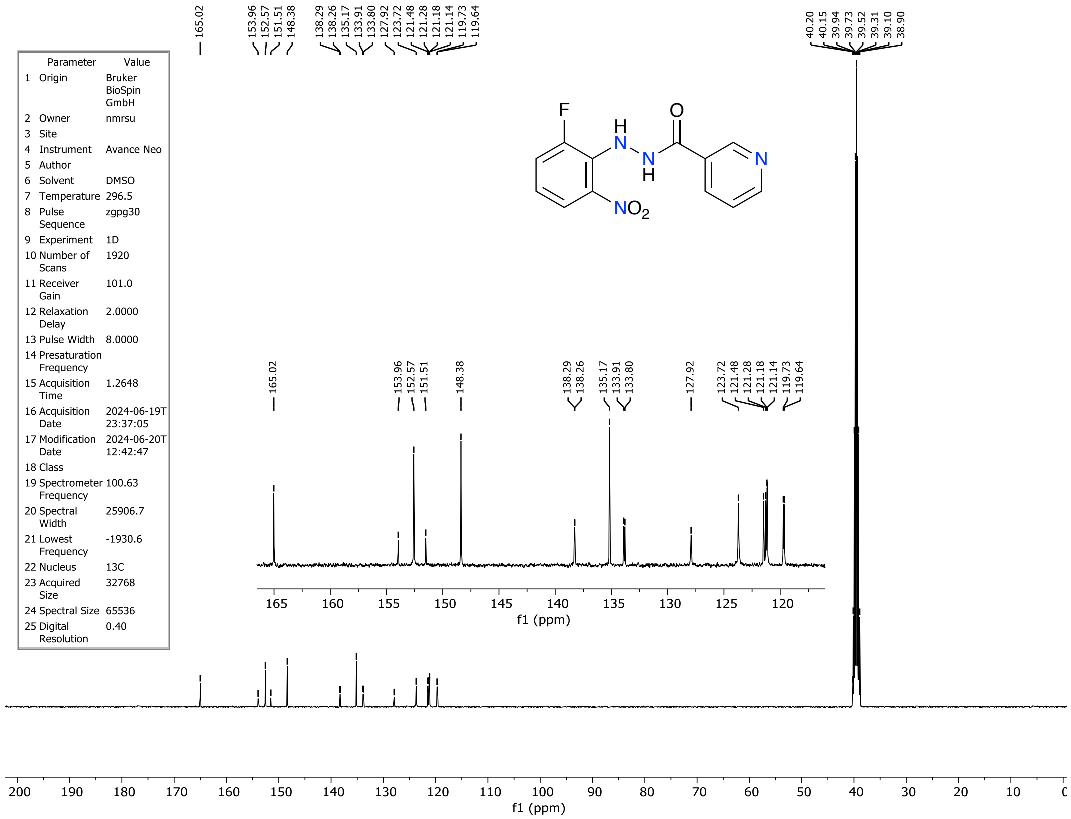

Figure S3. ^{1}H and ^{13}C{^{1}H} NMR of **5c** recorded in DMSO-d_6 at 400 and 101 MHz, respectively.

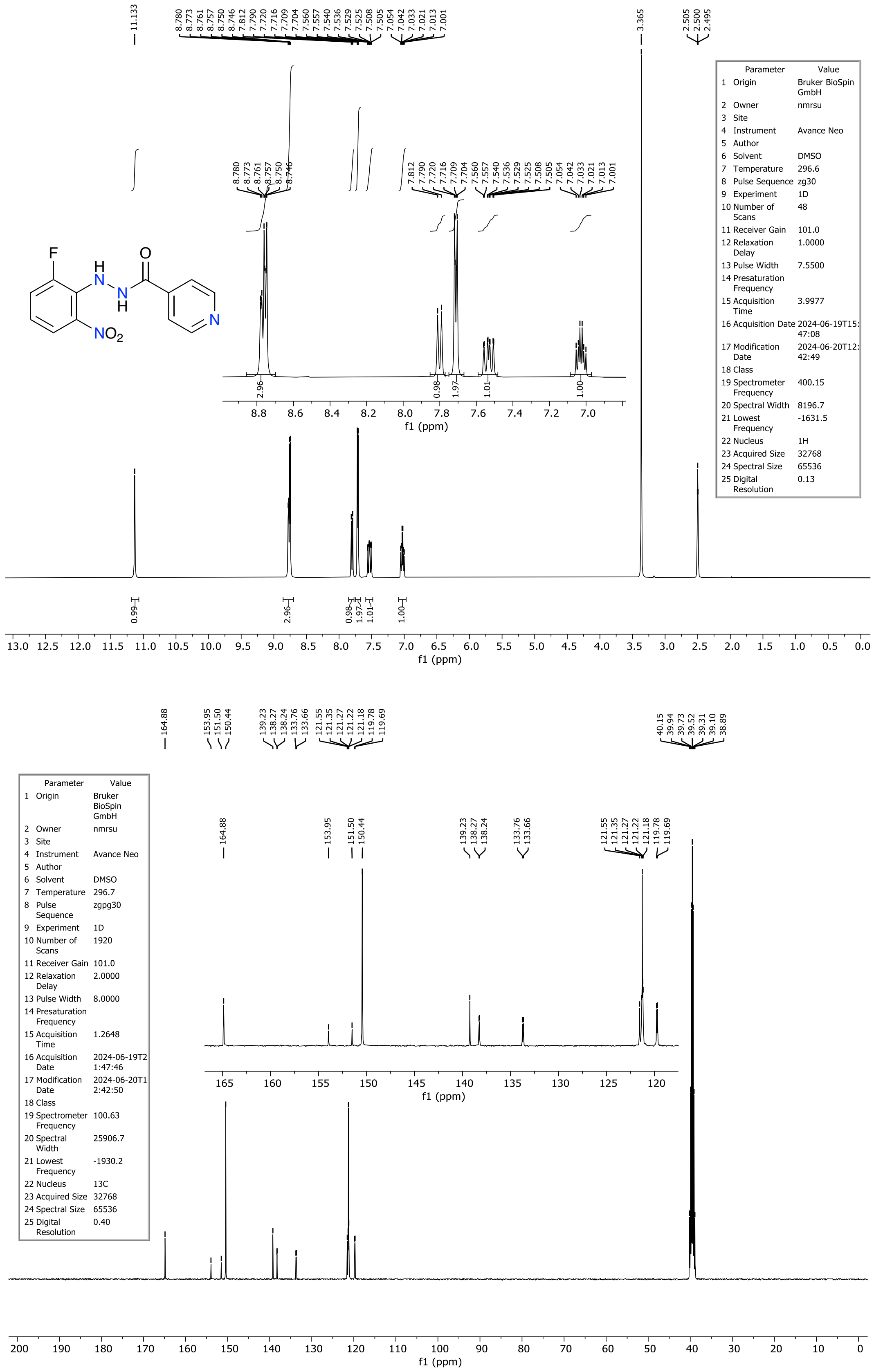

Figure S4. ^{1}H and $^{13}C\{^{1}H\}$ NMR of **5d** recorded in DMSO-d_6 at 400 and 101 MHz, respectively.

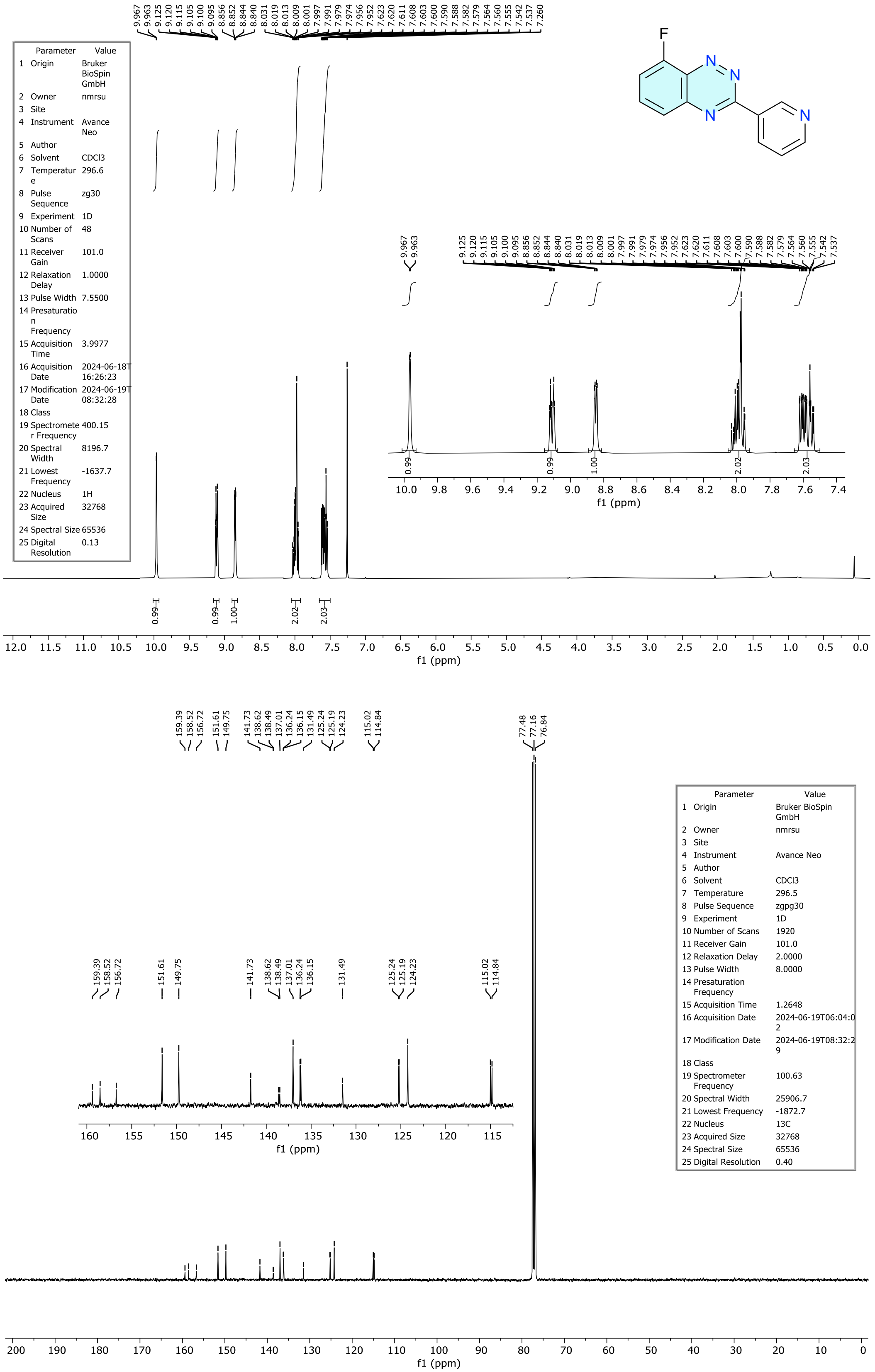

Figure S5. ^{1}H and ^{13}C{^{1}H} NMR of **6c** recorded in $CDCl_3$ at 400 and 101 MHz, respectively.

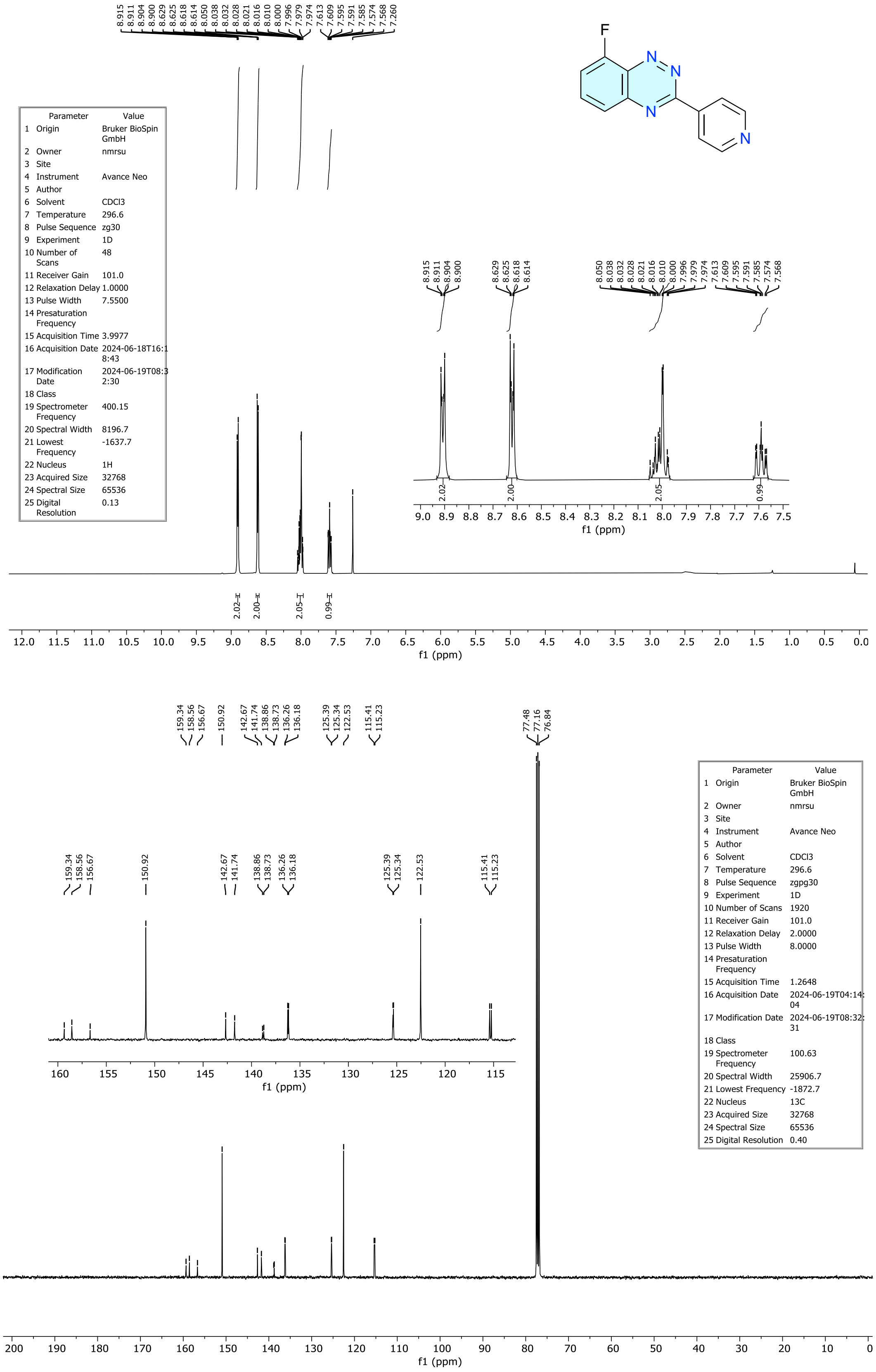

Figure S6. ^{1}H and $^{13}C\{^{1}H\}$ NMR of **6d** recorded in $CDCl_3$ at 400 and 101 MHz, respectively.

2. IR spectra

IR spectra for radicals **2** and their complexes **2[Cu]** were recorded for solid-state samples using a Thermo Scientific Nicolet 6700 FT-IR spectrophotometer and are shown in Figures S7–S10.

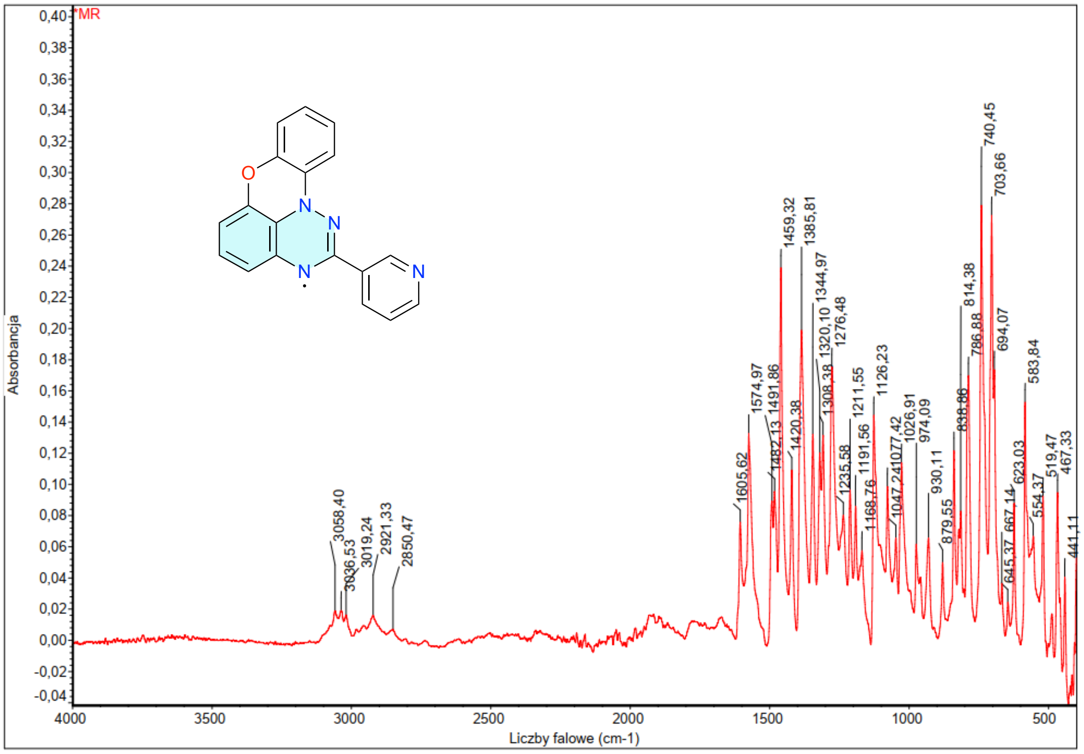

Figure S7. Solid state IR spectrum of radical **2c**.

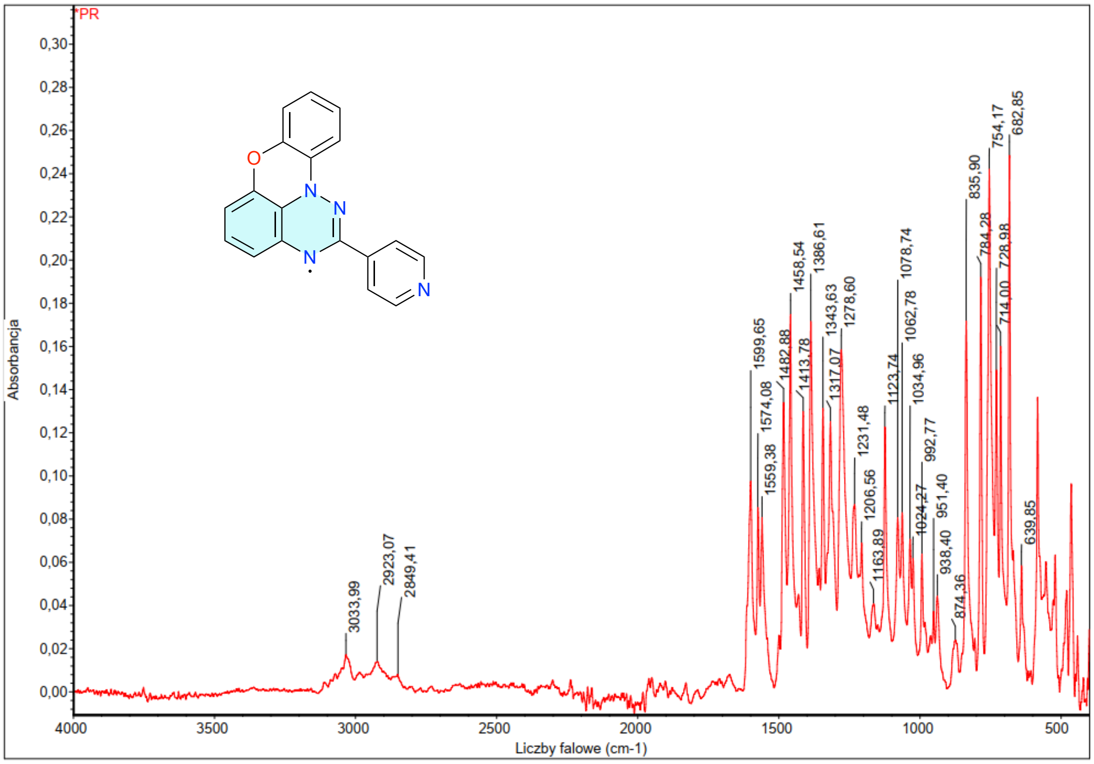

Figure S8. Solid state IR spectrum of radical **2d**.

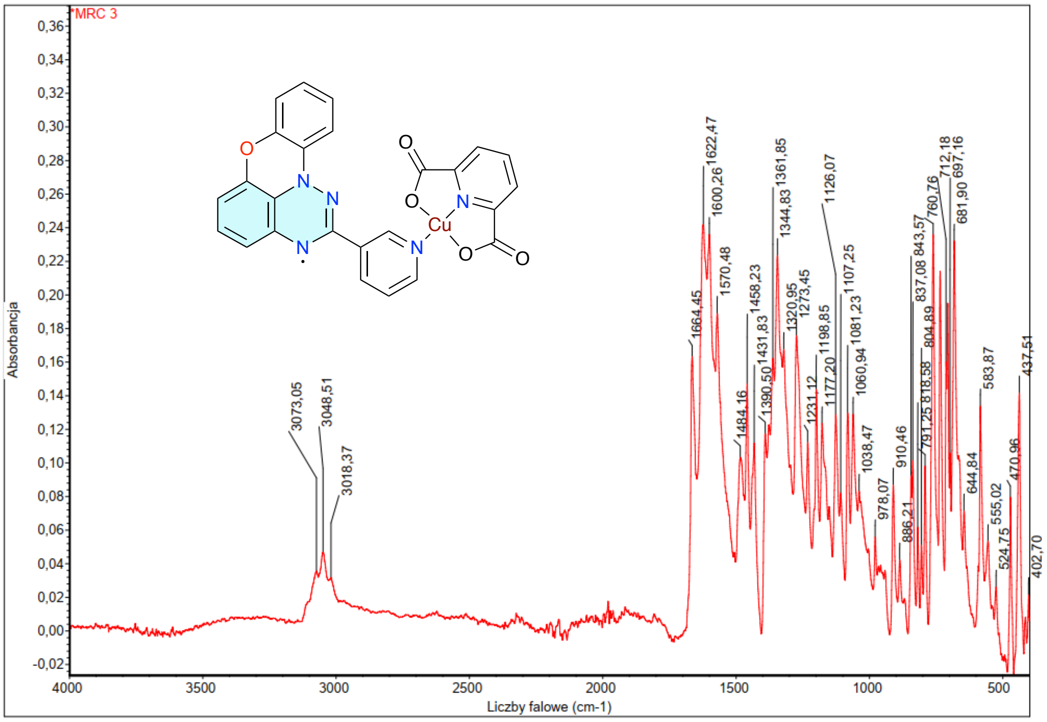

Figure S9. Solid state IR spectrum of complex **2c[Cu]**.

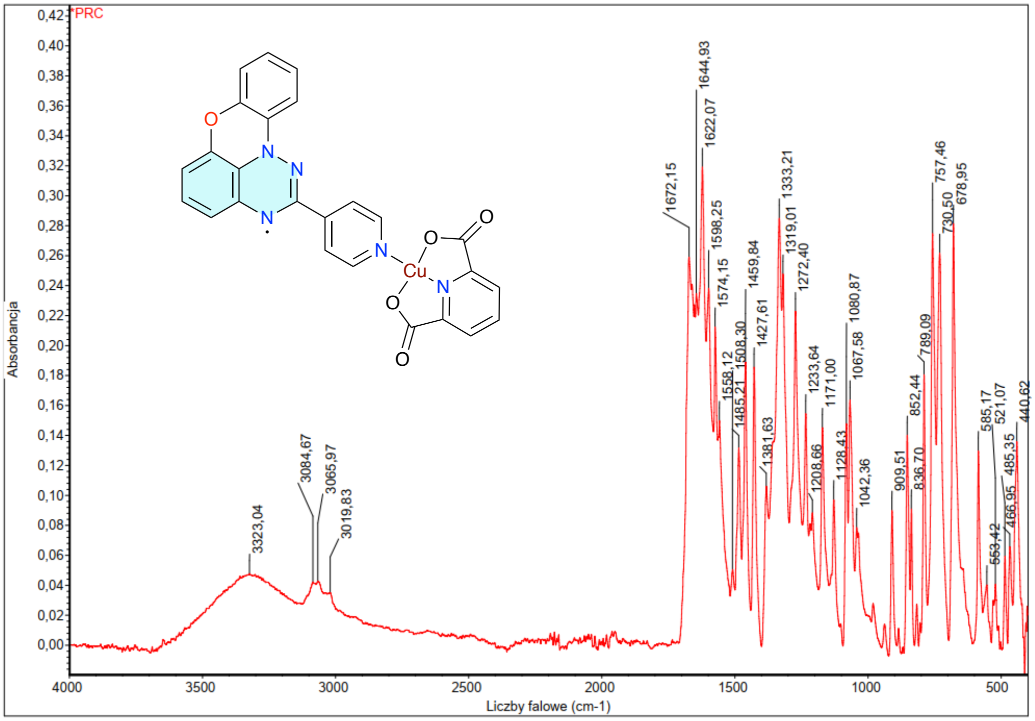

Figure S10. Solid state IR spectrum of complex **2d[Cu]**.

3. XRD data collection and refinement details

a) crystal preparation

Orange prism-shaped crystals suitable for single-crystal XRD analysis were obtained by slow cooling of MeOH/H_2O solutions (**5d**). Yellow plate-shaped crystals were obtained by slow evaporation of CH_2Cl_2 (**3c**) or MeOH solutions (**6c**). Evaporation of MeOH/EtOH/MeCN solutions of **2c[Cu]** gave blue triclinic crystals (*P*-1). All attempts at growing crystals of **2d[Cu]** suitable for XRD analysis by slow evaporation of MeOH, MeCN/MeOH or MeCN/EtOH and other solvent systems were unsuccessful. Similarly, XRD quality crystals of radicals **2c** and **2d** could not be obtained.

b) general comments to data collection

All crystal data were collected on a dual source Rigaku SuperNova diffractometer with a Dectris Pilatus3 R 200 K-A detector, equipped with an Oxford Cryosystems Ltd. nitrogen flow apparatus (Cryostream 800 Series) at 100 K using micro-focus X-ray Source Cu *K*α radiation, λ = 1.54184 Å, (for **2c[Cu]**, **5d**, **6c**) or Mo *K*α radiation λ = 0.71073 Å (for **3c**) The data were integrated using CrysAlisPro program.[1] Intensities for absorption were corrected using multi-scan or gaussian

method as in SCALE3 ABSPACK scaling algorithm implemented in CrysAlisPro program.[1] The crystal data and structure refinement descriptors for all structures are listed in Table S1.

Files CCDC 2457177 and 2463643-2463645 contain supplementary crystallographic data for this paper. These data can be obtained free of charge from the Cambridge Crystallographic Data Centre via www.ccdc.cam.ac.uk/structures.

Table S1. Selected structural data for analyzed crystals

	**2c[Cu]**<br>2457177	**3c**<br>2463644	**5d**<br>2463643	**6c**<br>2463645
Formula	$C_{25}H_{14}CuN_5O_5$	$C_{18}H_{11}IN_4O$	$C_{12}H_9FN_4O_3$	$C_{12}H_7FN_4$
Formula Weight	527.95	426.21	276.23	226.22
Crystal System	triclinic	triclinic	monoclinic	monoclinic
Space Group	P-1	P-1	$P2_1/n$	$P2_1/n$
a/Å	7.6327(6)	7.1532(3)	8.39832(10)	7.1830(3)
b/Å	9.3580(6)	7.5805(2)	16.8578(2)	21.3390(6)
c/Å	14.8044(12)	16.1935(5)	8.54241(10)	7.3020(3)
α/°	92.258(6)	84.961(2)	90	90
β/°	102.579(7)	88.810(3)	100.7180(12)	118.264(6)
γ/°	95.584(6)	63.871(3)	90	90
V/Å^3	1025.15(14)	785.15(5)	1188.31(3)	985.79(8)
Z	2	2	4	4
2θ range for data collection /°	3.06 to 76.99	2.53 to 29.79	5.89 to 76.38	4.14 to 76.66
No. of measured, independent, and observed [I>2σ(I)] reflections	9235, 4110, 2362	8297, 3530, 3413	5622, 2397, 2328	7477, 2002, 1745
R_{int}	0.0741	0.0355	0.0120	0.0321
Goodness-of-fit on F^2	1.032	1.038	1.029	1.124
Final R indexes [$F^2 > 2σ(F^2)$]	R_1 = 0.0853, wR_2 = 0.2097	R_1 = 0.0232, wR_2 = 0.0586	R_1 = 0.0382, wR_2 = 0.0994	R_1 = 0.0521, wR_2 = 0.1556
Final R indexes [all data]	R_1 = 0.1459, wR_2 = 0.2496	R_1 = 0.0240, wR_2 = 0.0591	R_1 = 0.0390, wR_2 = 0.1000	R_1 = 0.0521, wR_2 = 0.1588
Restraints/parameters	0/319	0/217	0/181	0/154
Largest diff. peak/hole Å^{-3}	1.219/--0.722	0.898/-1.061	0.481/-0.524	0.317/-0.226

c) structure solution and refinement

Structures were solved with the ShelXT structure solution program[2] and refined in the ShelXL program[3] by the full-matrix least-squares minimization on F^2 using OLEX2 software package.[4]

All non-hydrogen atoms were refined anisotropically. All hydrogen atoms were included in idealized positions for structure factor calculations using a riding model. Structural data are collected in Table S1.

d) data analysis

Iodide **3c** crystallizes in the triclinic space group *P*-1 and two molecules in the unit cell (Figure S11). The geometry and dimensions of the benzo[*e*][1,2,4]triazine system are typical for this ring system.[5] The C(3)-Ph group is nearly coplanar with the benzo[*e*][1,2,4]triazine system with the angle between the mean planes of 3.8°. The iodophenyl and benzo[*e*][1,2,4]triazine mean planes form an angle of 66.4°, or the C_{BT}–C_{BT}–O–C_{Ph} torsion angle of 67.2(3)°. Benzo[*e*][1,2,4]triazine mean planes are separated by 3.253 Å in the discrete dimer in the unit cell (Figure S11) and by 3.651 Å between the unit cells. The discrete dimers form slipped stacks separated by the iodophenyl groups.

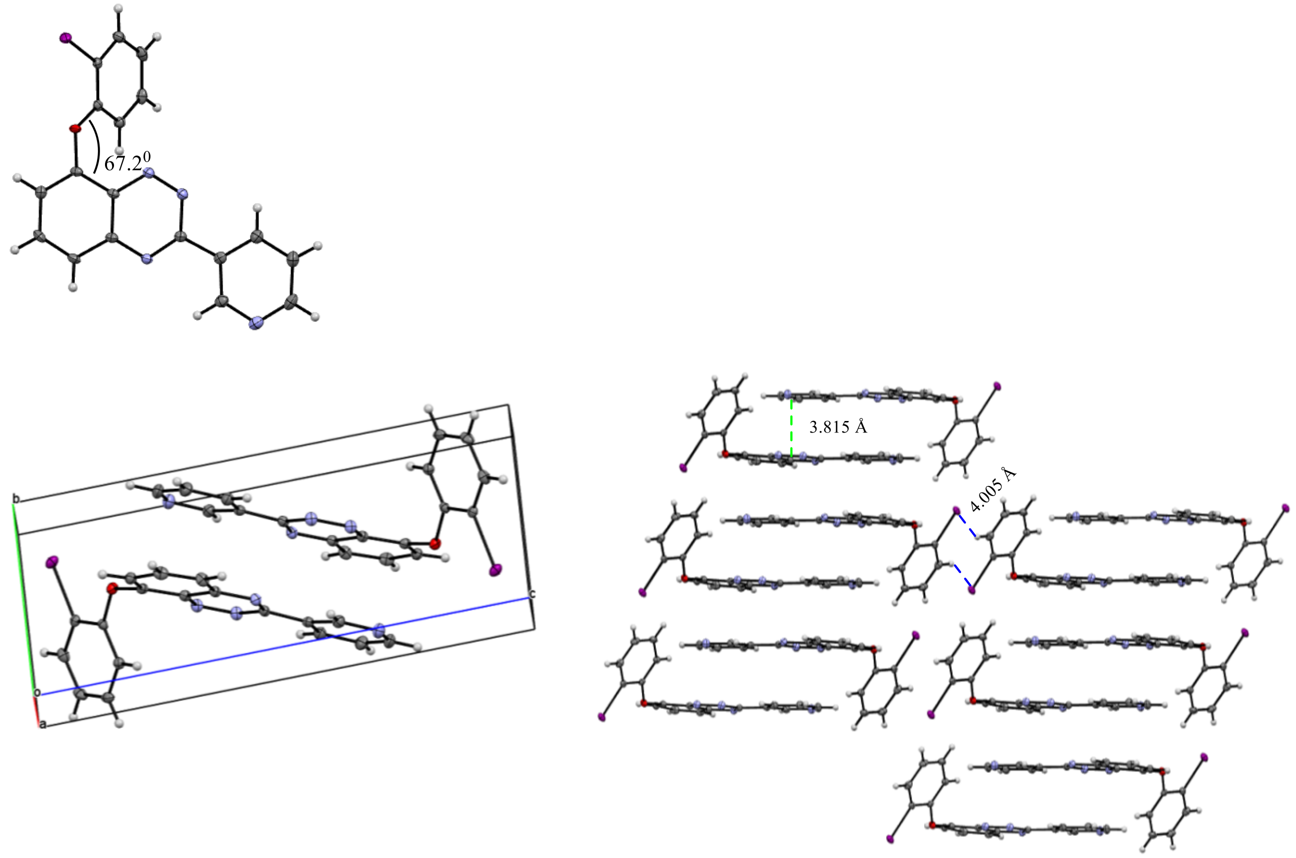

Figure S11. Molecular structure (top) and unit cell and partial packing diagram of **3c** (bottom). Displacement ellipsoids are drawn at 50% probability level.

Hydrazide **5d** crystallizes in the monoclinic crystal system with the space group $P2_1$/n. The unit cell contains four molecules related by symmetry. The hydrazine β H atom forms intramolecular and intermolecular hydrogen bonds to the NO_2 group with the O---H distance of 2.037 and 2.308 Å, respectively (Figure S12). The molecules form a zig-zag pattern in the crystal structure.

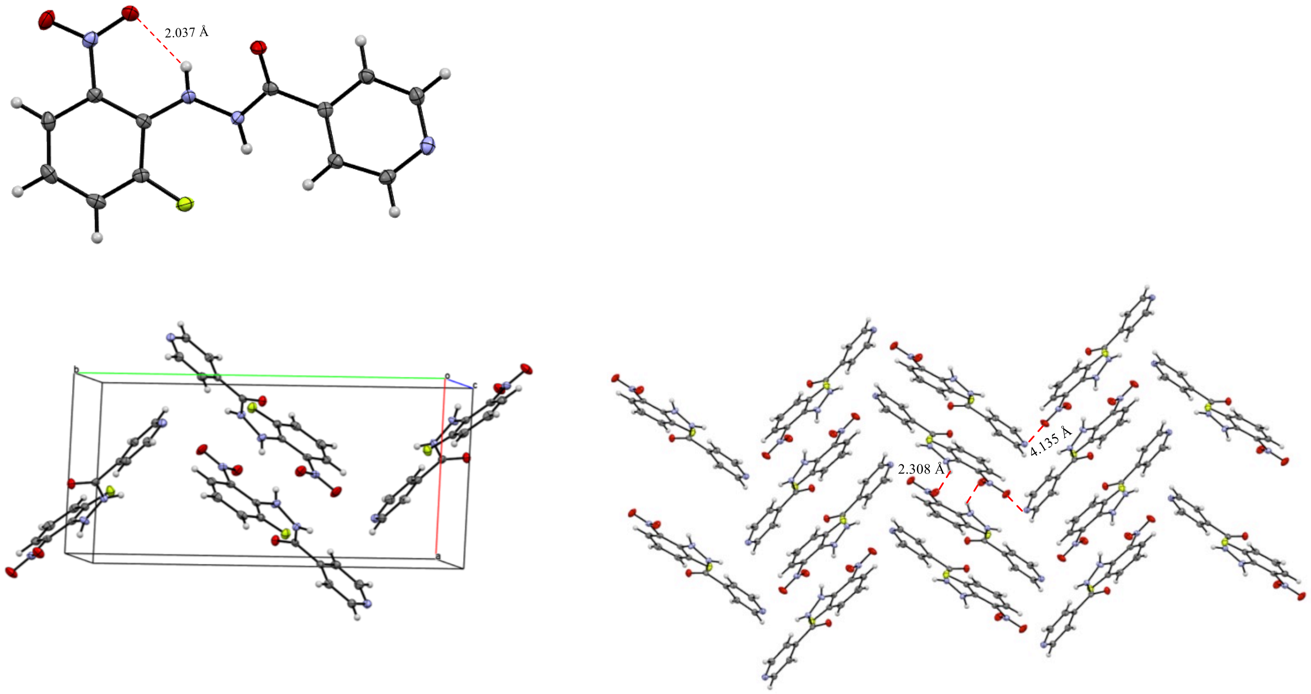

Figure S12. Molecular structure (top) and unit cell and partial packing diagram of **5d** (bottom). Displacement ellipsoids are drawn at 50% probability level.

Fluoride **6c** crystallize in the monoclinic crystal system with the $P2_1/n$ space group. The unit cell contains four molecules. The geometry and dimensions of the benzo[e][1,2,4]triazine system are typical for this ring system.[5] The C(3)-Ph group is nearly coplanar with the benzo[*e*][1,2,4]triazine system with the angle between the mean planes of 1.8° (Figure S13). Two symmetry related molecules in the unit cell form a discrete dimer with the separation between the mean molecular planes of 3.30 Å. The dimers form slipped stacks with the separation of 3.43 Å. There are H bonds C-H---N and C-H---F with distances of 2.523 and 2.298 Å, respectively. The F---F separation is 4.159(1) Å.

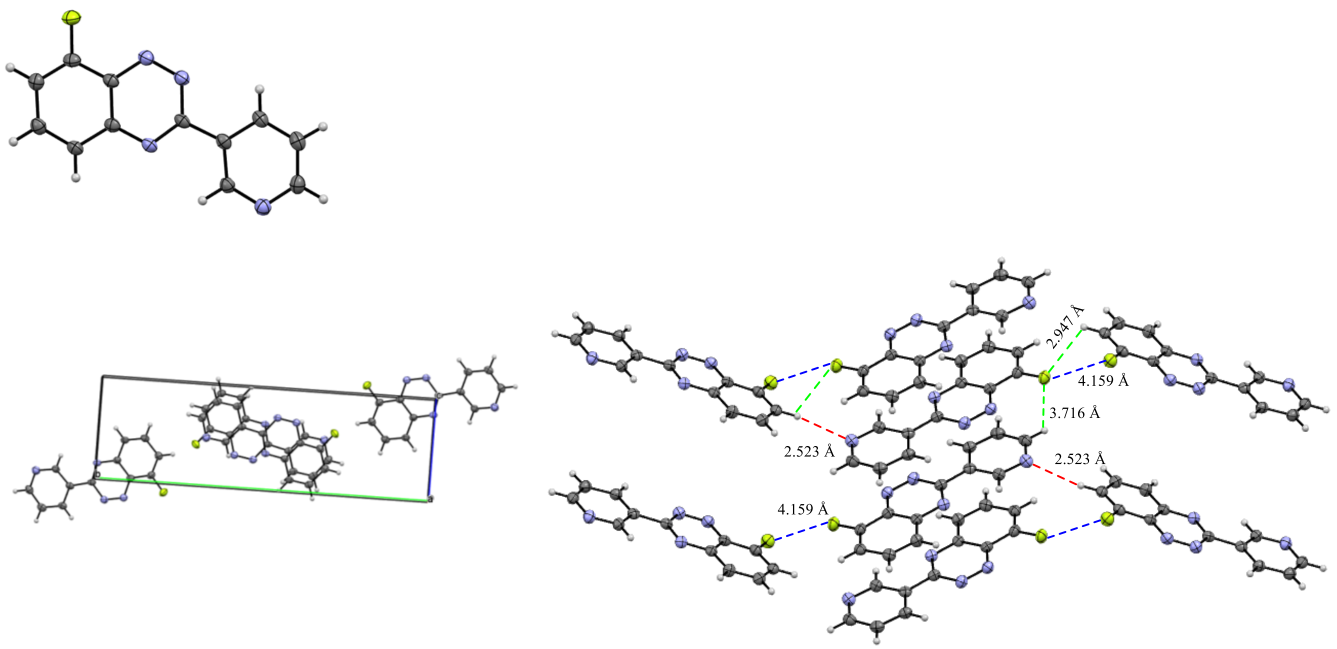

Figure S13. Molecular structure (top) and unit cell and partial packing diagram of **6c** (bottom). Displacement ellipsoids are drawn at 50% probability level.

e) additional views of packing in 2c[Cu]

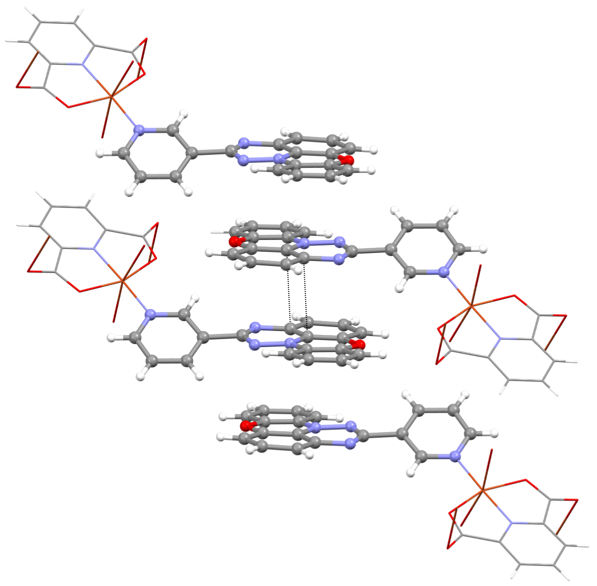

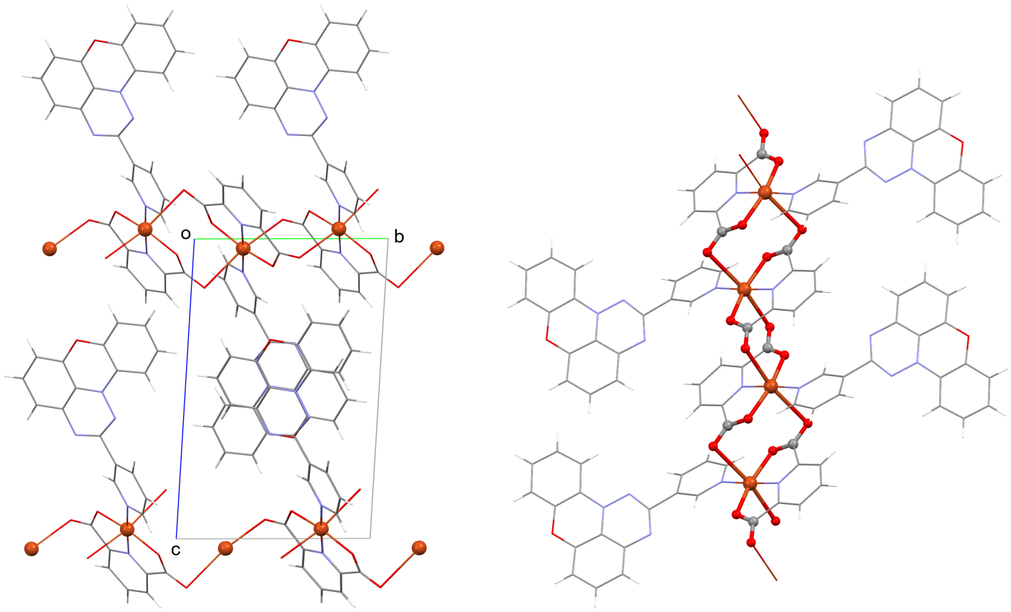

Figure S14. Additional views for molecular packing in the solid state of **2c[Cu]**.

4. UV-vis spectroscopy

Electronic absorption spectra were recorded on a Jasco V770 spectrophotometer in spectroscopic grade CH_2Cl_2 at concentrations in the range 2–10×10^{-5} M for radicals **2c** and **2d** and fitted to the Beer–Lambert law. Results are shown in Figures S15 and S16.

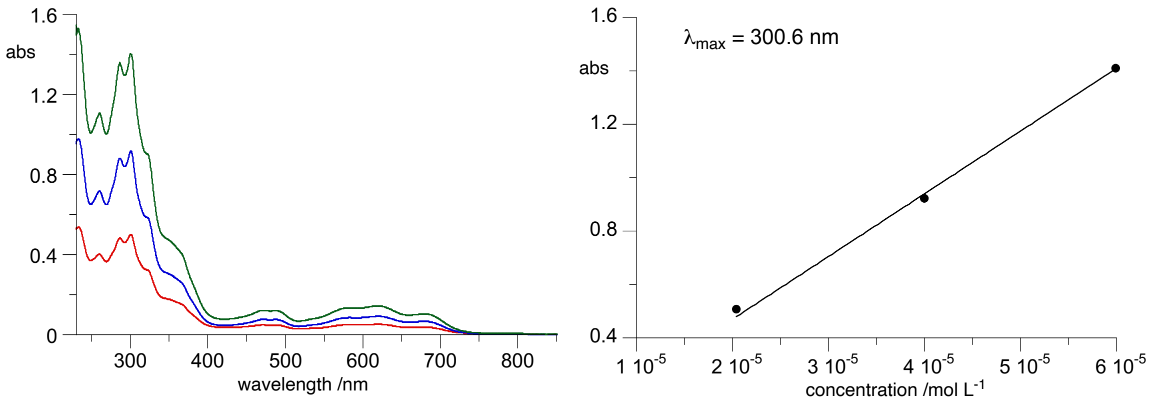

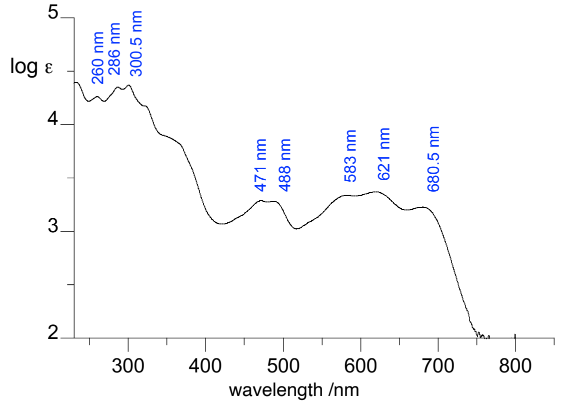

Figure S15. Clockwise: electronic absorption spectra for **2c** in CH_2Cl_2 for three concentrations, determination of molar extinction coefficient ε at λ = 300.6 nm (best fit function: ε = 23343(280)×conc, r^2 = 0.999), and molar excitation *log* (ε).

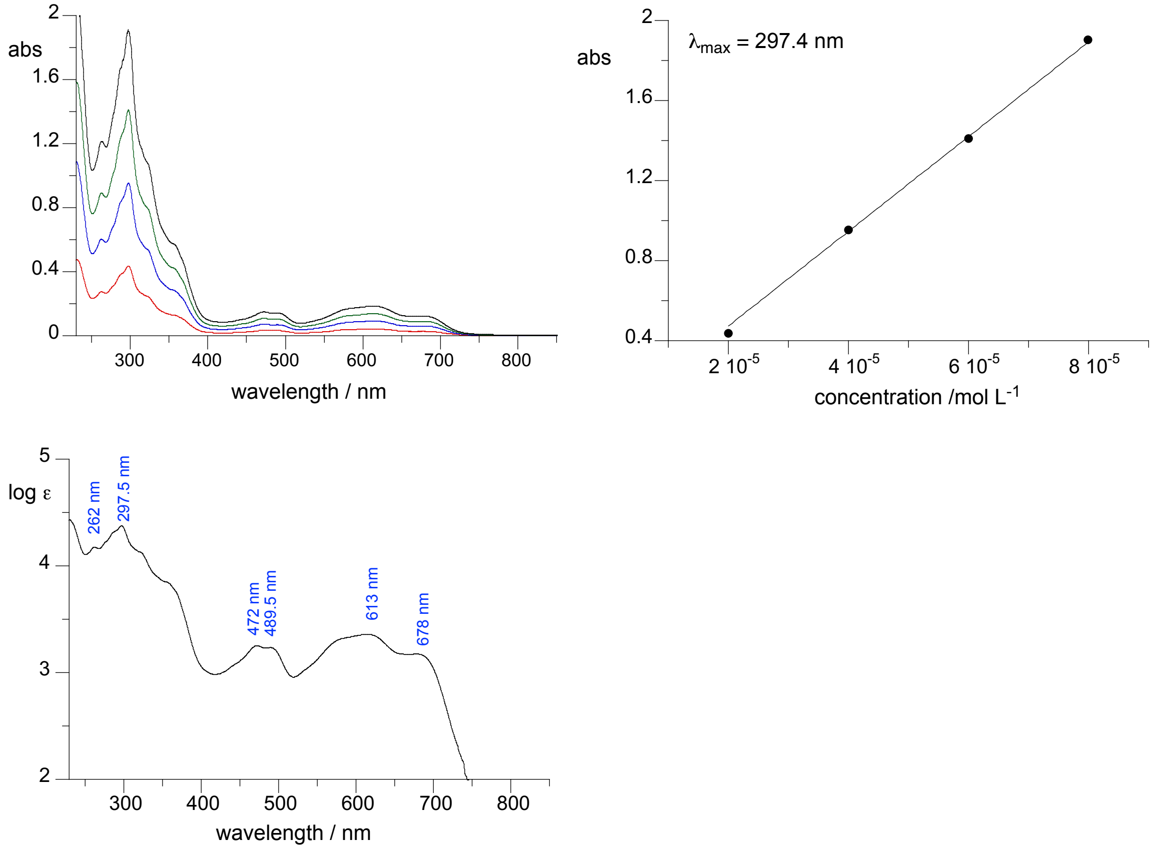

Figure S16. Clockwise: electronic absorption spectra for **2d** in CH_2Cl_2 for four concentrations, determination of molar extinction coefficient ε at λ = 297.4 nm (best fit function: ε = 23657(217)×conc, r^2 = 0.999), and molar excitation *log* (ε).

5. Electrochemical analysis

Electrochemical characterization of selected radicals was conducted using a Metrohm Autolab PGSTAT128N potentiostat/galvanostat instrument. Radicals **2** were dissolved in dry CH_2Cl_2 (conc. 1 mM) in the presence of $Bu_4N^+PF_6^-$ as an electrolyte (conc. 100 mM) and the resulting solution was degassed by purging with Ar gas for 20 min. A three-electrode electrochemical cell was used with glassy carbon disk as the working electrode (ϕ 2 mm, alumina polished), Pt wire as the counter electrode and Ag/AgCl wire as the pseudo reference electrode. All samples were measured without internal reference once and afterwards with $Me_{10}Fc$ (-0.556 V *vs* the Fc/Fc^+ couple) as the internal reference with a scan rate of 50 mV s^{-1} at *ca.* 20 °C. Cyclic voltammetry (CV) measurements were started from 0.0 V in the oxidative direction. Plots are shown in Figures S17–S19, while the data are collected in Table S2 and presented in the main text.

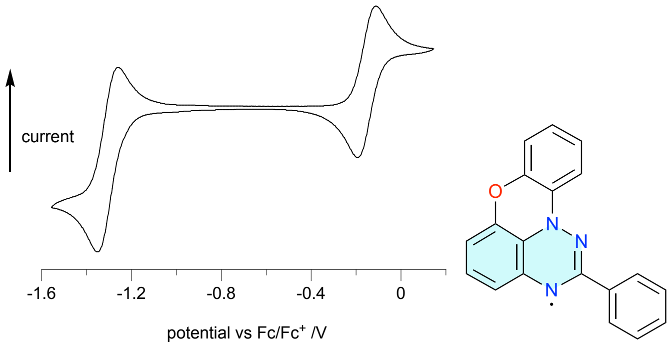

Figure S17. Cyclic voltammogram for parent radical **2a** in CH_2Cl_2 referenced to the Fc/Fc^+ couple.

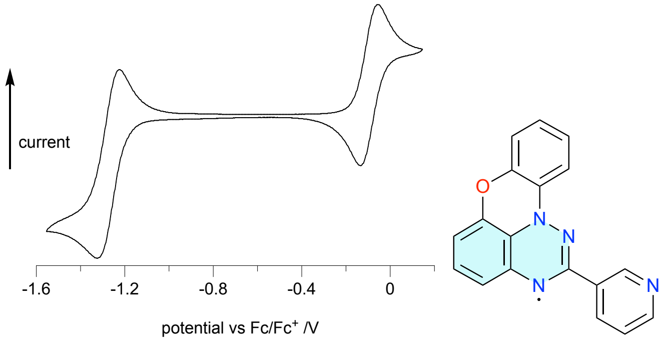

Figure S18. Cyclic voltammogram for pyridin-3-yl radical **2c** in CH_2Cl_2 referenced to the Fc/Fc^+ couple.

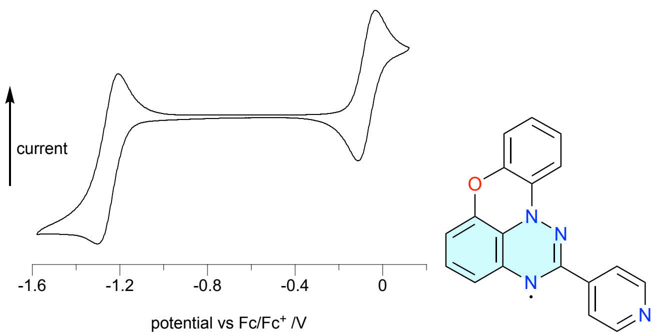

Figure S19. Cyclic voltammogram for pyridin-4-yl radical **2d** in CH_2Cl_2 referenced to the Fc/Fc^+ couple.

Table S2. Electrochemical data for radicals 2.

radical	$E_{1/2}^{-1/0}$ (V)	$E_{1/2}^{0/+1}$ (V)	E_{cell} (V)
**2a**	-1.306	-0.153	1.153
**2c**	-1.275	-0.096	1.179
**2d**	-1.255	-0.074	1.181

[a] Recorded in CH_2Cl_2 (1 mM). Conditions: $Bu_4N^+PF_6^-$ (100 mM), glassy carbon electrode, scan rate 50 mV·s^{-1}, *ca*. 20 °C. Potentials reported *vs* Fc/Fc^+.

6. EPR spectroscopy

EPR spectra for radicals **2c** and **2d** in degassed benzene and their Cu complexes in the solid-state were recorded on a X-band EMX-Nano EPR spectrometer at ambient temperature. The microwave power for each measurement was set in the linear range of the detector response (established with the Power Sweep program; Figures S20 and S21) with the modulation frequency of 100 kHz, modulation amplitude of 0.5 G_{pp} and spectral width of 100 G for radicals, and 900 G for copper complexes. The *g*-values were calculated using the formula: $g = h\nu/\beta H$), where ν is the frequency of the spectrometer, $\beta = 9.2740154\times10^{-24}$ JT^{-1}, and H is the centre of the EPR signal.

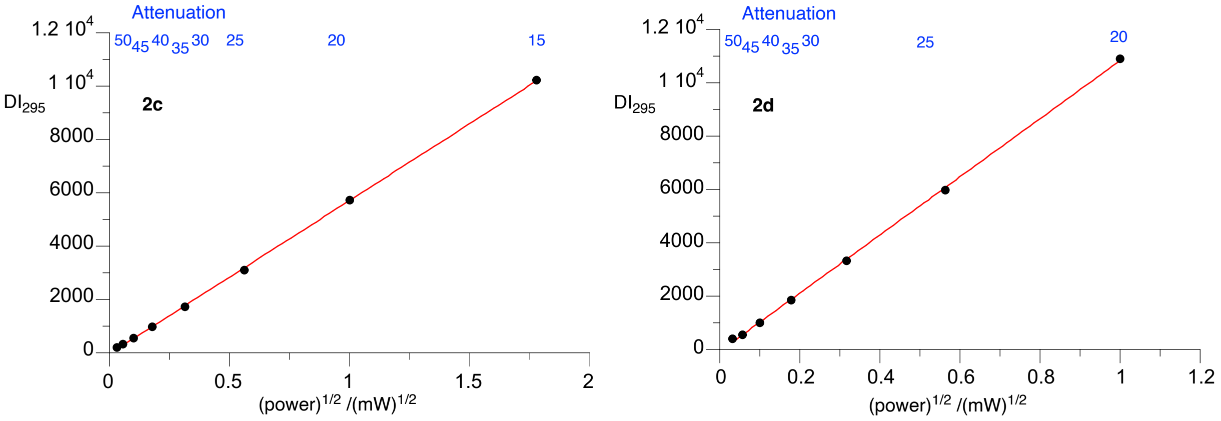

Figure S20. EPR double integral signal intensity (DI) *vs* the square root of microwave power for benzene solutions of radicals **2**. Left: **2c**, best fit line: DI_{295} = -45+5756×(power)$^{1/2}$, r^2 = 0.999; Right: **2d**, best fit line: DI_{295} = -64+10895×(power)$^{1/2}$, r^2 = 0.999.

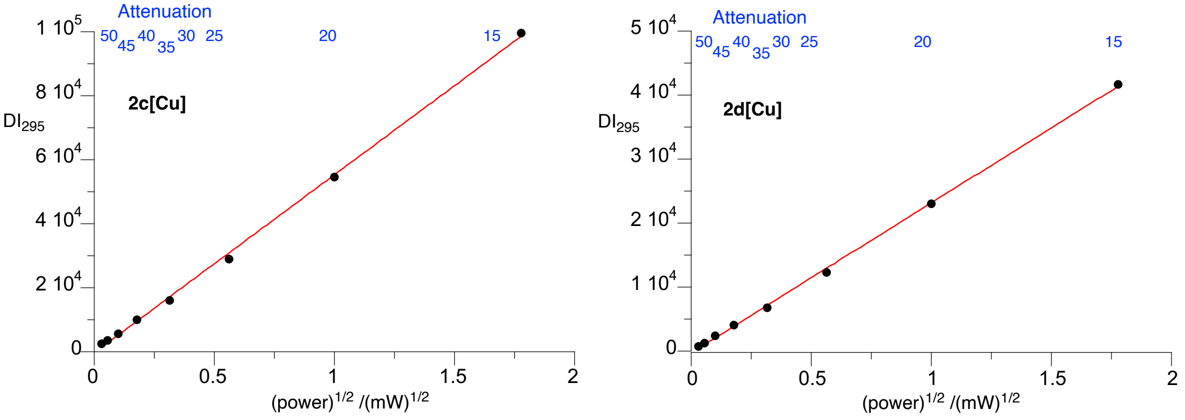

Figure S21. EPR double integral signal intensity (DI) *vs* the square root of microwave power for solid complexes **2[Cu]**. Left: **2c[Cu]**, best fit line: DI_{295} = -438+55636×(power)$^{1/2}$, r^2 = 0.999; Right: **2d[Cu]**, best fit line: DI_{120} = -34.79+1963×(power)$^{1/2}$, r^2 = 0.997.

Simulation of the first derivative of solution spectra of **2c** and **2d** was performed with *Winsim*,[6,7] using DFT results (*vide infra*) as the starting point including 3 nitrogen and 4 hydrogen atoms. The resulting *hfcc* values were perturbed several times until a global minimum for the fit was achieved. For consistency of results, previously recorded EPR spectra[8] of **2a** we re-simulated with the same method and parameters. Experimental and simulated spectra are shown in Figures S22–S27, while the resulting *hfcc* are collected in Table S3.

The resulting a_N *hfcc* values were correlated with the C(2) substituent Hammett σ_p parameters[9] and results are shown in Figure S28.

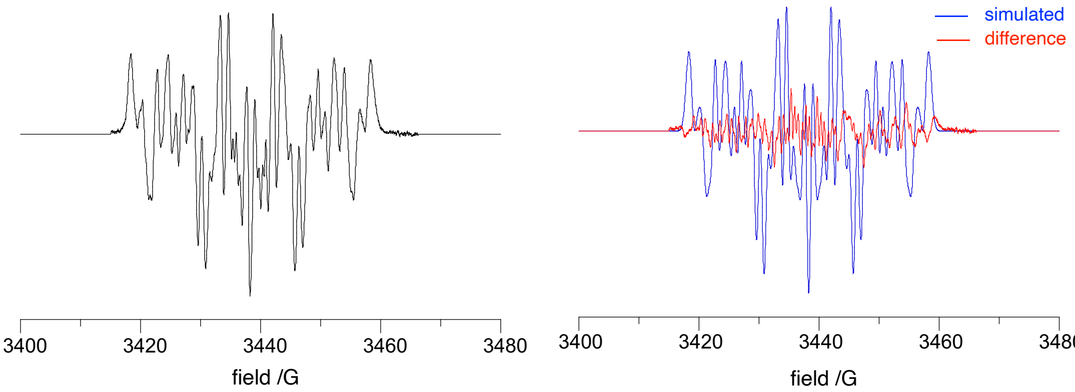

Figure S22. 1st derivative of experimental, recorded in benzene at 15 dB and *ca* 20 °C (black, left, ref [8]), simulated (blue, right), and difference (red, right) spectra for **2a**.

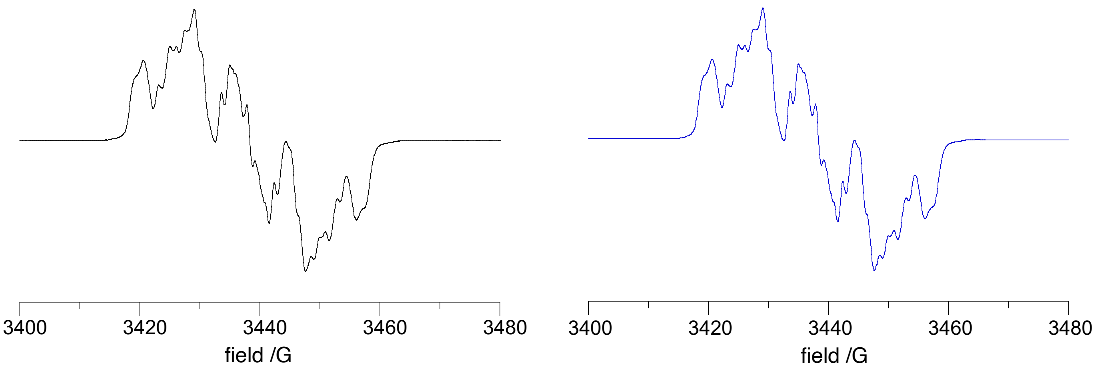

Figure S23. Experimental, recorded in benzene at 15 dB and *ca* 20 °C (black, left, ref [8]), and simulated (blue, right) spectra for **2a**.

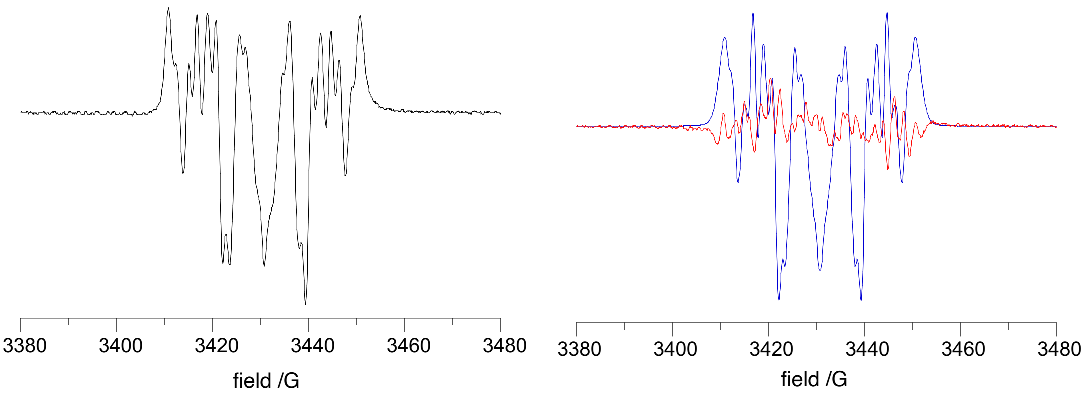

Figure S24. 1st derivative of experimental, recorded in benzene at 15 dB and *ca* 20 °C (black, left), simulated (blue, right), and difference (red, right) spectra for **2c**.

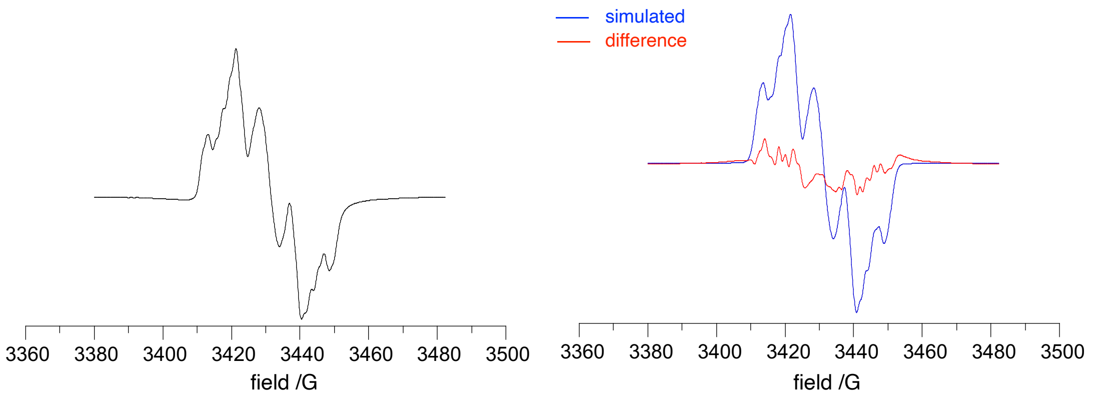

Figure S25. Experimental, recorded in benzene at 15 dB and *ca* 20 °C (black, left), simulated (blue, right) and difference (red, right) spectra for **2c**.

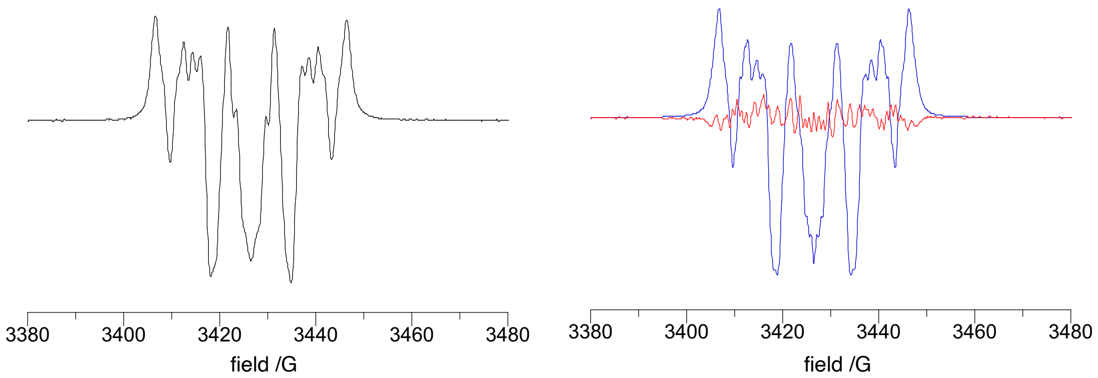

Figure S26. 1st derivative of experimental, recorded in benzene at 15 dB and *ca* 20 °C (black, left), simulated (blue, right), and difference (red, right) spectra for **2d**.

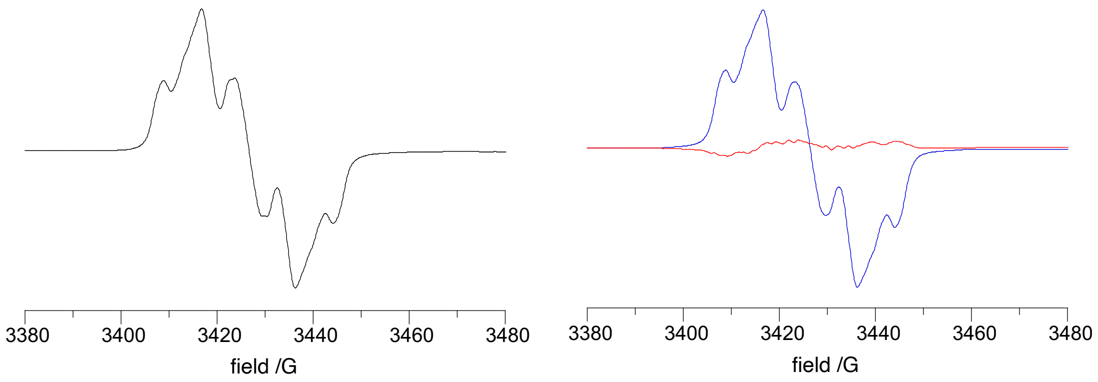

Figure S27. Experimental, recorded in benzene at 15 dB and *ca* 20 °C (black, left), simulated (blue, right), and difference (red, right) spectra for **2d**.

Table S3. Experimental hyperfine coupling constants (G) for radicals in benzene at *ca*. 20 °C.

atom	**2a** [a]	**2c**	**2d**
$a_{N(12)}$	7.43	7.54	7.53
$a_{N(1)}$	4.21	4.07	3.99
$a_{N(3)}$	4.47	4.42	4.51
a_H	1.93	1.80	1.89
a_H	1.90	1.66	1.80
a_H	1.14	1.59	1.11
a_H	0.84	0.80	0.77
g	2.0037	2.0055	2.0055

[a] Experimental data for simulation taken from ref [8]

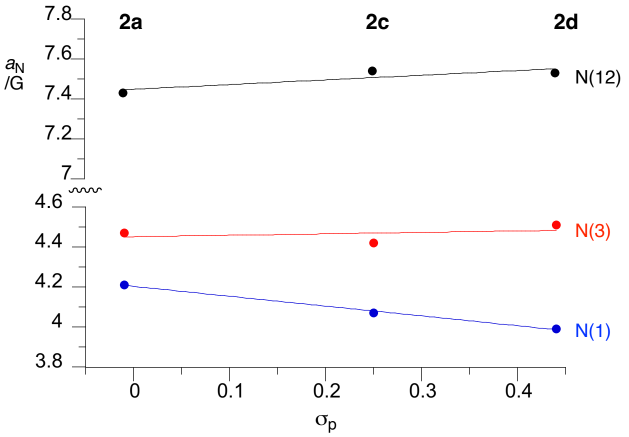

Figure S28. Correlation of the a_N *hfcc* for radicals **2** and the C(2) substituent Hammett parameter σ_p. Best fitting functions: $a_{N(12)} = 0.23(13)\times\sigma_p + 7.45(4)$, r^2=0.76; $a_{N(1)} = -0.49(3)\times\sigma_p + 4.20(1)$, r^2=0.996; $a_{N(3)} = 0.1(2)\times\sigma_p + 4.45(5)$, r^2=0.13.

The solid-state EPR spectra of complexes **2c[Cu]** and **2d[Cu]** were simulated using the *PIP v2.1* software[10] with *PIP4WIN* interface[11] applying Lorentzian line shape. Experimental and simulated spectra are shown in Figures S29 and S30, while the resulting g parameters for complexes in are listed in Table S4.

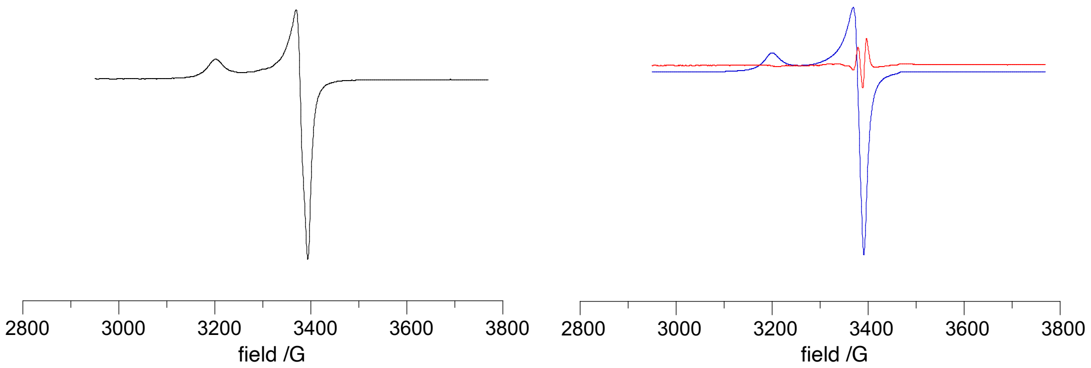

Figure S29. Experimental recorded in the solid state at 30 dB and at *ca* 20 °C (black, left), simulated (blue, right) and difference (red, right) spectra for **2c[Cu]**.

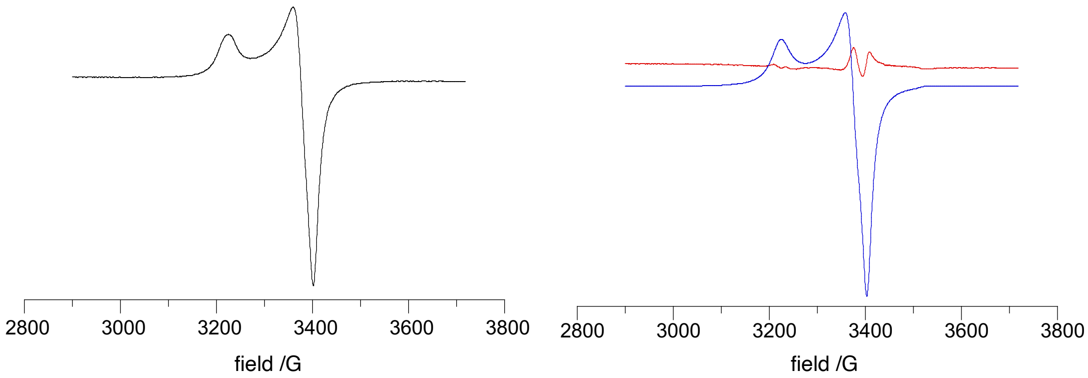

Figure S30. Experimental, recorded in the solid state at 30 dB at *ca* 20 °C (black, left), simulated (blue, right) and difference (red, right) spectra for **2d[Cu]**.

Table S4. Experimental parameters for solid-state complexes **2[Cu]** at *ca*. 20 °C.

	**2c[Cu]**	**2d[Cu]**
g_x	2.034	2.027
g_y	2.042	2.045
g_z	2.156	2.14
g_{iso}	2.077	2.071
$\Delta H_{pp(x)}$ /G	8	12
$\Delta H_{pp(y)}$ /G	10	17
$\Delta H_{pp(z)}$ /G	20	24

7. SQUID magnetometry of 2c[Cu]

The magnetic susceptibility of a polycrystalline sample of complex **2c[Cu]** mounted in a spherical polycarbonate capsule (Figure S31) was measured using a SQUID magnetometer (Quantum Design MPMS-XL-7T) and recorded in the temperature range of 300 K→ 2 K in an applied field of 0.6 T using a sweep rate of 1 K min^{-1}.

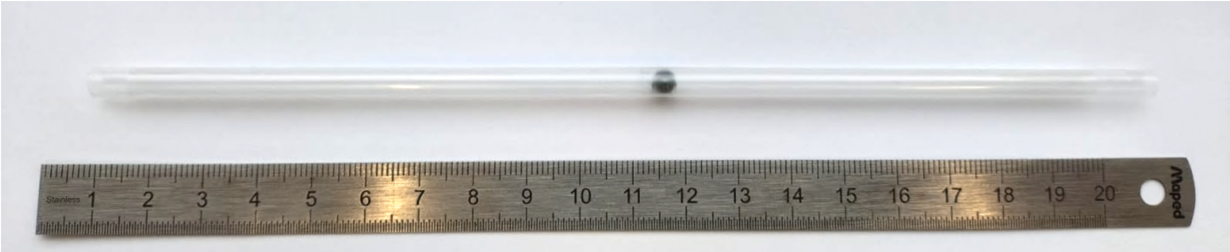

Figure S31. Sample contained in a spherical polycarbonate capsule and fitted to a plastic straw holder used as a sample holder in SQUID measurements.

Analysis of magnetic behavior of **2[Cu]** uses two models: Curie-Weiss (eq S1) and Hatfield[12] (eq S3) with Duffy and Barr[13] parameterization. The latter describes an alternating 1D Heisenberg antiferromagnetic chain based on the Hamiltonian in eq S2, where the secondary exchange interaction is proportional to the main interaction by factor α (alternation parameter). There are two sets of parameters A–F depending on the ratio of exchange interactions α.

$$\chi_p = \frac{C}{T-\theta} \qquad \text{hence} \qquad {}^{1}/_{\chi_p} = \frac{T}{C} - \frac{\theta}{C} \qquad \text{(eq S1)}$$

$$\hat{H} = -J \sum_{i=1}^{\frac{n}{2}} [S_{A_{2i}} S_{A_{2i-1}} + \alpha\, S_{A_{2i}} S_{A_{2i+1}}] \qquad \text{(eq 2)}$$

$$\chi_p(T) = \frac{N_A g^2 \mu_B^2}{k_B T} \frac{A+Bx+Cx^2}{1+Dx+Ex^2+Fx^3} \qquad \text{(eq 3)}$$

$$x = |J|/k_B T$$

For $0 \le \alpha \le 0.4$:

$A = 0.25$

$B = -0.062935 + 0.11376\alpha$

$C = 0.0047778 - 0.033268\alpha + 0.12742\alpha^2 - 0.32918\alpha^3 + 0.25203\alpha^4$

$D = 0.053860 + 0.70960\alpha$

$E = -0.00071302 - 0.10587\alpha + 0.54883\alpha^2 - 0.20603\alpha^3$

$F = 0.047193 - 0.0083778\alpha + 0.87256\alpha^2 - 2.7098\alpha^3 + 1.9798\alpha^4$

For $0.4 \le \alpha \le 1.0$:

$$A = 0.25$$

$$B = -0.068475 + 0.13194\alpha$$

$$C = 0.0042563 - 0.03167\alpha + 0.12278\alpha^2 - 0.29943\alpha^3 + 0.21814\alpha^4$$

$$D = 0.035255 + 0.6521\alpha$$

$$E = -0.00089418 - 0.10209\alpha + 0.87155\alpha^2 - 0.18472\alpha^3$$

$$F = 0.04523 - 0.008191\alpha + 0.83234\alpha^2 - 2.6181\alpha^3 + 1.92813\alpha^4$$

where J- exchange integral (the first interaction parameter), α- the alternation parameters ≤1 (αJ- the second interaction parameter).

To describe two antiferromagnetic chains in **2c[Cu]**, which account for two 1D Heisenberg magnetic chains composed of Cu(II) ions (J_{CuCu}) and radical ligands (J_{RR}), a sum of two Hamiltonians is used (eq S4)

$$\hat{H} = -J_{CuCu}[\hat{S}_{Cu1}\hat{S}_{Cu2} + \alpha_{CuCu}\hat{S}_{Cu2}\hat{S}_{Cu3}\ldots] - J_{RR}[\hat{S}_{R1}\hat{S}_{R2} + \alpha_{RR}\hat{S}_{R2}\hat{S}_{R3}\ldots] \quad \text{(eq S4)}$$

which leads to a composite Hatfield model (eq S5)

$$\chi_p(T) = \chi_{p(CuCu)}(T) + \chi_{p(RR)}(T) = \frac{N_A\mu_B^2}{k_BT}\left(g_{CuCu}^2\frac{A+Bx'+Cx'^2}{1+Dx'+Ex'^2+Fx'^3} + g_{RR}^2\frac{A+Bx+Cx^2}{1+Dx+Ex^2+Fx^3}\right) \quad \text{(eq S5)}$$

where *x'* is contains the term for exchange interaction between the Cu(II) ions and *x* between radicals.

Analysis for complex **2c[Cu]**

A microcrystalline sample of derivative **2c[Cu]** (m = 7.29 mg, 1.378×10^{-5} mol, M_w = 527.95 g mol^{-1} per XRD analysis for $C_{25}H_{14}CuN_5O_5$) in a polycarbonate capsule was analyzed at 0.6 T in the 300–2 K temperature range. Total molar magnetic susceptibility of the sample $\chi_{tot}(T)$ and $\chi_{tot}T(T)$ plots are shown in Figures S32 and S33, respectively.

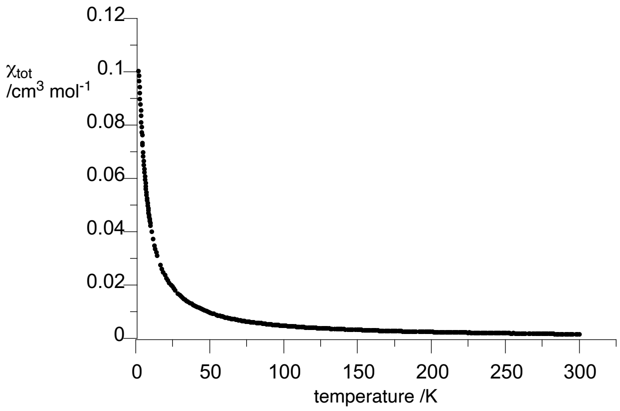

Figure S32. χ_{tot} *vs* T plot for **2c[Cu]** and the capsule.

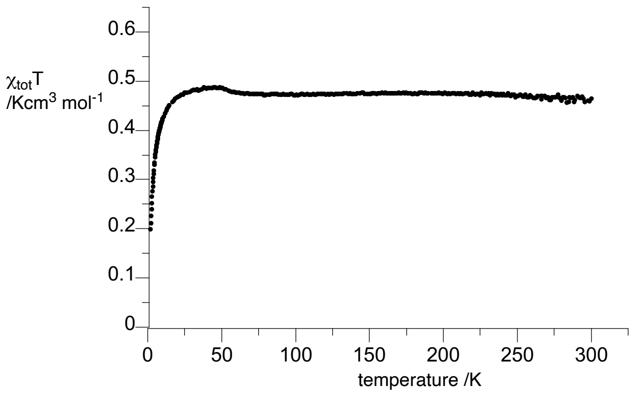

Figure S33. $\chi_{tot}T$ *vs* T plot for **2c[Cu]** and the capsule. The feature at 50 K is due to traces of paramagnetic oxygen trapped in the capsule with the sample.

A diamagnetic correction for the sample and the capsule was made using eq. S6.[14]

$$\chi_d = kM \times 10^{-6} \text{ /cm}^3 \text{ mol}^{-1} \qquad \text{eq S6.}$$

where M is the molecular weight (g mol^{-1}) and $k \sim 0.4 – 0.5$ cm^3 g^{-1}. The value of k was adjusted to provide *i*) a best fit to Curie-Weiss behavior ($\chi_p = C/(T - \theta)$) in the $T < 50$ K region with *ii*) a Curie constant $C = 0.405$ cm^3 K mol^{-1}, consistent with that expected for an S = ½ Cu(II) ion with $g_{iso} = 2.077$ (experimentally determined by EPR spectroscopy, Table S4). This afforded $k = 0.49$ ($\chi_d = -2.6 \times 10^{-4}$ cm^3 mol^{-1}) and an r^2 value of 0.9996.

The diamagnetic correction χ_d = -2.6 × 10^{-4} cm^3 mol^{-1} was used to obtain the paramagnetic susceptibility χ_p for **2c[Cu]**. The $\chi_p T$ *vs* T plot for **2c[Cu]** is shown in Figure S34 and Curie-Weiss plot in Figure S35.

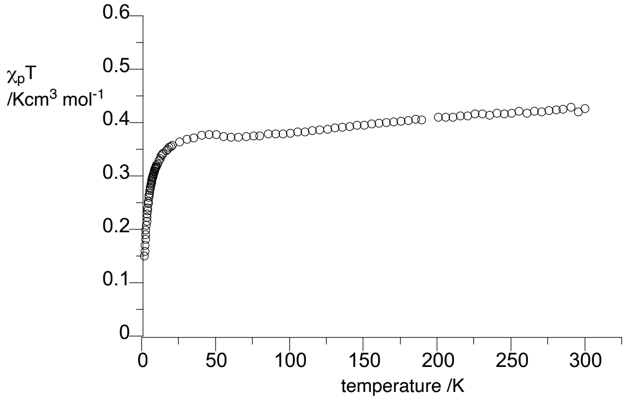

Figure S34. $\chi_p T$ *vs* T plot for **2c[Cu]**.

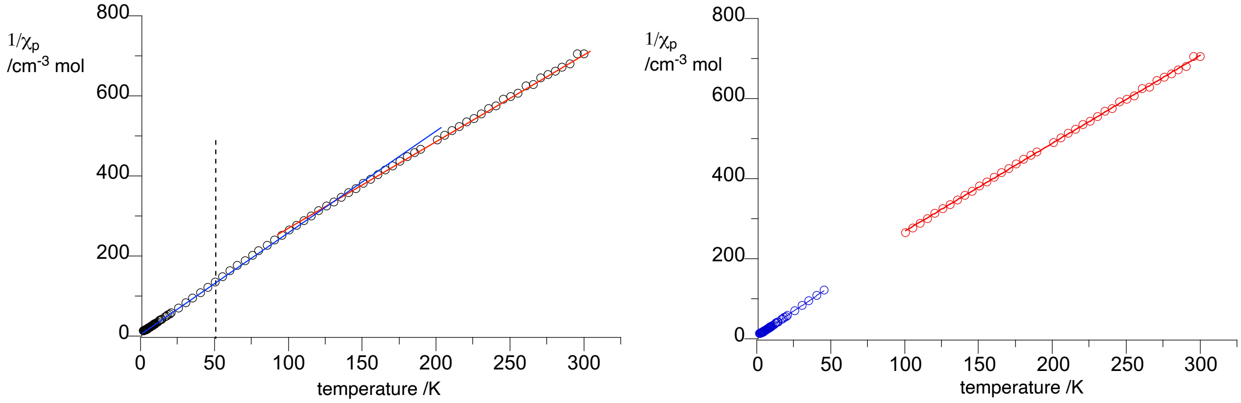

Figure S35. $1/\chi_p$ *vs* T plot for **2c[Cu]**. Left: The vertical line marks 50 K. The blue line shows the slope of linear data at low temperature and the red line at high temperature. Right: sections of the plot with the linear fitting to the Curie-Weiss formalism. Best fitting functions: low temperature (blue) $1/\chi_p$ = 2.483(7)×T+6.8(1), r^2=0.9996; high temperature (red) $1/\chi_p$ = 2.196(7)×T+48.8(16), r^2=0.9996.

The Curie-Weiss plot in Figure S35 exhibits two linear sections: below 50 K and above 100 K. The first is solely due to the Cu(II) paramagnetic behavior (blue line) and the second to the combined magnetic behavior of copper ions and the radical ligands. The resulting C parameter are C = 0.403(1) and 0.456(1) cm^3 mol^{-1}, and Weiss constants θ= -2.73(4) and -22(1) K.

To find exchange interaction values J_{CuCu} and J_{RR} and alternation parameters α_{CuCu} and α_{RR}, values $x' = |J_{CuCu}|/k_B T$ and $x = |J_{RR}|/k_B T$ in eq S5 were systematically varied (by assuming certain J values) in such a way that the predicted values χ_p in eq S5 gave best match for the experimental $1/\chi_p$ *vs* T data. Since the calculated α_{RR} = 0.40 (*vide infra*), two scenarios and sets of parameters were investigated for $\alpha > 0.4$ and for $\alpha < 0.4$.

The initial focus was on determining J_{CuCu} and α_{CuCu} assuming that below 50 K all paramagnetic moment is due to the Cu(II) ions, while radicals are EPR silent due to the predicted strong AFM exchange interactions. Therefore, eq S3 was used. Systematic variation of the x' value gave the best fit to the experimental $1/\chi_p(T)$ data for J_{CuCu}/k_B = -3.5 K and α_{CuCu} = 0.9. This provides the Cu(II) term, $\chi_{p(CuCu)}$, in eq S5. Further analysis gave the interaction between the radicals, J_{RR}/k_B = −1200 K and α_{RR} = 0.4. The plot of experimental χ_pT(T) with composite Hatfield fitting function is shown in the main text, while Figure S36 presents $\chi_p T$ *vs* log$T$ plot to accentuates the details of the fit.

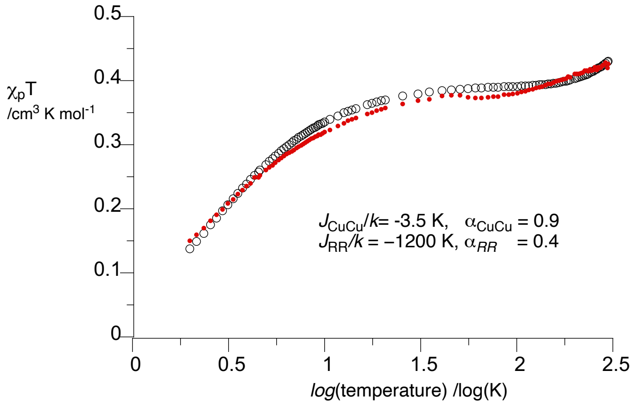

Figure S36. $\chi_p T$ *vs* log$T$ plot for **2c[Cu]** with fitting Hatfield model (red, eq 5) with the indicated parameters J and α.

8. Computational details and results

Quantum-mechanical calculations were carried out using Gaussian 16 suite of programs.[15] Geometry optimizations of the radicals **2** were obtained at the UB3LYP/6-311G(d,p) level of theory in vacuum, using tight convergence limits and with the C_s symmetry constrains.

a) isotropic Fermi contact coupling constants (hfcc) and spin densities for radicals

Isotropic Fermi contact coupling constants for radicals **2** were calculated using the UCAM-B3LYP/EPR-II // UB3LYP/6-311G(d,p) method in benzene dielectric medium requested with the SCRF(Solvent=Benzene) keywords (PCM model).[16] The resulting *hfcc* values are shown in Table S5 and spin densities are listed and graphically shown in Table S6. Correlations between the experimental and DFT hfcc values are shown in Figure S37.

Table S5. DFT calculated hyperfine coupling constants (G) of radicals **2** in benzene at the UCAM-B3LYP/EPR-II // UB3LYP/6-311G(d,p) level of theory.[a]

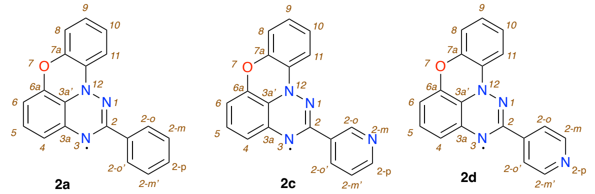

hfcc /G	**2a**	**2c**	**2d**
$a_{N(12)}$	5.91	6.00	6.02
$a_{N(1)}$	3.79	3.68	3.55
$a_{N(3)}$	4.14	4.12	4.22
$a_{H(4)}$	-0.58	-0.55	-0.59
$a_{H(5)}$	-0.82	-0.85	-0.81
$a_{H(6)}$	-0.83	-0.83	-0.89
$a_{H(8)}$	1.06	1.07	1.08
$a_{H(9)}$	-2.42	-2.43	-2.42
$a_{H(10)}$	1.14	1.14	1.14
$a_{H(11)}$	-2.49	-2.49	-2.47
$a_{H(2\text{-}o)}$	0.54	0.55	0.52
$a_{H(2\text{-}o')}$	0.53	0.49	0.50
$a_{X(2\text{-}m)}$	-0.30(C)	-0.29 (N)	-0.29(C)
$a_{H(2\text{-}m')}$	-0.33	0.18	-0.25
$a_{X(3\text{-}p)}$	0.45 (C)	0.48 (C)	-0.21 (N)

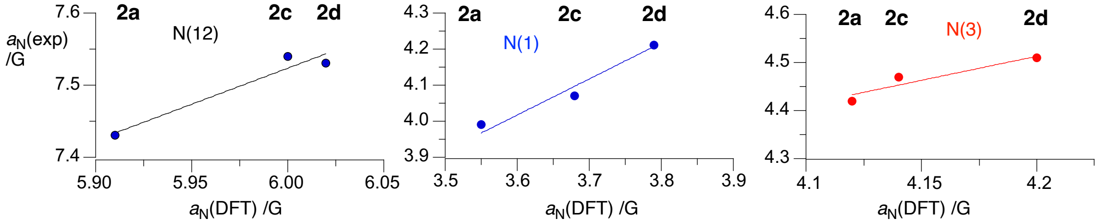

Figure S37. Plot of experimental vs DFT calculated a_N *hfcc* parameters for radicals **2**. Best fitting functions: $a_{N(12)}(\text{exp}) = a_{N(12)}(\text{DFT}) + 1.52(1)$, r^2=0.94; $a_{N(1)}(\text{exp}) = a_{N(1)}(\text{DFT}) + 0.42(1)$, r^2=0.98; $a_{N(12)}(\text{exp}) = a_{N(3)}(\text{DFT}) + 0.31(1)$, r^2=0.89.

Table S6. DFT calculated spin densities of radicals **2** in benzene at the UCAM-B3LYP/EPR-II//UB3LYP/6-311G(d,p) level of theory.[a]

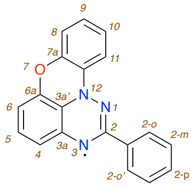
2a

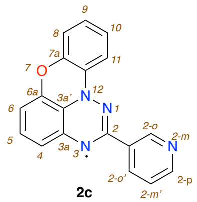
2c

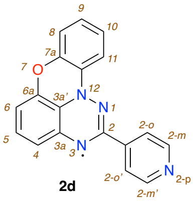
2d

Spin density	2a	2c	2d	graphic presentation *density = 0.008*
$\rho_{N(12)}$	0.281	0.284	0.284	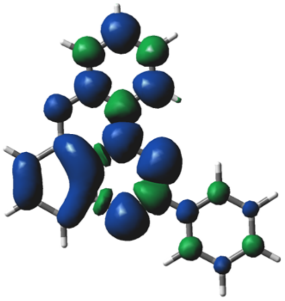
$\rho_{N(1)}$	0.280	0.273	0.265	
$\rho_{C(2)}$	-0.099	-0.098	-0.100	
$\rho_{N(3)}$	0.293	0.293	0.299	
$\rho_{C(3a)}$	0.001	0.003	0.001	
$\rho_{C(3a')}$	0.074	0.075	0.079	
$\rho_{C(4)}$	0.009	0.007	0.009	**2a**
$\rho_{C(5)}$	0.024	0.026	0.024	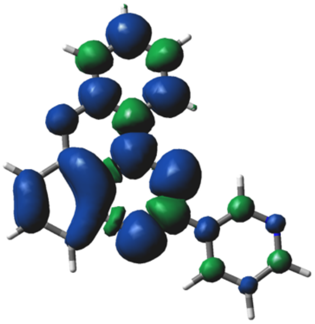
$\rho_{C(6)}$	0.023	0.023	0.025	
$\rho_{C(6a)}$	0.018	0.018	0.016	
$\rho_{O(7)}$	0.028	0.028	0.028	
$\rho_{C(7a)}$	0.079	0.080	0.080	
$\rho_{C(8)}$	-0.049	-0.050	-0.050	
$\rho_{C(9)}$	0.090	0.091	0.090	
$\rho_{C(10)}$	-0.050	-0.050	-0.049	**2c**
$\rho_{C(11)}$	0.087	0.087	0.086	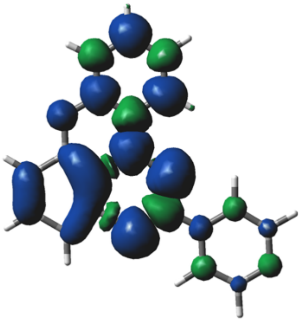
$\rho_{C(11a)}$	-0.075	-0.076	-0.075	
$\rho_{C(2\text{-}ipso)}$	0.020	0.018	0.018	
$\rho_{C(2\text{-}o)}$	-0.020	-0.020	-0.020	
$\rho_{C(2\text{-}o')}$	-0.020	-0.017	-0.019	
$\rho_{C(2\text{-}m)}$	0.012	0.012	0.012	
$\rho_{X(2\text{-}m')}$	0.013	N 0.011	0.010	
$\rho_{X(3\text{-}p)}$	-0.018	-0.018	-0.015	**2d**

b) spin delocalization in radicals in benzene dielectric medium

Spin delocalization parameter RDV (Radical Delocalization Value)[17] was calculated according to the formula (eq S6):

$$RDV = \sum_{i=1}^{n}(\rho_i)^2 \qquad \text{eq S6}$$

where spin concentration ρ_i on heavy atoms i (hydrogen atoms summed up to heavy atoms) is obtained with the UCAM-B3LYP/EPR-II // UB3LYP/6-311G(d,p) method in benzene dielectric medium requested with the SCRF(Solvent=Benzene) keywords (PCM model).[16] For the purpose of this work, an inverse is reported: $RDV^{-1} = 1/RDV$, since now larger values corresponds to greater delocalization. Results are shown in Table S7.

Table S7. Radical delocalization value (RDV^{-1}) for radicals **2**.

Radical	RDV^{-1} (in benzene)
**2a**	3.387
**2c**	3.414
**2d**	3.408

c) electronic excitations

Electronic excitation energies in CH_2Cl_2 dielectric medium were obtained at the UB3LYP/6-311G(d,p) // UB3LYP/6-311G(d,p) level of theory using the time-dependent TD-DFT method[18] supplied in the Gaussian 16 package. Solvation models in calculations were implemented with the PCM model[16] using the SCRF(solvent=CH_2Cl_2) keyword. Three lowest excitation energies, classified as π→π* transitions are listed in Table S8.

Energies of FMOs involved in the low energy transitions are listed in Table S9.

Table S8. Electronic transition energies and oscillator strength values with the indicated main electronic transition obtained at the TD-UB3LYP/6-311G(d,p) // UB3LYP/6-311G(d,p) level of theory in CH_2Cl_2 dielectric medium.

Radical	π→π* α-HOMO→ α-LUMO /nm (*f*)	π→π* β-HOMO→ β-LUMO /nm (*f*)	π→π* α-HOMO→α-LUMO+1 /nm (*f*)
**2a**	611.6 (0.003)	575.7 (0.051)	451.7 (0.42)
**2c**	631.4 (0.003)	578.4 (0.053)	449.1 (0.039)
**2d**	665.0 (0.005)	577.2 (0.054)	448.7 (0.040)

Table S9. Energies of MO involved in low energy transitions obtained from the UB3LYP/6-311G(d,p) // UB3LYP/6-311G(d,p) method in CH_2Cl_2 dielectric medium.

Radical	α-HOMO π /eV	α-LUMO π* /eV	α-LUMO+1 π* /eV	β-HOMO-1 π /eV	β-HOMO π* /eV	β-LUMO π* /eV
**2a**	-4.835	-1.745	-1.080	-6.895	-6.002	-2.938
**2c**	-4.904	-1.912	-1.126	-7.126	-6.067	-3.009
**2d**	-4.939	-2.065	-1.153	-7.289	-6.116	-3.048

d) partial output data from TD-DFT calculations for radicals 2

Method: UB3LYP/6-311G(d,p)// UB3LYP/6-311G(d,p)

Keywords: TD(nstates=20, root=1) SCRF(solvent=CH2CL2) SCF=tight

2a(Ar=Ph)

```
Excited State   1:  2.045-A'     2.0272 eV  611.60 nm  f=0.0030  <S**2>=0.796
     78A -> 79A         0.96004
     76B -> 78B        -0.11108
     77B -> 78B        -0.14197
 This state for optimization and/or second-order correction.
 Total Energy, E(TD-HF/TD-DFT) =  -970.854411652
 Copying the excited state density for this state as the 1-particle RhoCI density.

 Excited State   2:  2.087-A'     2.1535 eV  575.73 nm  f=0.0511  <S**2>=0.838
     78A -> 79A         0.13351
     78A -> 80A        -0.10957
     77B -> 78B         0.96336

 Excited State   3:  2.198-A'     2.7448 eV  451.71 nm  f=0.0417  <S**2>=0.958
     77A -> 80A         0.13162
     78A -> 80A         0.94342
     72B -> 78B         0.13039
     77B -> 80B        -0.10603

 Excited State   4:  2.048-A"     2.9615 eV  418.65 nm  f=0.0011  <S**2>=0.799
     73B -> 78B         0.98755

 Excited State   5:  2.484-A'     3.1496 eV  393.66 nm  f=0.0353  <S**2>=1.293
     75A -> 82A         0.10333
     76A -> 79A         0.14687
     77A -> 79A         0.32426
     78A -> 79A         0.12618
     76B -> 78B         0.84595
     76B -> 79B         0.14736
     77B -> 79B         0.20627
```

2c(Ar=3-Pyridyl)

```
Excited State   1:  2.040-A'     1.9636 eV  631.43 nm  f=0.0027  <S**2>=0.791
     78A -> 79A         0.96321
     77B -> 78B        -0.11528
 This state for optimization and/or second-order correction.
 Total Energy, E(TD-HF/TD-DFT) =  -986.896535182
 Copying the excited state density for this state as the 1-particle RhoCI density.

 Excited State   2:  2.085-A'     2.1437 eV  578.35 nm  f=0.0528  <S**2>=0.837
     78A -> 79A         0.10715
     78A -> 80A        -0.10798
     77B -> 78B         0.96791

 Excited State   3:  2.206-A'     2.7608 eV  449.09 nm  f=0.0390  <S**2>=0.966
     77A -> 80A         0.13634
     78A -> 80A         0.93892
     72B -> 78B         0.13452
     77B -> 80B        -0.11266

 Excited State   4:  2.053-A"     2.9626 eV  418.49 nm  f=0.0010  <S**2>=0.803
     73B -> 78B         0.69382
     75B -> 78B         0.70384

 Excited State   5:  3.308-A'     3.1760 eV  390.37 nm  f=0.0079  <S**2>=2.486
     75A -> 79A         0.19011
     77A -> 79A         0.54056
     78A -> 79A         0.10527
     78A -> 82A        -0.14880
     78A -> 83A         0.10979
     76B -> 78B         0.27229
     76B -> 79B         0.19670
     77B -> 79B         0.63026
```

2d(Ar=4-Pyridyl)

```
Excited State   1:  2.034-A'     1.8645 eV  664.98 nm  f=0.0051  <S**2>=0.785
     78A -> 79A         0.96991
 This state for optimization and/or second-order correction.
 Total Energy, E(TD-HF/TD-DFT) =  -986.900129345
 Copying the excited state density for this state as the 1-particle RhoCI density.

 Excited State   2:  2.087-A'     2.1481 eV  577.18 nm  f=0.0535  <S**2>=0.839
     78A -> 80A        -0.11003
     77B -> 78B         0.97128

 Excited State   3:  2.211-A'     2.7632 eV  448.70 nm  f=0.0396  <S**2>=0.972
     77A -> 80A         0.13859
     78A -> 80A         0.93892
     71B -> 78B         0.13621
     77B -> 80B        -0.11690

 Excited State   4:  2.055-A"     3.0017 eV  413.05 nm  f=0.0011  <S**2>=0.806
     73B -> 78B         0.98412

 Excited State   5:  3.340-A'     3.1604 eV  392.30 nm  f=0.0041  <S**2>=2.538
```

```
74A -> 79A        0.10416
77A -> 79A       -0.54386
77A -> 83A        0.12100
78A -> 81A        0.18688
78A -> 83A       -0.13982
71B -> 78B       -0.11994
72B -> 79B       -0.10193
77B -> 79B        0.69937
77B -> 83B       -0.12504
```

e) intermolecular interaction energy calculations

The spin-spin exchange interaction J_{DFT} in close pairs of spins (radical–Cu in the monomer, two radical ligands, and Cu···O···Cu bridge) in the crystal lattice of **2c[Cu]** were determined using the Yamaguchi formalism (eq S13):[19]

$$\Delta E_{ST} = J_{DFT} = 2\frac{E_{BS}-E_T}{\langle S^2\rangle_T-\langle S^2\rangle_{BS}} \qquad \text{eq S13}$$

where the SCF energies of the triplet (E_T) and broken symmetry singlet (E_{BS}) and total spin angular momenta $\langle S^2\rangle$ before spin annihilation were obtained by single point calculations at the UB3LYP/6-31++G(d,p) level of theory for selected pairs of ligands at the crystallographically determined coordinates. The oxygen atoms coordinating the axial positions of the Cu atoms were converted to H_2O ligands by addition of two H atoms at the idealized positions. For calculation of interactions in the Cu···O···Cu bridge, the radial was truncated leaving only the pyridine lingand with a hydrogen atom placed in the idealized position. The calculated energies and $\langle S^2\rangle$ values for the piars are shown in Table S10. The input geometries and calculated exchange interaction energies are shown in Figures S38–S40.

Table S10. Calculated energies and total spin angular momenta for monomeric **2c[Cu]**, radicals **2c**, and Cu–O bridges at their XRD coordinates.[a]

species	triplet energy /ht	$<S^2>_T$	open-shell singlet /ht	$<S^2>_{BS}$	ΔE_{ST} [b] /cal mol^{-1}
**monomer 2c[Cu]**	-3251.2205491	2.016795	-3251.2205407	1.016571	10.5
**monomer 2c[Cu]x2H_2O**	-3404.0929903	2.016123	-3404.0929821	1.015908	10.3
**2c radical pair 1**	-1973.1502246	2.025998	-1973.1510655	0.988317	-1016
**2c radical pair 2**	-1973.1483698	2.026164	-1973.1487011	1.012007	-410
**Cu…O…Cu bridge 1**	-5178.6315389	2.005246	-5178.631546	1.005186	-8.9
**Cu…O…Cu bridge 2**	-5178.6307322	2.005223	-5178.6307392	1.005158	-8.8

[a] Single point at the UB3LYP/6-31++G(d,p) level of theory at the XRD coordinates. [b] calculated according to eq. S13.

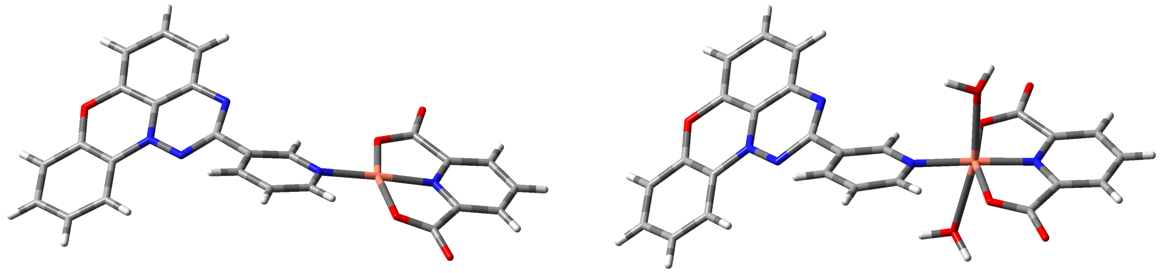

Figure S38. The monomeric unit of **2c[Cu]** without (left) and with two oxygen atoms (right) coordinating the Cu center in their crystallographic coordinates of the complex **2c[Cu]** used for DFT calculations of the S-T energy gap, E_{S-T}. The coordinating oxygen atoms were converted to water molecules. Data in Table S10.

pair 1 *pair 2*

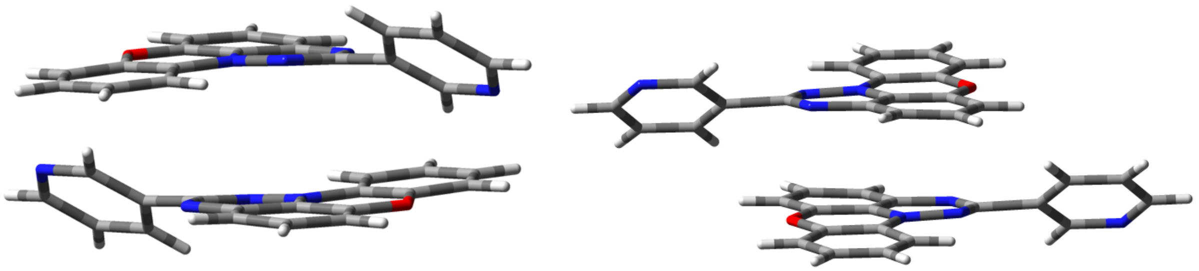

E_{S-T} (1) = –1.02 kcal mol^{-1} E_{S-T} (2) = -0.41 kcal mol^{-1}.

Figure S39. Two pairs of radical ligands **2c** in their crystallographic coordinates in the complex **2c[Cu]** used for DFT calculations of the S-T energy gap, E_{S-T}. Data in Table S10.

bridge 1 *bridge 2*

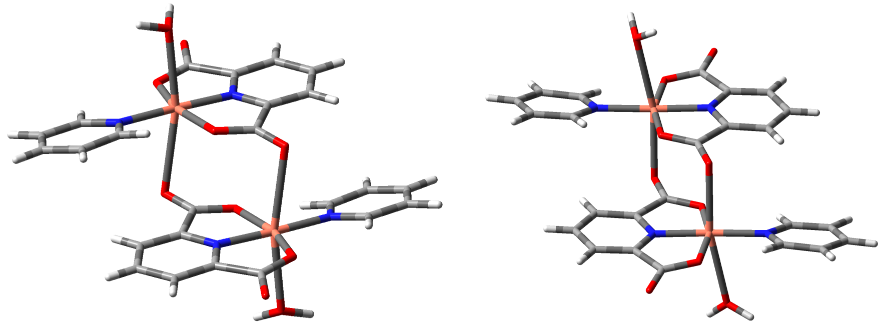

Figure S40. Two pairs of radical ligands **2c** in their crystallographic coordinates in the complex **2c[Cu]** used for DFT calculations of the S-T energy gap, E_{S-T}. Data in Table S10.

9. Archive for DFT geometry optimization results.

2a

1\1\GINC-LOCALHOST\FOpt\UB3LYP\6-311G(d,p)\C19H12N3O1(2)\PIOTR\07-Aug-
2024\0\\#P UB3LYP/6-311G(d,p) FOpt=tight freq(noraman) SCF=Direct #P G
eom=(NoDistance,NoAngle) fcheck\\Parent C(8)-O-Ph(N1) benzotrazinyl (p
henazinoBT), Cs\\0,2\N,-0.2278877873,0.,-0.7084263176\N,1.6321910991,0
.,1.3128825056\C,0.3129880577,0.,1.5111857835\N,-0.6503075981,0.,0.576
8055176\C,2.8786535524,0.,-2.6902290381\C,1.5291389331,0.,-2.380349485
6\C,1.1219316219,0.,-1.0442236245\C,2.0578378588,0.,0.0125543531\C,3.4
247554582,0.,-0.3201118186\C,3.816972262,0.,-1.6491147022\C,-1.1822310
988,0.,-1.7398126194\C,-0.7290760417,0.,-3.0714973973\C,-1.6371943064,
0.,-4.1201994823\C,-3.0046875126,0.,-3.8560366366\C,-3.459870238,0.,-2
.5383765587\C,-2.5557251842,0.,-1.4827298838\C,-0.1683642447,0.,2.9196
786726\C,-1.5360822218,0.,3.2244211726\C,-1.9623219845,0.,4.5480071083
\C,-1.0314057238,0.,5.5858369247\C,0.3306622795,0.,5.2903077622\C,0.76
0909599,0.,3.9678089319\O,0.6085245667,0.,-3.4016442722\H,4.1444535006
,0.,0.4880729973\H,-1.2511252521,0.,-5.131839092\H,-4.5225909432,0.,-2
.328713281\H,-2.8831618771,0.,-0.4536580141\H,-2.2565015446,0.,2.41782
91393\H,-3.0235523825,0.,4.77073054\H,1.0603303916,0.,6.0923893601\H,1
.8146738821,0.,3.72378903\H,4.8719986175,0.,-1.8962881704\H,3.17977092
88,0.,-3.7297147051\H,-3.7085837296,0.,-4.6792968653\H,-1.3657459375,0
.,6.617402166\\Version=ES64L-G16RevC.01\State=2-A"\HF=-970.9224068\S2=
0.765181\S2-1=0.\S2A=0.750177\RMSD=6.549e-09\RMSF=4.240e-06\Dipole=-0.
3140391,0.,-0.7901217\Quadrupole=6.2148761,-11.0265678,4.8116917,0.,-0
.4622123,0.\PG=CS [SG(C19H12N3O1)]\\@

2c

1\1\GINC-LOCALHOST\FOpt\UB3LYP\6-311G(d,p)\C18H11N4O1(2)\PIOTR\10-May-
2025\0\\#P UB3LYP/6-311G(d,p) FOpt=tight SCF=Direct #P Geom=(NoDistanc
e,NoAngle) fcheck\\2-(mPyridyl)C(8)-O-Ph(N1) benzotrazinyl, Cs\\0,2\N,
-0.0104202178,0.7505885539,0.\N,1.1284322656,-1.7514465045,0.\C,-0.184
9422738,-1.5253099383,0.\N,-0.8117873784,-0.3394565968,0.\C,3.55951384
33,1.6639967157,0.\C,2.1806041656,1.7905574572,0.\C,1.3772425679,0.647

8486079,0.\C,1.9383763178,-0.6476825943,0.\C,3.340176072,-0.7582304234
,0.\C,4.1268928988,0.382652904,0.\C,-0.5952732402,2.0284068914,0.\C,0.
2511817536,3.1518527248,0.\C,-0.2839853572,4.4317951655,0.\C,-1.665389
8201,4.6077759949,0.\C,-2.5095547387,3.4981999092,0.\C,-1.9803790532,2
.2131437325,0.\C,-1.0813633438,-2.710521807,0.\C,-2.4744006728,-2.5914
782445,0.\C,-3.2480997568,-3.7442594517,0.\C,-2.6049929529,-4.98019638
74,0.\C,-0.5404735242,-4.0042720427,0.\O,1.6246975976,3.0477033404,0.\
H,3.7718990398,-1.7505123424,0.\H,0.3986713547,5.272261654,0.\H,-3.584
5331041,3.6311111367,0.\H,-2.6134147045,1.3382823798,0.\H,-2.929816479
8,-1.6101621468,0.\H,-4.3304789357,-3.6906898182,0.\H,0.5360670613,-4.
124821166,0.\H,5.2063478953,0.2888758197,0.\H,4.1695720573,2.557872911
3,0.\H,-2.0770060395,5.6096325695,0.\H,-3.1809729718,-5.9017019697,0.\
N,-1.2755243251,-5.1180100347,0.\\Version=ES64L-G16RevC.01\State=2-A"\
HF=-986.9605303\S2=0.765144\S2-1=0.\S2A=0.750176\RMSD=4.704e-09\RMSF=1
.682e-06\Dipole=-0.287954,1.7788843,0.\Quadrupole=13.0339432,-6.234113
2,-6.7998301,1.4188701,0.,0.\PG=CS [SG(C18H11N4O1)]\\@

2d

1\1\GINC-LOCALHOST\FOpt\UB3LYP\6-311G(d,p)\C18H11N4O1(2)\PIOTR\10-May-
2025\0\\#P UB3LYP/6-311G(d,p) FOpt=tight SCF=Direct #P Geom=(NoDistanc
e,NoAngle) fcheck\\2-(para-Pyridyl)C(8)-O-Ph(N1) benzotrazinyl (phenaz
inoBT), Cs\\0,2\N,0.0025446677,0.7492412694,0.\N,1.1566039572,-1.74605
15409,0.\C,-0.1575519824,-1.525469482,0.\N,-0.7913239483,-0.345400555,
0.\C,3.5670741155,1.6843034283,0.\C,2.1870417343,1.802579007,0.\C,1.39
1247872,0.6548677766,0.\C,1.9606676535,-0.6371040948,0.\C,3.3626821458
,-0.7391014374,0.\C,4.142427294,0.406946387,0.\C,-0.5903273072,2.02370
20024,0.\C,0.249380256,3.1519442656,0.\C,-0.2933649397,4.428693257,0.\
C,-1.6757496676,4.5959496383,0.\C,-2.513307985,3.4812350486,0.\C,-1.97
65460326,2.1994453095,0.\C,-1.0481530613,-2.7196709321,0.\C,-2.4416997
44,-2.6087623555,0.\C,-3.207361155,-3.769980237,0.\C,-1.3596632807,-5.
1003535266,0.\C,-0.5012710643,-4.0055236044,0.\O,1.6238191238,3.055872
798,0.\H,3.8009261257,-1.7285163022,0.\H,0.3840397641,5.2733822828,0.\
H,-3.5890299491,3.6076412379,0.\H,-2.6043365414,1.320745546,0.\H,-2.91
46991298,-1.6365760144,0.\H,-4.2921132098,-3.7056516237,0.\H,-0.954630
6735,-6.1086230073,0.\H,0.5725496312,-4.1325416216,0.\H,5.2224308618,0
.319967003,0.\H,4.1715277796,2.5819768015,0.\H,-2.093567313,5.59522196
9,0.\N,-2.6931509977,-5.0042086929,0.\\Version=ES64L-G16RevC.01\State=
2-A"\HF=-986.9606498\S2=0.765155\S2-1=0.\S2A=0.750176\RMSD=7.046e-09\R
MSF=4.498e-06\Dipole=0.6183067,1.8632712,0.\Quadrupole=8.0443257,-3.10
8269,-4.9360567,-9.4015513,0.,0.\PG=CS [SG(C18H11N4O1)]\\@

10. References

(1) CrysAlisPro, 1.171.40.84a and 1.171.41.123a, Rigaku Oxford Diffraction, 2020 and 2022, Yarnton, Oxfordshire, England.

(2) Sheldrick, G. M. *SHELXT* – Integrated space-group and crystal- structure determination, *Acta Cryst., Sect. A* **2015**, *A71*, 3-8.

(3) Sheldrick, G. M. Crystal structure refinement with *SHELXL*, *Acta Cryst., Sect. C* **2015**, *C71*, 3-8.

(4) Dolomanov, O. V.; Bourhis, L. J.; Gildea, R. J.; Howard, J. A. K.; Puschmann, H. OLEX2: a complete structure solution, refinement and analysis program, *J. Appl. Cryst.* **2009**, *42*, 339-341.

(5) Bodzioch, A.; Pomikło, D.; Celeda, M.; Pietrzak, A.; Kaszyński, P. 3-Substituted benzo[e][1,2,4]triazines: Synthesis and electronic effects of the C(3) substituent, *J. Org. Chem.* **2019**, *84*, 6377−6394.

(6) Duling, D. R. Simulation of multiple isotropic spin-trap EPR spectra, *J. Magn. Res. B* **1994**, *104*, 105–110.

(7) O'Brien, D. A.; Duling, D. R.; Fann, Y. C., EPR spectral simulation for MS-Windows, Public EPR Software Tools NIEHS; Simulation of Multiple Isotropic Spin Trap EPR Spectra.

(8) Bartos, P.; Anand, B.; Pietrzak, A.; Kaszyński, P. Functional planar Blatter radical through Pschorr-type cyclization, *Org. Lett.* **2020**, *22*, 180–184.

(9) Hansch, C.; Leo, A.; Taft, R. W. Survey of Hammet substituent constants and resonance and field parameters, *Chem. Rev.* **1991**, *91*, 165–195.

(10) Nilges, M. Illinois EPR Research Centre.

(11) Rawson, J. M. University of Windsor, *PIP* for Windows: 2011.

(12) Hall, J. W.; Marsh, W. E.; Weller, R. R.; Hatfield, W. E. The Hatfield model for 1D Heisenberg altrnating chain, *Inorg. Chem.* **1981**, *20*, 1033-1037.

(13) Duffy, W.; Barr, K. P. Theory of alternating antiferromagnetic Heisenberg linear chains, *Phys. Rev.* **1968**, *165*, 647–654.

(14) Kahn, O. *Molecular Magnetism*; VCH Publishers: Cambridge, UK, 1993.

(15) Gaussian 16, M. J. Frisch, G. W. Trucks, H. B. Schlegel, G. E. Scuseria, M. A. Robb, J. R. Cheeseman, G. Scalmani, V. Barone, G. A. Petersson, H. Nakatsuji, X. Li, M. Caricato, A. V. Marenich, J. Bloino, B. G. Janesko, R. Gomperts, B. Mennucci, H. P. Hratchian, J. V. Ortiz, A. F. Izmaylov, J. L. Sonnenberg, D. Williams-Young, F. Ding, F. Lipparini, F. Egidi, J. Goings, B. Peng, A. Petrone, T. Henderson, D. Ranasinghe, V. G. Zakrzewski, J. Gao, N. Rega, G. Zheng, W. Liang, M. Hada, M. Ehara, K. Toyota, R. Fukuda, J. Hasegawa, M. Ishida, T. Nakajima, Y. Honda, O. Kitao, H. Nakai, T. Vreven, K. Throssell, J. A. Montgomery, Jr., J. E. Peralta, F.

Ogliaro, M. J. Bearpark, J. J. Heyd, E. N. Brothers, K. N. Kudin, V. N. Staroverov, T. A. Keith, R. Kobayashi, J. Normand, K. Raghavachari, A. P. Rendell, J. C. Burant, S. S. Iyengar, J. Tomasi, M. Cossi, J. M. Millam, M. Klene, C. Adamo, R. Cammi, J. W. Ochterski, R. L. Martin, K. Morokuma, O. Farkas, J. B. Foresman, and D. J. Fox, Gaussian, Inc., Wallingford CT. 2016,.

(16) Cossi, M.; Scalmani, G.; Rega, N.; Barone, V. New developments in the polarizable continuum model for quantum mechanical and classical calculations on molecules in solution, *J. Chem. Phys.* **2002**, *117*, 43–54.

(17) De Vleeschouwer, F.; Chankisjijev, A.; Yang, W.; Geerlings, P.; De Proft, F. Pushing the boundaries of intrinsically stable radicals: Inverse design using the thiadiazinyl radical as a template, *J. Org. Chem.* **2013**, *78*, 3151–3158.

(18) Stratmann, R. E.; Scuseria, G. E.; Frisch, M. J. An efficient implementation of time-dependent density-functional theory for the calculation of excitation energies of large molecules, *J. Chem. Phys.* **1998**, *109*, 8218-8224.

(19) Yamaguchi, K.; Takahara, Y.; Fueno, T.; Nasu, K. Ab initio MO calculations of effective exchange integrals between transition-metal ions via oxygen dianions: Nature of the copper-oxygen bonds and superconductivity, *Jpn. J. Appl. Phys.* **1987**, *26*, L1362.
